# Developing Strategies to Reduce Unnecessary Services in Primary Care: Protocol for User-Centered Design Charrettes

**DOI:** 10.2196/15618

**Published:** 2019-11-26

**Authors:** Mandi L Klamerus, Laura J Damschroder, Jordan B Sparks, Sarah E Skurla, Eve A Kerr, Timothy P Hofer, Tanner J Caverly

**Affiliations:** 1 Center for Clinical Management Research Department of Veterans Affairs Ann Arbor, MI United States; 2 Institute for Healthcare Policy and Innovation University of Michigan Ann Arbor, MI United States; 3 Medical School University of Michigan Ann Arbor, MI United States

**Keywords:** quality of health care, user-centered design, design thinking, overtesting, medical overuse, overtreatment

## Abstract

**Background:**

Overtreatment and overtesting expose patients to unnecessary, wasteful, and potentially harmful care. Reducing overtreatment or overtesting that has become ingrained in current clinical practices and is being delivered on a routine basis will require solutions that incorporate a deep understanding of multiple perspectives, particularly those on the front lines of clinical care: patients and their clinicians. Design approaches are a promising and innovative way to incorporate stakeholder needs, desires, and challenges to develop solutions to complex problems.

**Objective:**

This study aimed (1) to engage patients in a design process to develop high-level deintensification strategies for primary care (ie, strategies for scaling back or stopping routine medical services that more recent evidence reveals are not beneficial) and (2) to engage both patients and primary care providers in further co-design to develop and refine the broad deintensification strategies identified in phase 1.

**Methods:**

We engaged stakeholders in design charrettes—intensive workshops in which key stakeholders are brought together to develop creative solutions to a specific problem—focused on deintensification of routine overuse in primary care. We conducted the study in 2 phases: a 6.5-hour design charrette with 2 different groups of patients (phase 1) and a subsequent 4-hour charrette with clinicians and a subgroup of phase 1 patients (phase 2). Both phases included surveys and educational presentations related to deintensification. Phase 1 involved several design activities (mind mapping, business origami, and empathy mapping) to help patients gain a deeper understanding of the individuals involved in deintensification. Following that, we asked participants to review hypothetical scenarios where patients, clinicians, or the broader health system context posed a barrier to deintensification and then to brainstorm solutions. The deintensification themes identified in phase 1 were used to guide phase 2. This second phase primarily involved 1 design activity (*WhoDo*). In this activity, patients and clinicians worked together to develop concrete actions that specific stakeholders could take to support deintensification efforts. This activity included identifying barriers to the actions and approaches to overcoming those barriers.

**Results:**

A total of 35 patients participated in phase 1, and 9 patients and 7 clinicians participated in phase 2. The analysis of the deintensification strategies and survey data is currently underway. The results are expected to be submitted for publication in early 2020.

**Conclusions:**

Health care interventions are frequently developed without input from the people who are most affected. The exclusion of these stakeholders in the design process often influences and limits the impact of the intervention. This study employed design charrettes, guided by a flexible user-centered design model, to bring clinicians and patients with differing backgrounds and with different expectations together to cocreate real-world solutions to the complex issue of deintensifying medical services.

**International Registered Report Identifier (IRRID):**

RR1-10.2196/15618

## Introduction

### Background and Rationale

Many efforts to decrease low-value care (overuse) have focused on avoiding one-time diagnostic procedures or treatments, such as not treating acute sinusitis with antibiotics [1]. However, much of health care involves the *routine* use of medical services for chronic conditions or preventive services. Thus, developing effective strategies to motivate appropriate *deintensification*—the scaling back or stopping of routine medical services that more recent evidence reveals are not beneficial—is a key component of reducing overuse. Examples of deintensifying include decreasing the dose of oral sulfonylurea medications for diabetes management, reducing the frequency of cancer screening, or stopping routine testing such as carotid artery screening that is no longer supported by the evidence. Deintensifying unneeded and potentially harmful services would improve quality of care by decreasing patient’s exposure to harm [2]. Furthermore, deintensification has the potential to improve access to *necessary* services for those who need them the most [3]. Yet, research has shown that deintensification can be rare even when patients are at high risk for net harm [4-6].

Overuse is a *wicked problem* [7] with no easy solutions—and deintensification of routine care may prove even more challenging than attempts to reduce other types of low-value care. A long-standing challenge that applies equally to all types of overuse is that patients and the public may focus on small opportunities for improvement and ignore larger treatment risks [8-10]. In addition, patients and clinicians come to a health care encounter with their own knowledge and beliefs about the degree to which care is beneficial or appropriate, and each individual could be hesitant to deintensify for a variety of reasons [11]. These beliefs may be stronger in the context of long-term ongoing care and represent an even more challenging barrier for reducing this type of care compared with reducing a one-time test or treatment for a patient. Furthermore, without clear guidance on exactly when to deintensify ongoing care [2], lack of time and lack of communication tools may be even more important barriers to appropriate deintensification of services that have been a matter of routine practice for both the patient and clinician [12]. Finally, patients and clinicians are also embedded within larger health system contexts, with motivational structures and processes that influence care decisions. As many existing performance measures incentivize high-intensity care regardless of appropriateness [13], clinicians may feel compelled to continue with inappropriate treatment (eg, intensive glucose management) and be hesitant to adopt newer recommendations to deintensify.

Thus, even more than for reducing other types of low-value care, deintensifying care that is successfully delivered as a matter of routine for both the patient and clinician will likely require innovative, multifaceted solutions and an in-depth understanding of multiple perspectives—particularly perspectives of those on the frontlines of clinical care: patients and clinicians. Moreover, as deintensifying care presents difficult challenges at multiple levels, simultaneously deploying multiple interventions may be required. We believe that to overcome these challenges, policy makers will need to do more than elicit knowledge and attitudes about deintensification from stakeholders [14]. A promising strategy is to directly engage patients and clinicians in the actual *design* of strategies to implement deintensification. In this paper, we detail the ways in which we employed user-centered design (UCD) activities to develop patient- and clinician-generated solutions, focusing both on digital health and nondigital (ie, traditional or *offline*) health, to the complex problem of deintensifying routine medical care within primary care clinics. Applying design approaches to interventions in health care is becoming more popular, and a recent review found that design processes may result in more practical, acceptable, and effective interventions as compared with other expert-driven methods [15].

### Study Objectives

We employed design charrettes, guided by a flexible UCD model, to engage stakeholders in generating innovative strategies to support successful deintensification in primary care. (A charrette is defined as an intensive workshop or session in which key stakeholders are brought together to build off of each other’s best ideas and develop creative solutions to a particular problem [16-18].)

The specific aims of the study were as follows:

1. To engage patients in developing high-level deintensification strategies for primary care (patient design charrettes [phase 1]).
2. To engage both patients and primary care providers in further developing and refining the broad deintensification strategies identified in phase 1 (patient-clinician design charrette [phase 2]).

## Methods

### User-Centered Design Overview

UCD is a discipline that seeks to ground the characteristics of an innovation within in-depth information about the individuals who will use the innovation [16]. Working closely with consultants from the University of Michigan Stamps School of Art & Design [19], we employed a set of design activities to help patients and providers generate strategies for deintensification.

**Figure 1:**
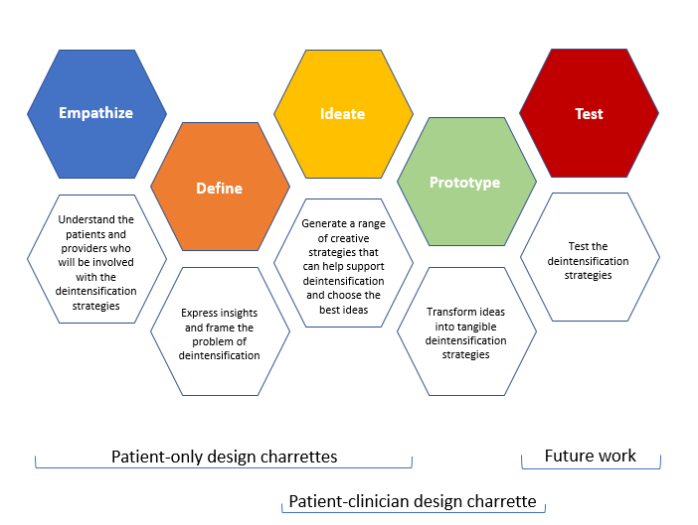
Design process model. Figure adapted, with permission, from Stanford d.school.

Design approaches prioritize deep empathy for end user desires, needs, and challenges to fully understand a complex problem in hopes of developing more comprehensive and effective solutions [20]. Design incorporates stakeholder needs and feedback throughout the co-design process and is increasingly being used in a variety of health care settings and conditions [15]. Although many variations of design process models exist, we selected the frequently used model developed by the Hasso Plattner Institute of Design at Stanford (also known as the d.school) to guide our work (Figure 1) [21].

The design model includes the following 5 stages:

1. Empathize: Work to understand the people who you are trying to find a solution for.
2. Define: Clearly articulate the primary problem (ie, what needs to be fixed).
3. Ideate: Brainstorm as many creative solutions as possible.
4. Prototype: Create representations of the solutions identified in the prior stage.
5. Test: Elicit feedback about the prototypes.

### Potential Benefits of Using a Design Approach to Develop Strategies for Deintensification

Design is a creative process to solve complex problems, such as the one addressed in this study: *stopping or reducing nonbeneficial medical services that have become part of a patient’s routine care*. We felt the following design approaches could support key goals for this project:

1. Participants would first be required to think through how other users involved in the deintensification process (eg, patients, providers, and caregivers) might feel about deintensification before beginning to develop solutions. This would guide participants toward a shared understanding of the users and ultimately more meaningful deintensification strategies.
2. Participants would consider the workflows in primary care, the competing demands and time constraints that providers confront during a clinic appointment, the preferences and motivations of users (primary care patients, primary care clinicians, and others), and other relevant issues. This would help ensure that the strategies generated would be particularly relevant to the primary care setting.
3. Participants would be encouraged to brainstorm as many creative solutions as possible and to think outside the box. Thus, at the end of the project, we would have an extensive list of potentially innovative strategies for deintensification.

In addition, by allowing us to directly engage those on the front lines of care delivery to generate potential solutions (patients and primary care clinicians), we felt that the strategies generated would be perceived as more practical, feasible, and trustworthy to other patients and clinicians on the front lines, increasing their dissemination and implementation potential.

### Study Design Overview

In an earlier part of the study, 37 recommendations for deintensification were validated by an expert panel using a modified RAND/UCLA Appropriateness Method [22]. These recommendations focused on common conditions and preventive care services encountered in adult ambulatory primary care. From these 37 recommendations, we reviewed deintensification recommendations that were rated highly by the expert panel. We selected 3 highly rated recommendations as topics for the charrettes, trying to identify a set of topics that are not only applicable to both genders but which might also elicit different concerns from participants (eg, cancer screening vs medications for cardiovascular prevention and diabetes treatment). The selected recommendations included the following:

1. Recommendation 1: Stop or decrease the dose of diabetes medications in patients aged 65 years and older who have low hemoglobin A1c (HbA_1c_<6.5%).
2. Recommendation 2: Do not do screening colonoscopy in average-risk adults aged 80 years or older. In addition, do not conduct screening colonoscopies more often than every 10 years.
3. Recommendation 3: Do not screen for carotid artery stenosis in asymptomatic adult patients without a history of cerebrovascular disease.

We conducted a design charrette with patients (phase 1; July 9, 2018) and repeated the charrette with a new group of patients (phase 1; July 14, 2018). Following these charrettes, 1 patient-clinician design charrette was conducted (phase 2; November 29, 2018). Phase 1 focused on the empathize, define, and ideate stages of our guiding design process model. Phase 2 focused primarily on the ideate and prototype stages. A future phase of the project will focus on the final stage, that is, the test stage.

The local Department of Veterans Affairs (VA) Institutional Review Board approved the study.

### Phase 1: Patient Design Charrette

#### Participant Recruitment

We stratified recruitment by gender and race to ensure a diversity of perspectives. Once a patient had been deemed conditionally eligible (see Multimedia Appendix 1 for inclusion and exclusion criteria), a staff member mailed the patient a recruitment letter explaining the study and informing them that a study team member would be calling to invite them to participate. A copy of the study consent form was included with the mailing. Approximately 1 week after the mailing, staff phoned the patient to explain the study and ascertain their interest in participating. (Staff attempted to contact a patient up to 3 times.)

For each phase 1 charrette, patients were recruited until approximately 30 agreed to participate (10 who met the eligibility criteria for Recommendation 1 plus 20 who met the eligibility criteria for Recommendations 2 and 3).

Approximately 2 weeks before a charrette, relevant materials were mailed to the patients who agreed to participate. These materials included information on the goals of the full research study, an explanation of what to expect during the charrette, a summary of the 3 recommendations that would be discussed at the session, and a map with driving directions to the session.

#### Design Charrette Overview

The phase 1 charrette lasted approximately 6.5 hours and was hosted at the VA Center for Clinical Management Research in Ann Arbor, Michigan. The registration process began by obtaining written informed consent. Once consent was obtained, participants were directed to their assigned group; each group focused on 1 of the 3 deintensification recommendations described above; 3 trained facilitators, 1 assigned to each group, guided the participants throughout the day (see Multimedia Appendix 2 for the facilitator’s guide). The participants completed a baseline survey (Multimedia Appendix 3).

Following a brief presentation by the project manager, to highlight the goals of the study and agenda for the day and introductions within groups, 8 design activities were conducted. The selected activities, which are commonly used in design charrettes, were assembled to help participants better understand the needs of clinicians, patients, and other clinical staff/leadership involved in the deintensification process, and to ground design of the deintensification strategies (the final product of the day) in information about the people who will ultimately be involved in carrying these strategies out in practice. Portions of the charrette were audiotaped.

#### Charrette Activities

The charrette activities supported broad, quick, and open idea generation. Imaginative, fresh, and creative ideas were encouraged (see Multimedia Appendix 4). Participants were asked to actively listen to others in their group and be open-minded and not critical (ie, “every idea is a good idea”). In addition, participants were instructed to go for volume (ie, “generate as many ideas as possible”).

#### Presentation by a Veterans Affairs Primary Care Physician

A VA primary care provider gave a brief presentation to orient participants to how doctors think about deintensification, highlight some of the challenges in deintensifying, and assure participants that deintensifying is often the right thing to do (ie, “appropriately deintensifying does mean that you are getting the best care possible”). In addition, the presentation highlighted the importance of patient input to develop innovative and effective deintensification strategies.

#### Presentation by a Veterans Affairs Patient

Following the provider’s presentation, a Veteran patient who receives his care at the Ann Arbor VA Medical Center gave a brief presentation to explain deintensification from a patient’s perspective, to provide support to the doctor’s presentation, and to help make the participants feel comfortable sharing their opinions. The Veteran patient met several times with the study team, before the charrette, to discuss and prepare content for the presentation.

#### Case Review

Each group was presented with a written case related to their deintensification topic (ie, related to the subgroup’s specific recommendation; see Multimedia Appendix 5). The case included a patient persona, a provider persona, and a fictional story about scaling back, told from both the patient and provider perspectives. The case highlighted some of the reasons why deintensification can be so challenging and provided inspiration for the *gamestorming* UCD activities detailed in the following sections [17,23].

During the case review, the facilitator narrated the patient and clinician personas, and a session participant volunteered to narrate the corresponding story sections. After reading the case, the facilitator asked participants to reflect on what they heard in the case.

#### Mind Mapping

Mind mapping is a visual thinking tool to help organize the information [17]. Through nonlinear groupings and branches, it connects and organizes information around a central subject, thus allowing participants to better understand the relationships that exist. Mind mapping was used early in the charrette to help jump-start the creative process.

During this activity, session participants identified information that stood out, articulated their interpretation of issues and concepts mentioned in the case, and discussed ideas sparked by the case (Multimedia Appendix 6). During this discussion, facilitators wrote the group’s comments on a flipchart, creating branches to represent words related to the central idea (ie, the story) and sub-branches to represent words that further expanded on the central idea. The mind mapping diagram remained on display throughout the entire session.

#### Business Origami

Business Origami is an activity that allows participants to collaboratively develop a physical representation of a system [17]. The aim of this activity is to help groups gain a deeper understanding of the people and things involved, the surrounding environment, and the interaction(s) between them.

In our design charrette, participants were instructed to imagine that the patient and provider in the case were meeting for a medical appointment. Participants were asked to think about what that medical appointment might look like and to map out the flow of the medical appointment between the patient and doctor in the case using 3-dimensional icons (ie, paper pop-up tokens); some icons were preprinted with potentially relevant actors (eg, doctor, nurse, and patient’s spouse), artifacts (eg, doctor’s computer, medicine, and educational materials), and places (eg, check-in station, waiting room, and doctor’s office), and some were blank to allow participants to add new ones if needed (Multimedia Appendix 6). The interactions between the tokens were represented by arrows drawn on the surface (horizontal white paper) with colored markers. The completed business origami model was displayed throughout the session.

#### Empathy Mapping

An empathy map is a collaborative design tool for discovering deeper insights about users, customers, or stakeholders [24]. The aim of empathy mapping is for participants to put themselves in the place of another person and understand their motivations and frustrations. The structure of an empathy map canvas often includes 4 quadrants representing the user’s external, observable world, and internal mindset.

For our session, the facilitator placed a large outline of a human head onto a flipchart. This head represented the patient in the case. (The name of the patient and several of his/her characteristics were written on the canvas.) Then, 4 quadrants were drawn out from the head representing the following: seeing, saying/doing, hearing, and thinking/feeling. Participants were asked to write down on sticky notes what they think the patient might be seeing, saying/doing, hearing, and/or thinking/feeling during the medical appointment where deintensification was being discussed. The facilitator placed the sticky notes on the appropriate quadrant of the map (Multimedia Appendix 6). Pains (fears, frustrations, and anxieties) and gains (wants, needs, hopes, and dreams) were also articulated and written at the bottom of the canvas. This activity was repeated with a second human head representing the doctor in the case. The canvases were displayed throughout the session.

#### Identifying Strategies Card Game

We developed recommendation-specific scenarios about different patients and their primary care providers. The scenarios were designed so patients and clinicians in the scenarios varied along a spectrum of combinations of *degree of resistance to deintensification*, from highly resistant to deintensification, somewhat resistant to deintensification, or not at all resistant to deintensification. Scenarios covered all combinations of patient and clinician types. Each of the scenarios included the following 5 pieces of information: (1) a brief patient description, (2) a brief clinician description, (3) wants/needs of the patient and/or clinician, (4) motivations/reasoning of the patient and/or clinician, and (5) barriers (patient, clinician, and/or system-level) to deintensification. Put together, these pieces expressed a *problem statement* that participants reviewed together [25]. Following the review, participants brainstormed solutions that could help solve the problem as they saw it for that scenario.

The following is an example of a deintensification scenario where the patient is highly resistant to deintensification and the provider is not at all resistant to successful deintensification:

- 1. Patient description: Arik is 74 years old and retired from the army, suffers from diabetes, recently transferred to the VA, with several prescriptions including insulin; most recent HbA_1c_ level is low.
- 2. Provider description: Dr. Stokes is a physician at a large VA Medical Center, has been seeing Arik for 1 year.
- 3-5. Problem statement: Dr. Stokes wants Arik to reduce his insulin (wants or needs information) because Arik’s HbA_1c_ level is low and current evidence suggests that a low HbA_1c_ can be harmful in older adults (motivation information) but Arik has been on insulin for many years and is scared his blood sugars will rise if he stops or reduces it, so Arik refuses (barriers information).

Multimedia Appendix 7 provides the full set of patient-clinician scenarios that the participants reviewed. Participants worked in pairs or trios to work through a card game where they were taken through each scenario one-at-a-time in a structured fashion, to identify barriers that might prevent the doctor or patient from scaling back, and to brainstorm solutions (ie, strategies) to overcome those barriers. The pairs/trios were asked to brainstorm as many strategies as possible, then work together to select the best 1 to 3 strategies from all those brainstormed. Participants were prompted with the following instructions as they worked through each scenario using a worksheet (see worksheet template in Multimedia Appendix 8): Solution 1 - The big problem that would prevent scaling back is (insert text); A solution that can solve the problem is (insert text). This was repeated for up to 2 additional solutions (ie, solutions 2 and 3) as desired by the participants.

Participants repeated the above until they finished all the scenarios or until time for the activity ran out.

#### Dot Voting

Dot voting is one of the simplest ways to collaboratively prioritize and converge upon agreed solutions [23].

Following the card game, all scenario worksheets generated by the group were displayed on a nearby wall and read aloud by the group’s facilitator. Each participant in the group was given 6 dot stickers and asked to place dots on the 6 strategies they felt were most important.

#### Charrette Wrap-Up

At the end of the charrette, participants completed a postsession survey. The survey was similar to the baseline survey but included questions related to the participant’s willingness to participate in phase 2 of the study. Participants received a US $125 gift card for taking part in the session.

#### Analysis

Following both patient design charrettes, project staff rapidly reviewed all prioritized deintensification strategies, along with the related facilitator notes and audio recordings. The team followed a consensus process to identify themes and group similar themes together. The resultant 6 themes were termed *super strategies* and used to guide the phase 2 patient-clinician design charrette (Table 1).

**Table 1:** Deintensification super strategy categories from phase 1.

Super strategy category	Example
Provide patient education through outreach^a^	Offer a group class to educate patients about deintensification
Educate patients using mass/social media^a^	Educate the public about deintensification using billboards, newspapers, or magazines
Provide education to providers^a^	Have mandatory trainings for clinic staff (eg, providers and nurses) on the newest overuse recommendations
Provide patient-centered care^b^	“Treat the patient as a person and not as a number”
Educate patients during an appointment^b^	Use decision aids to help a patient better understand the risks and benefits of scaling back
Offer alternatives to care^b^	Have providers consider doing more up front to build rapport and trust with the patient to help ensure success during future scaling back efforts

^a^Groups focusing on this strategy: diabetes treatment in high-risk patients; Screening for carotid artery stenosis in asymptomatic patients.

^b^Group focusing on this strategy: screening for colorectal cancer in older adults.

### Phase 2: Patient-Clinician Design Charrette

#### Patient Recruitment

A staff member mailed patients, who participated in phase 1 and met other inclusion criteria (see Multimedia Appendix 1 for inclusion and exclusion criteria), a recruitment letter explaining the patient-clinician design charrette and informing them that a study team member would be calling to invite them to participate. A copy of the study consent form was included with the mailing. Approximately 1 week after the mailing, the staff called the patient to ascertain their interest in participating (the staff attempted to contact a patient up to 3 times). Once a patient indicated they were interested in participating, the staff member reviewed the consent form with the patient and answered any questions they had.

Two weeks before the session, relevant materials were mailed to the patients who agreed to participate. These materials included information on the goals of the full research study, a summary of the patient-only session (including a table outlining 3 of the *super* (deintensification) strategies identified during that session), a description of what to expect during the patient-clinician session, and directions to the session. In addition, patients received an index card titled *Personal Experience with Deintensification*. The index card stated:

Describe a time when you went to your doctor wanting a specific test/treatment, your doctor persuaded you that NOT getting the test/treatment was the best thing to do, and in the end, you felt good about it.

Patients were instructed to complete the card before the session and bring it with them to the session.

#### Provider Recruitment

A staff member emailed providers a recruitment letter explaining the patient-clinician design charrette. The study consent form was attached to the email. Providers were instructed to review the consent form and reply to the email if they were interested in participating in the session (the staff sent up to 3 recruitment emails to providers).

Two weeks before the session, relevant materials were hand delivered to the providers who agreed to participate. These materials included information on the goals of the full research study, a summary of the initial patient sessions (including a table outlining 3 of the *super* (deintensification) strategies identified during that session), a description of what to expect during the patient-clinician session, and directions to the session. In addition, providers received an index card titled *Personal Experience with Deintensification*. The index card stated:

Describe a time when a patient came to the clinic wanting a specific test or treatment, but after some discussion you were able to persuade the patient that it really wasn’t in their best interest. Then, describe a few ways that patients have made these deintensification conversations easier for you in the past.

Providers were instructed to complete the card before the session and bring it with them to the session.

#### Design Charrette Overview

The 4-hour session took place at the VA Center for Clinical Management Research in Ann Arbor, Michigan. The registration process began by obtaining written informed consent. Once consent was obtained, participants were directed to their assigned group, which was led by a trained facilitator (see Multimedia Appendix 9 for the facilitator’s guide). As in phase 1, each group focused on 1 of the 3 deintensification recommendations mentioned in the study design overview section. In addition, each group concentrated on 3 of the 6 deintensification *super* strategies identified in phase 1 (Table 1).

To begin, all participants completed a baseline survey. Following a brief presentation by a study investigator to outline the agenda and goals for the day and to summarize the high-level deintensification strategies generated during the phase 1 session, each participant shared their personal experience(s) with deintensification using the prompts introduced on the previously mailed index card, as described above. (If a participant forgot their card, they were instructed to simply share any experience they have had in scaling back or stopping tests or treatments.) After this, each facilitator briefly reviewed the 3 *super* strategies that their group would be focusing on during the remainder of the session, answered any questions participants had about the strategies, and discussed the goal of the primary charrette activity, WhoDo. WhoDo was used in this study to help participants develop concrete actions that specific stakeholders can take to support deintensification efforts. Portions of the charrette activities were audiotaped.

#### Charrette Activities

WhoDo is a tool that helps to brainstorm, plan, and prioritize actions (see Multimedia Appendix 4).

We modified the tool to create a *WhoDo matrix* (see Multimedia Appendix 6) [23]. This matrix collected information not only on the stakeholders (*Who*) and their actions (*Do*), but also on potential obstacles to the action (*Barriers*) and approaches to overcome the barriers (*How to Overcome*).

Specific questions that were to be considered included the following:

1. Who: Who is involved in making deintensification happen? Who is the decision maker? Who has the needed resources? Whose support is needed?
2. Do: What do they need to do or do differently? What actions will build toward the big goal? (Each Do (action) had to be concrete and measurable.)
3. Barrier: What could get in the way of getting this (Do) done? What potential problems exist?
4. How to Overcome: What needs to happen to be able to overcome the barrier(s)?

In addition, we asked participants to consider stakeholders (*Who*) at 3 different levels. These levels included the primary care team level, the local VA level, and the national VA level. A list of potential stakeholders and/or their role at each level (eg, primary care team—provider, patient, and nurse; local VA level—director [leadership], pharmacists [specialists], social workers [support services], and clerk [administration]; national VA level—National Office to promote health or prevent disease, Veterans Service Organizations) was provided to each of the 3 groups. Facilitators stressed to participants that for deintensification efforts to be successful at any 1 level, they often need to be supported by other levels of the health care system. Each facilitator gave a brief example, at 1 of the levels, as a demonstration.

#### Step 1: Brainstorming of Who and Do

The initial step of the activity was a simple 30-min brainstorming session. Facilitators instructed participants to consider, within the 3 super strategy areas assigned to their group, what could be done to support deintensification (*Do*) and who would be needed to make it happen (*Who*). (Note: We refer to these collectively as *WhoDo*.) Facilitator 1 asked participants to write their *WhoDo* ideas on sticky notes and then share with the entire group, facilitator 2 collected information directly on a whiteboard as they were brainstormed by participants, and facilitator 3 used both of the above techniques to collect information. Facilitators worked to ensure that ideas were generated in each of the 3 super strategy areas.

#### Step 2: Selection of the Most Important WhoDo

Following the brainstorming, participants were asked to work together to identify the actions that would be most effective in supporting appropriate deintensification. Each group was instructed to identify the top 1 to 3 actions and to select the 1 action that they would like to use to start their first WhoDo matrix.

#### Step 3: Identification of Barriers (Barriers) and Solutions for Overcoming the Barriers (How to Overcome)

Participants selected the level (ie, primary care team, local VA, and national VA) for the top 1 to 3 actions, and the facilitator copied the prioritized action into a *WhoDo matrix*, which was presented on large sheets of white paper. Facilitators then asked participants to brainstorm potential barriers (*Barriers*) that could get in the way of the action (*Do*) actually happening. All barriers were documented within the WhoDo matrix. Once participants felt like their list of barriers was complete, they were instructed to select the one barrier that was likely the most important obstacle to making the *Who* and *Do* happen, and the facilitator highlighted this barrier in the matrix. Following that, participants were instructed to discuss solutions (*How to Overcome*) that could help overcome the biggest barrier. Again, all solutions were documented within the WhoDo matrix. Once the list of solutions was felt to be complete, participants were instructed to select the best solution, and the facilitator highlighted this solution in the matrix. The facilitator asked participants to then consider how the most important solution could be supported by the other 2 levels of the health care system. The participants completed the same steps as above (ie, determining *Who*, *Do, Barriers, and How to Overcome*) for one or both remaining levels.

#### Step 4: Selection of the Most Valuable WhoDo

Groups were instructed to select the most valuable *WhoDo* from all completed matrices. Participants were asked to consider the following 3 questions when making their decision: (1) *Which is most likely to lead to appropriate deintensification?* (2) *Which is most sustainable?* (3) *Which would be most acceptable to all stakeholders?* Once the most valuable WhoDo was identified, each facilitator shared it with all session participants.

#### Charrette Wrap-Up

At the end of the charrette, participants completed a postsession survey. Patient participants received a US $75 gift card for taking part in the session.

#### Analysis Plan (phase 1 and phase 2)

##### Categorizing Strategies

The first step in our analysis will be to categorize the strategies that were developed by participants during phase 1. Our analytic team (LD, JS, TC, MK, and SK) will use an inductive coding approach to create categories for the prioritized phase 1 strategies. The categories will focus on the actor or entity responsible for initiating the deintensification strategies. We list the categories here to convey the breadth of responses across different levels: doctor, patient, other staff, multilevel (within a health system), health system, and national. The team will refine the definitions for the categories while coding the prioritized phase 2 strategies. We will reconcile any outdated codes from phase 1 with our phase 2 codes to ensure continuity in our coding application. Once we finalize our coding scheme, we will follow a deductive approach to categorize the remaining (ie, nonprioritized) strategies for phases 1 and 2.

##### Developing Themes

Building on our initial work, members of the analytic team will independently review the strategies within each level and create a list of ideas for patterns across the strategies. The team will consider several questions while reviewing the strategies in each level, such as What are the similarities across the strategies? What is the common thread in all these ideas? For example, do the commonalities lie in *who* participants think should be involved, or *how* this process should happen? Finally, we will review the ideas as a group and distill them into themes to capture what we heard from participants in each phase.

##### Examining the Survey Data

Analysis of the survey data will include basic descriptive statistics of the main variables of interest.

## Results

### Recruitment

**Figure 2:**
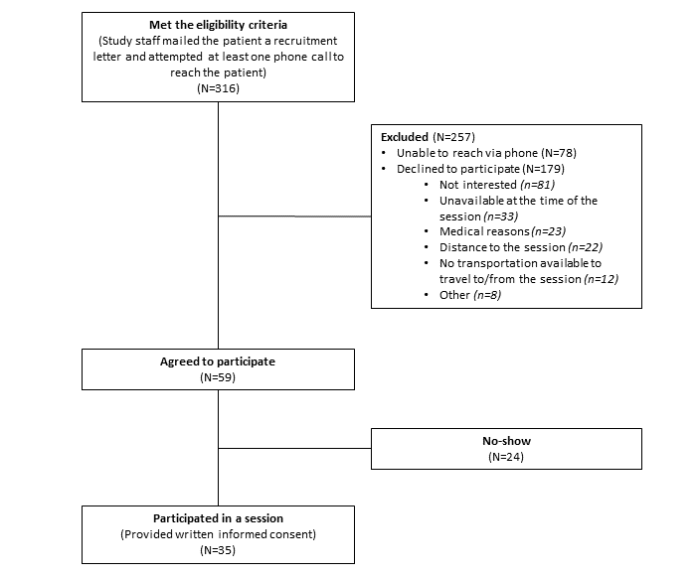
Recruitment for the two patient-only design charrettes (phase 1).

In phase 1, study staff sent recruitment letters and made at least one phone call attempt to 316 eligible patients (Figure 2).

Staff were unable to reach 78 patients via phone, and an additional 179 patients declined to participate in the study. Of the 59 patients verbally agreeing to participate, 35 provided written informed consent and participated in the study.

**Figure 3:**
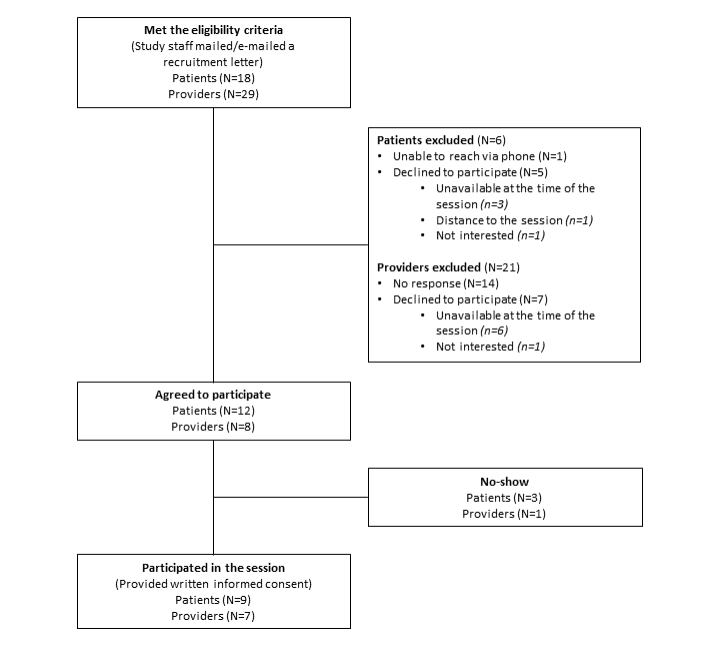
Recruitment for the patient-clinician design charrette (phase 2).

In phase 2, 18 eligible patients and 29 eligible providers received a study recruitment letter/email (Figure 3).

One patient could not be reached via phone, and 5 patients declined to participate; 14 providers did not respond to the recruitment email, and 7 providers declined to participate. Of the 12 patients who agreed to participate, 9 provided written informed consent and participated in the study. Of the 8 providers who agreed to participate, 7 provided written informed consent and participated in the study.

### Data Analysis

The categorization of deintensification strategies, development of themes, and analysis of survey data are currently underway. The results are expected to be submitted for publication in early 2020.

## Discussion

Our study protocol employs a novel method, design charrettes, to bring providers and patients with differing backgrounds and with different expectations together to cocreate solutions to the complex issue of deintensification. To our knowledge, this is the first study to use design charrettes, a collaborative session consisting of UCD activities guided by a UCD process model, to engage patients and providers in cocreating strategies to support successful deintensification in primary care.

Deintensification is closely connected to the concept of deimplementation. Deintensification occurs when a test or treatment is scaled back or stopped. This project focused on deintensification of routine services as exemplified in practice recommendations. Deimplementation is a similar concept that focuses on the broader need to develop system approaches to stop low-value practices [26] and can be seen as an implicit part of implementation and organizational change [27]. In the future, there may be deimplementation projects that focus on deintensification recommendations.

Design approaches have only recently been employed in health care, and the wide array of existing design processes have roots in disparate fields such as architecture, engineering, and business [28]. These approaches are now being taught in medical schools and are being used directly by doctors and nurses to improve patient care and patient’s experiences [29-31]. A concrete example of how design activities can have a real-world impact is the following: administrators at the Rotterdam Eye Hospital in the Netherlands wanted to transform the patient’s experience from an often anxiety-riddled episode into something more reliably pleasant and personal [32]. To do this, they incorporated UCD principles into their planning process. First, hospital staff set out to better understand their target user (ie, patients coming into the hospital for treatment). They found that most patients were scared about losing their eyesight; therefore, their primary goal was to reduce patients’ fears. The team brainstormed potential solutions. They sought insight from both inside and outside the health care field (eg, airlines, supermarkets, and other medical organizations). The most promising ideas were presented to the leadership of the hospital. Small-scale prototypes were tested, and the best ideas spread naturally. By using a design approach, the hospital was able to improve user experience. Patient intake increased by 47%, and the hospital has since won several awards for safety, quality, and design.

Other UCD success stories are summarized in a systematic review by Altman et al [15]. The authors examined how design has been used to plan interventions in health care settings and assessed whether the interventions were effective. They identified 26 papers, representing 24 interventions that used UCD in intervention development, intervention implementation, or both. A total of 19 of the interventions focused on physical health, 2 on mental health, and 3 on system processes. Although there were variable design activities employed across studies, all but one of the interventions showed positive effects on one or more outcomes.

By directly engaging patients and clinicians in the design process, the uncertainties and risks involved with innovation may be substantially minimized. Our study employs design to increase the chances that the resultant deintensification strategies are acceptable, effective, and sustainable in a primary care setting.

## Appendix

Multimedia Appendix 1 Inclusion and Exclusion Criteria (phase 1 and phase 2).

Multimedia Appendix 2 Phase 1 Facilitator Guide.

Multimedia Appendix 3 Phase 1 Survey.

Multimedia Appendix 4 User-Centered Design Charrette Activities and Associated Model Stage.

Multimedia Appendix 5 Case example.

Multimedia Appendix 6 Pictures of mind mapping, business origami, and empathy mapping.

Multimedia Appendix 7 Patient-clinician scenarios for the Identifying strategies card game.

Multimedia Appendix 8 Identifying strategies card game worksheet example.

Multimedia Appendix 9 Phase 2 Facilitator Guide.

Multimedia Appendix 10 Previous peer-review reports from the funder, the Department of Veterans Affairs.

## Abbreviations

HbA_1c_: hemoglobin A1c
UCD: user-centered design
VA: veterans affairs

## References

[1] Colla CH, Mainor AJ, Hargreaves C, Sequist T, Morden N (2017). Interventions aimed at reducing use of low-value health services: a systematic review. Med Care Res Rev.

[2] Kerr EA, Hofer TP (2016). Deintensification of routine medical services: the next frontier for improving care quality. JAMA Intern Med.

[3] Kale MS, Korenstein D (2018). Overdiagnosis in primary care: framing the problem and finding solutions. Br Med J.

[4] Maciejewski ML, Mi X, Sussman J, Greiner M, Curtis LH, Ng J, Haffer SC, Kerr EA (2018). Overtreatment and deintensification of diabetic therapy among medicare beneficiaries. J Gen Intern Med.

[5] McAlister FA, Youngson E, Eurich DT (2017). Treatment deintensification is uncommon in adults with type 2 diabetes mellitus: a retrospective cohort study. Circ Cardiovasc Qual Outcomes.

[6] Sussman JB, Kerr EA, Saini SD, Holleman RG, Klamerus ML, Min LC, Vijan S, Hofer TP (2015). Rates of deintensification of blood pressure and glycemic medication treatment based on levels of control and life expectancy in older patients with diabetes mellitus. JAMA Intern Med.

[7] Rittel HW, Webber MM (1973). Dilemmas in a general theory of planning. Policy Sci.

[8] Schwitzer G (2014). A guide to reading health care news stories. JAMA Intern Med.

[9] Squiers LB, Bann CM, Dolina SE, Tzeng J, McCormack L, Kamerow D (2013). Prostate-specific antigen testing: men's responses to 2012 recommendation against screening. Am J Prev Med.

[10] Torke AM, Schwartz PH, Holtz LR, Montz K, Sachs GA (2013). Older adults and forgoing cancer screening: 'I think it would be strange'. JAMA Intern Med.

[11] Morgan DJ, Brownlee S, Leppin AL, Kressin N, Dhruva SS, Levin L, Landon BE, Zezza MA, Schmidt H, Saini V, Elshaug AG (2015). Setting a research agenda for medical overuse. Br Med J.

[12] Powell AA, Bloomfield HE, Burgess DJ, Wilt TJ, Partin MR (2013). A conceptual framework for understanding and reducing overuse by primary care providers. Med Care Res Rev.

[13] Sirovich BE, Woloshin S, Schwartz LM (2011). Too Little? Too Much? Primary care physicians' views on US health care: a brief report. Arch Intern Med.

[14] Bartels LM, MacKuen MB, Rabinowitz G (2003). Democracy with attitudes. Electoral Democracy.

[15] Altman M, Huang TT, Breland JY (2018). Design thinking in health care. Prev Chronic Dis.

[16] Dopp AR, Parisi KE, Munson SA, Lyon AR (2018). A glossary of user-centered design strategies for implementation experts. Transl Behav Med.

[17] Martin B, Hanington BM (2012). Universal Methods of Design: 100 Ways to Research Complex Problems, Develop Innovative Ideas, and Design Effective Solutions.

[18] Lennertz B, Lutzenhiser A (2006). The Charrette Handbook: The Essential Guide for Accelerated, Collaborative Community Planning.

[19] University of Michigan STAMPS School of Art and Design.

[20] Roberts JP, Fisher TR, Trowbridge MJ, Bent C (2016). A design thinking framework for healthcare management and innovation. Healthc (Amst).

[21] Stanford d.school.

[22] Fitch K, Bernstein S, Aguillar M, Burnand B, LaCalle J, Lazaro P, van Het LM, McDonnell J, Vader J, Kahan J (2001). The RAND/UCLA Appropriateness Method User's Manual.

[23] Gray D, Brown S, Macanufo J (2012). Gamestorming: A Playbook for Innovators, Rulebreakers, and Changemakers.

[24] XPLANE.

[25] Devos J Toptal.

[26] Niven DJ, Mrklas KJ, Holodinsky JK, Straus SE, Hemmelgarn BR, Jeffs LP, Stelfox HT (2015). Towards understanding the de-adoption of low-value clinical practices: a scoping review. BMC Med.

[27] Wang V, Maciejewski ML, Helfrich CD, Weiner BJ (2018). Working smarter not harder: coupling implementation to de-implementation. Healthc (Amst).

[28] Dam R, Siang T Interaction Design Foundation.

[29] McLaughlin JE, Wolcott MD, Hubbard D, Umstead K, Rider TR (2019). A qualitative review of the design thinking framework in health professions education. BMC Med Educ.

[30] Kalaichandran A (2017). The New York Times.

[31] Trowbridge M, Chen D, Gregor A (2018). Teaching design thinking to medical students. Med Educ.

[32] van der Heijde R, Deichmann D (2016). Harvard Business Review.

