# Supplementary material for: Developing Strategies to Reduce Unnecessary Services in Primary Care: Protocol for User-Centered Design Charrettes
Source: JMIR Res Protoc. 2019 Nov 26;8(11):e15618. doi: 10.2196/15618 (PMC6904896; doi:10.2196/15618)
Supplement: Multimedia Appendix 1 [file resprot_v8i11e15618_app1.docx]

## PHASE 1: PATIENT DESIGN CHARRETTE

### Inclusion Criteria

Using VA’s Corporate Data Warehouse (CDW), we generated a random sample of eligible patients receiving care at the VA Ann Arbor Healthcare System. Patients were included if they were 65 years or older and an active VA patient. (Active patient was defined by having at least two primary care visits at the Ann Arbor VA Healthcare System between April 1, 2016, and March 31, 2018, and at least one outpatient visit between April 1, 2016, and March 31, 2017. The patient’s most recent two visits needed to be at the Ann Arbor VA Healthcare System.) We selected patients who were likely to experience the situations described in the recommendations. For Recommendation 1, patients were required to have one inpatient diabetes diagnosis, two or more outpatient diabetes diagnoses, or a prescription for an antihyperglycemic medication between April 1, 2016, and March 31, 2018. Patients considered under Recommendation 2 or 3 were required to have at least one colorectal cancer screening test (i.e., colonoscopy, sigmoidoscopy, fecal immunochemical test (FIT), or fecal occult blood test (FOBT)) between April 1, 2013, and March 31, 2018.

### Exclusion Criteria

Patients were excluded if CDW data included any of the following diagnoses between April 1, 2013, and March 31, 2018: serious mental illness (including schizophrenia, schizoaffective disorder, bipolar disorder, or major depressive disorder with psychotic features); personality disorder; suicidal ideation; substance use disorder; end stage disease (i.e., terminal cancer); dementia or Alzheimer’s disease; stroke; or transient ischemic attack. Patients were also excluded if they didn’t have a phone number on record, resided in a nursing home, or were receiving palliative/hospice care. Additionally, patients considered under Recommendation 1 were excluded if they were on metformin only and patients considered under Recommendations 2 and 3 were excluded if they had a history of colorectal cancer, inflammatory bowel disease, or colectomy.

## PHASE 2: PATIENT-CLINICIAN DESIGN CHARRETTE

### Inclusion Criteria

A subset of patients from Phase 1 were invited to participate in Phase 2. Patients were included in the study sample if all of the following criteria were met: 1. Patient had previously participated in a Phase 1 patient-only design charrette; 2. Patient had indicated on the Phase 1 survey that they would be interested in continuing work on this topic or, for those who did not complete a survey, had verbally indicated to study staff during the patient-only session that they would be interested in participating in the Phase 2 patient-clinician design charrette; and 3. Patient was recommended by their assigned Phase 1 facilitator because the patient had been an active participant, contributing significantly in that session.

A list of primary care clinicians (physicians, nurse practitioners, and physician assistants) having at least 2 days of primary care clinic a week at the VA Ann Arbor Healthcare System was obtained from the Department of Ambulatory Care. These clinicians were included in the study sample.

### Exclusion Criteria

Patients who participated in a Phase 1 patient-only design charrette were excluded if they did not indicate interest in participating in Phase 2. A primary care clinician was excluded if s/he did not have at least 2 days of clinic each week, was an investigator on the current study, or was a medical resident.
