# Supplementary material for: Developing Strategies to Reduce Unnecessary Services in Primary Care: Protocol for User-Centered Design Charrettes
Source: JMIR Res Protoc. 2019 Nov 26;8(11):e15618. doi: 10.2196/15618 (PMC6904896; doi:10.2196/15618)
Supplement: Multimedia Appendix 2 [file resprot_v8i11e15618_app2.docx]

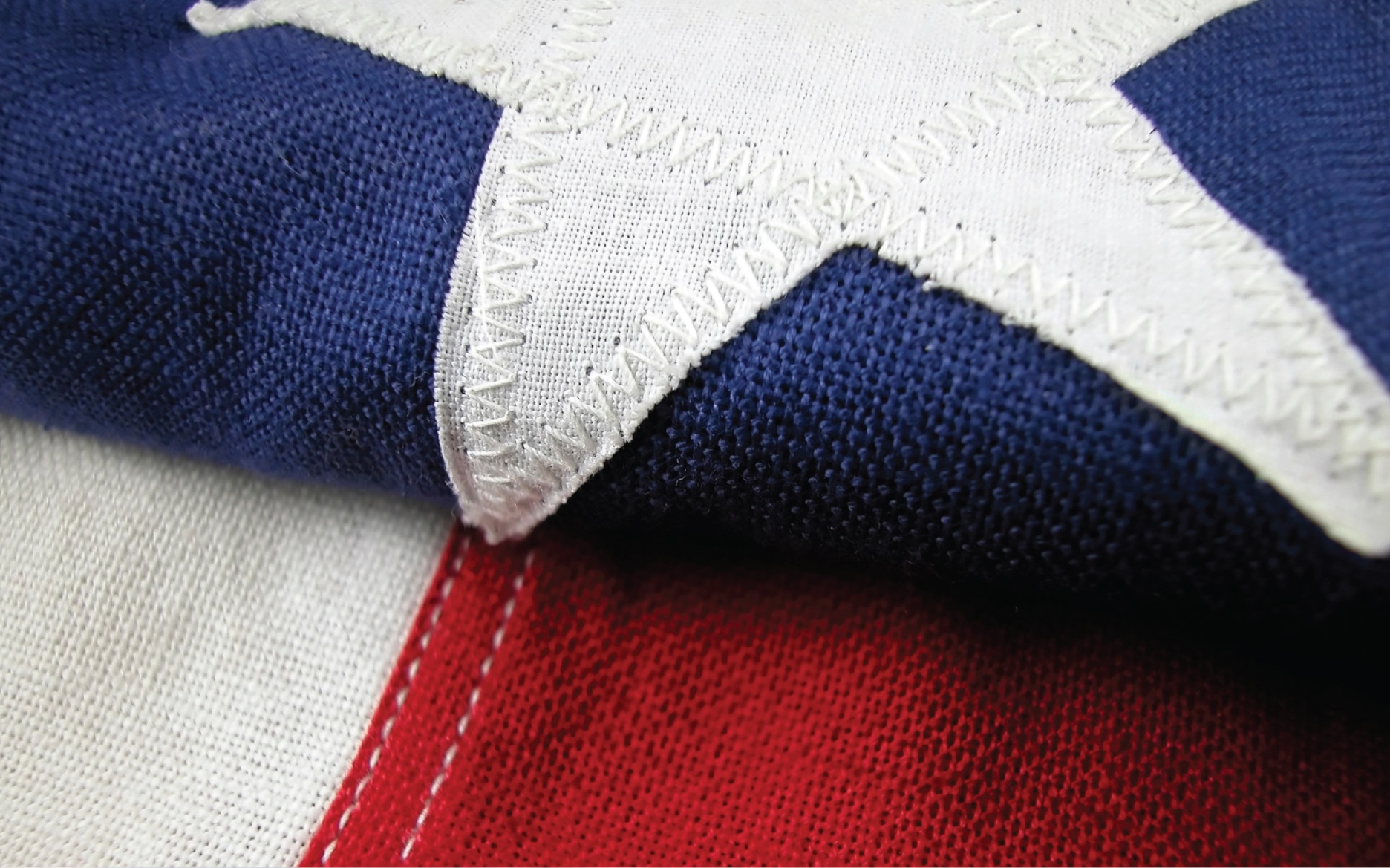


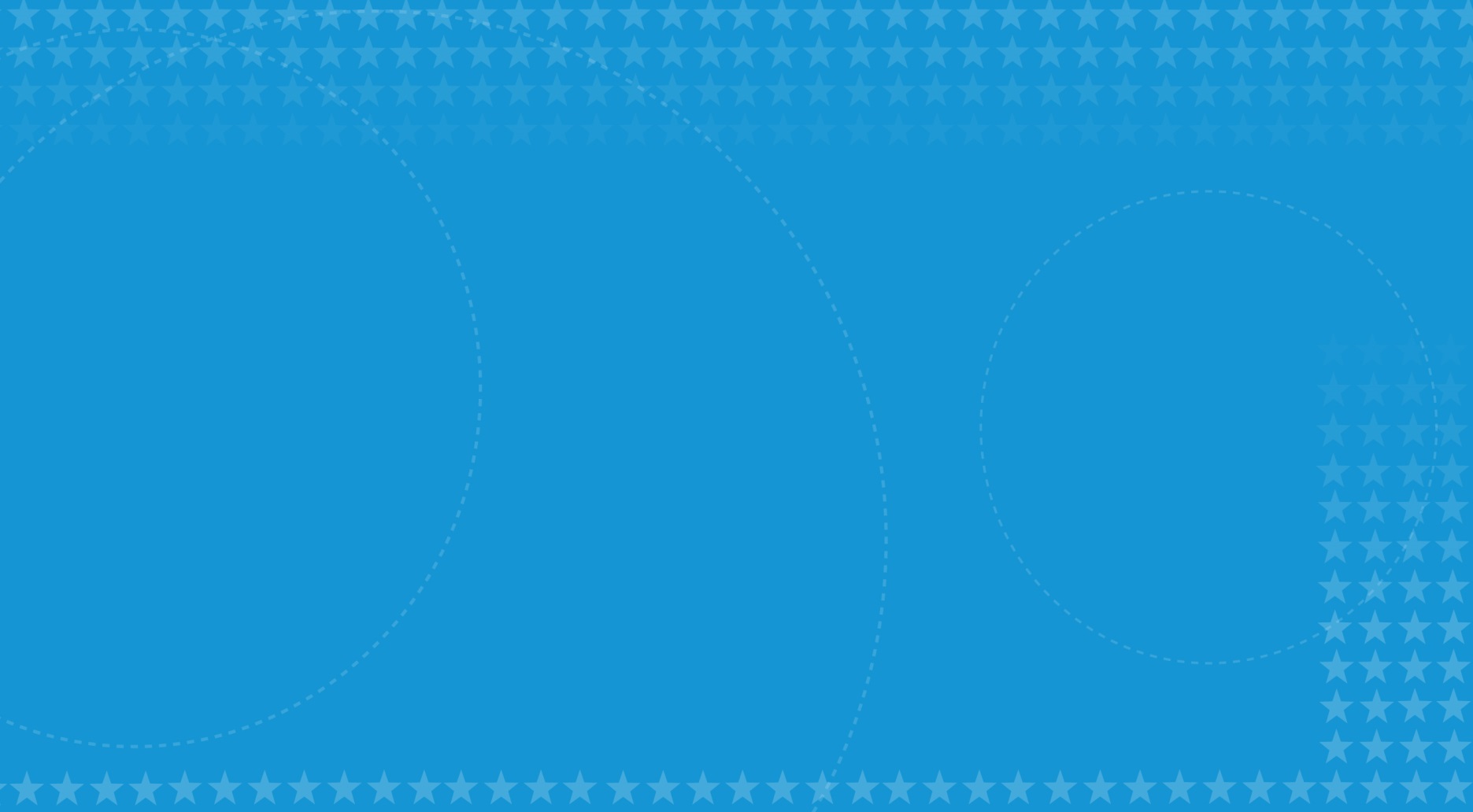
**Agenda for Facilitators**

**Patient Collaborative Design**

**Facilitator Guide**

| **Time** | **Activity** |
| --- | --- |
| 8:15 – 9:00 | Registration, Consent, and Pre-Forum Survey |
| 9:00 – 9:15 | Welcome, Overview, and Agenda |
| 9:15 – 9:35 | Introductions within Sub-Groups |
| 9:35 – 9:45 | Presentation #1: VA Doctor |
| 9:45 – 9:55 | Presentation #2: VA Patient |
| 9:55 – 10:05 | Q&A, Stretch Break, and Transition to Activities (10 min) |
| 10:05 – 10:15 | Case Review   - Sub-groups read case |
| 10:15 – 10:30 | Mind-Mapping   - Sub-groups visualize and summarize the facts of the case |
| 10:30 – 10:50 | Business Origami   - Using 3D icons, sub-groups imagine that the patient and doctor in the case are meeting for an appointment; sub-groups map out the interactions/conversations/environment |
| 10:50 – 11:00 | Break (10 min) |
| 11:00 – 11:40 | Empathy Maps: Patient and Doctor   - Sub-groups identify what the characters in the case are thinking/feeling, hearing, seeing, and saying/doing |
| 11:40 – 12:20 | Lunch (40 min) |
| 12:20 – 1:50 | ‘Identifying Solutions’ Card Game   - Participants work in pairs to identify scaling-back strategies to specific scenarios (includes strategy/solution prioritization) |
| 1:50 – 2:45 | Synthesis and Prioritization   - Participants select the strategies that are most important from all strategies identified in their sub-group |
| 2:45 – 3:00 | Thank-you |
| 3:00 – 3:30 | Post-Forum Survey, Gift Cards, and Consents |

| **Facilitation Tips** | |
| --- | --- |
| **If group members…** | **Then you might…** |
| Contribute relevant ideas | - Recognize contribution and continue moving discussion forward. |
| Seem unfocused/are goofing around   - People become undisciplined when they are overloaded or worn out. | - Ask the group to **take a break**, “let’s take a break.” - Add positive statement about what’s been accomplished so far. - Then, **emphasize the goals** of the small group discussion. |
| Interrupt each other while speaking   - This could be a good sign of engagement in the discussion, but it may also require some direction from you | - If someone is interrupted, offer them the opportunity to finish what he/she was saying. - It may also be an appropriate time to reaffirm some of the ground rules for discussion.   - **You can say:** “I want to make sure that I can hear everyone’s ideas and opinions. It would be really helpful if we could have 1 person talk at a time. If more than one person wants to talk at the same time, I’ll ask you to raise hands and I’ll number you off. That way, you’ll know when your turn is coming.” |
| Are being repetitious   - People repeat themselves because they don’t feel heard. | - Use **paraphrasing** to help that person summarize his or her thinking. **In your own words, say what you think the speaker said.**    - Occasionally preface with a comment like: “It sounds like you’re saying…did I get it?” |
| Make vague, awkward, or incomplete statements | - Help the speaker **relax** by **drawing him/her out with open-ended questions:**   - **You can say**: “Can you say more about that?” *or* “What do you mean by…?” *or* “What matters to you about that?” |
| Hesitate to contribute | - Don’t quickly press the issue, but instead look for an opportunity to **direct a question to the whole group**:   - **You can say:** “Are there other ways of looking at this?”   - Or, “Does anyone have a different point of view?” - There’s also opportunity to **direct a question to this person**:   - **You can say:** “What do you think of that approach/idea?” *or* “Do you have anything to add?” |
| Go off on a tangent | - Ask the person to help everyone see **how his/her point connects with the broader context**. - **You can say:** “How does your idea link up with [our topic]?”   - Validate the explanation: “Are you saying…[paraphrase]?”   - Then, follow-up with, “Okay, we have [name’s] idea. Whose turn is it to go next?” - You can also redirect by clarifying the objectives for the discussion. |
| Seem to have exhausted their contributions | - If you think the group has generated lots of good discussion already, then it’s fine to end. - If there are open questions or something needs clarity, you can use that to ask open-ended questions to elaborate more on an idea. - If you see a “gap” in ideas, ask the group for their opinion on that gap (e.g., “We haven’t talked about what doctors can do…”) |
| Come to the end of a part of the discussion | - Summarize ideas/decisions. |
| Are silent | - Tolerate silence if they seem to be gathering thoughts (look for non-verbal indicators of readiness to speak).   - You can count to 5 or 10 slowly before prompting for input. Especially at the beginning of the day, silence may be longer and more likely as people gather their thoughts and hesitate to be the first to speak up. - If simply quiet on subject, ask probing questions:   - **You can say:** “I’d like to get opinions from those who haven’t talked for a while.”   - Or, “Any new thoughts? [participant], you look as if you might be about to say something…” - You can also ask someone to restate the objective of the discussion. |
| Exhibit guarded expressions | - Make inquiry and probe. - **You can say:** “Looks like you’re having a reaction to that. I’m guessing you’re [emotion]. Am I close?” |
| Emotions are running “high” | - Acknowledge the emotion - **If it’s on task, you can ask probing questions:** - **You can say**: “You sound a bit worried. Can you say more about what you are thinking?”   - Then paraphrase the content of the thought and redirect to task. |
| Emotions are running “low” | - Invite expression of emotion with opinions. |
| Dispute/oppose/attack with loaded questions | - Turn loaded questions into problems to be addressed by group. |

**DVR Assignments**

**Instructions**

How to turn on DVR

Turn DVR over.

Slide the top bar from OFF to ON.

1. When ready to record, move the side bar to REC (a red light will flash on the top of the device to indicate it’s recording).
2. When you’ve finished recording, move the side bar to STOP (the red light will turn off).

# How to turn off DVR

1. Make sure the side bar is set to STOP.
2. Move top bar from ON to OFF.

# Note: Please be sure to STOP recording and turn OFF recorder. Each time you turn ON recorder a new audio file is created.

# Sub-Group Introductions and Ice-breaker

Time**:** 9:15-9:35am (20 minutes)

## Reminders to Facilitators

- Please make sure the recorders are working properly.
- Say your first name every time you start the recorder.
- Remind participants to say their first name whenever they speak; if they forget to say it, you should say it.

## Facilitate Activity

- *This morning, I would like to take a few minutes to get to know one another. My name is [_______], and my role today is to be your group facilitator. My intention is to support a free-flow of ideas, and help keep the group on schedule. I might have to cut the conversation short sometimes, but I want to give everyone opportunities to speak when they want to.*
- *As a reminder, we will be audio recording throughout today’s discussion, so* ***please begin your comments by using your first name. ‘This is John…****’.*
  - *We are doing this so we can* ***keep track of who is saying what*** *throughout the discussion, otherwise, peoples’ voices can sound like one another on the recording, and this makes it difficult for our transcriptionist and study to accurately keep track of everyone’s individual opinions.*
  - *To* ***protect your privacy****, it is important that you use only your first name. Our* ***recordings will be kept confidential, the transcripts will be de-identified****, and we will not use your name in anything we write about this session.*
  - *I will also try to use your name when speaking with you directly, so please do not feel singled out if I say your first name often during the discussion. Your specific comments will never be released in relation to your name.*
- As mentioned, **the ground rules for today’s conversations are:**
  - *Keep focused on issue/task.*
  - *Be respectful of everyone’s opinions, even if there are strong disagreements.*
  - *Everyone should have a voice in the discussions- if more than one person wants to talk at the same time, I’ll ask you to raise hands and I’ll number you off. That way, you’ll know when your turn is coming.*
  - *New and creative ideas are welcome. There’s no such thing as a bad idea!*
  - *Most importantly, we want you to* ***have fun****! Today is about getting your creative juices flowing and coming up with all types of ideas!*
- First let’s go around the table and introduce ourselves; please say….
- *Your name*
- *In what branch of the military your served.*
- Next, ask participants to pair off for the **“to the person sitting next to you**” activity
  - *Turn to the person next to you and find something that you have in common with that person. It can be a similar interest, military, family, education, etc. For example, “our grandmothers both went to the same schoolhouse in Milford, AK.” Also, see if you can go deeper than a common interest!*
  - *Now that you’ve gotten to know your partner a bit, let’s take a few minutes to share what you learned about each other.*

# Presentation #1: Provider

In this grounding activity, a clinician will provide insight into when scaling back is the right thing to do, and begin to understand how doctors think about scaling back and its challenges.

## Time: 9:35am-9:45am (10 minutes)

## Material

- PowerPoint Presentation

## Facilitate Activity

- No instruction.

# Presentation #2: Patient

In this grounding activity, we will begin to understand scaling back from the patient’s perspective.

## Time: 9:45am-9:55am (10 minutes)

## Material

- PowerPoint presentation

## Facilitate Activity

## No instruction.

# Q&A Session and Stretch Break

In this reflection activity, participants can ask questions to deepen their understanding of the information from the presentations, including scaling back in general.

## Time: 9:55am-10:05am (10 minutes)

## Material

- None

## Facilitate Activity

- No instruction.

# Transition to Activities

## Time: Start at 10:05am (about 1 minute)

## Material

- Big screen clock: (<http://ipadstopwatch.com/clock-fullscreen.html>)

## Facilitate Activity

- No instruction

# Case Review

In this framing exercise, we will present and review the patient and provider cases within sub-groups.

## Time: 10:05-10:15am (10 minutes)

## Materials

- Patient case
- Provider case
- Notepads
- Pens

## Reminders Facilitators

- CRC/CAS Facilitators: Highlight that the cases are about screening/tests in general.

## Facilitate Activity

*We’re going to start by sharing a story about scaling back [topic area]. This is just one example of a scaling back story. The story focuses on an upcoming medical appointment, and is told from the point of view of a patient and their provider. The story will be the basis for many of our other activities today.*

1. You can narrate the background description of the patient and provider stories.
2. Ask for a patient volunteer to act as the patient and provider and read their specific stories. It’s okay if the same patient reads both stories.
   - *Does anyone want to volunteer to be [patient]?*
3. After the group has read through the story, ask participants to take 1-2 minutes to reflect on what they heard from the story.
   - Ask them to think about (and write on their notepads, if they want to): **what they heard, what was surprising, and/or what was most interesting.**
4. Ask if anyone has questions about the story before moving on to the next activity.

# Mind-Mapping

In this framing/reflection exercise, sub-group members will visualize and summarize the facts of their case.

## Time: 10:15am-10:30am (15 minutes)

## Material

- Flipcharts
- Markers
- Notepads or post-it notes
- Recorder

## Reminder to Facilitators

- You may need to rely on encouraging techniques especially at the beginning.
- You can give positive feedback throughout but it needs to be equal. The acknowledgement helps people feel like they can and want to contribute. Don’t overuse “praise,” rather, use active listening skills.
- It’s okay if you’re not able to write down every idea- just try to get as many as you can!
- All ideas are good ideas- treat silly ideas the same as serious ideas.

## Facilitate Activity

*Now we’re going into a mind mapping activity where we’re going to visualize some of the main points of what happened in the story. I will try to capture as many thoughts and ideas as possible. Don’t feel like you have to soak it all in right now. Can someone begin to call out things they heard related to [main topic]?*

1. In large handwriting, write **“The Story”** on the flipchart paper, and ask participants for ideas from the stories they just read.
   - As people call out ideas, draw branches from the center and label each branch with **one key word**.
2. Try to write all thoughts and ideas as they come out.
   - You can encourage participants to record their thoughts on the paper in front of them and then calling out their ideas when they feel comfortable doing so.
   - Participants might draw from their own experiences for ideas- it’s okay to add those ideas to the mind map as well.
3. During the discussion, try to encourage participants to touch on the following points:
   - What we want to hear about **providers**:
     - *Does [doctor] think scaling back is the right thing?*
     - *Is scaling back easy or hard to do?*
     - *What considerations might the doctor have?*
   - What we want to hear about **patients**:
     - *What does [patient] think about scaling back?*
     - *Would scaling back be easy or hard to do?*
     - *What considerations might [the patient] have?*
4. Towards the end of the activity, you can start to group similar ideas together on the paper using a different colored marker.
5. Ask the group if they see any common themes.
6. Place the completed mind-map on the wall or window for the remainder of the forum.
7. Move onto group reflection questions.

**Ask participants questions to guide the discussion such as:**

- *What do you mean by…?*
- *Can you say more about that?*
- *Who else has an idea?*
- *What do others think?*
- *Let’s hear from someone who hasn’t spoken in a while.*
- *What are some other ideas or words that come to mind when you think of ________?* (an idea that doesn’t have many branches)
- *How do you see _____ connecting with ________? Can you help us make the connection?* (this is especially helpful when people go off on tangents).


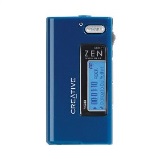


**Start Recorder**

## Group Reflection Questions (5 minutes)

- *Let’s take a look at what we’ve created…what jumps out at you about the story? What’s surprising?*
- *You have done some solid thinking about [topic]. Anything else that’s still on our minds that’s not up there? What’s missing from the story?*
- *Do we have a better sense of the complexity of the issue?*
- *We’ll continue to have this conversation, but in a new context. The next activity where we’re going to understand the landscape and understand the interactions that take place.*

**Stop Recorder**


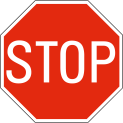


# Business Origami

In this framing/reflection exercise, sub-group members imagine the patient and provider in the story are meeting for an appointment. Participants work to gain a deeper understanding of the nature of interactions and the conversations, by creating a visual landscape.

Time: 10:30am-10:50am (20 min)

## Material

- People, place, things icons
- Large paper (ideally 2 sheets/table)
- Markers
- Painters Tape (specific to your group’s colors)
- Recorder

## Reminder to Facilitators

- Some participants may want to stand back and not engage, but try to encourage participation by all. Prompt quiet members for their thoughts.

## Facilitate Activity

*Let’s imagine that [patient] and [provider] are meeting for an appointment. Think about what [patient]’s journey and [provider]’s journey is like to the appointment, during the appointment, and after the appointment. We’re going to map out this interaction/conversation.*

1. Begin by bringing out icons, paper, and markers.
2. Facilitator places the patient and doctor icons in the middle of the paper.
3. Ask participants to select other icons that represent the people, place, or things that represent ideas that arose in mind mapping.
4. Ask participants to place the icons in relation to one another in a way that represents the situation from the story.
5. Ask participants to get as many ideas as they can in **10 minutes**.
   - This activity **can be prompted by the case** but it’s okay if participants want to bring in elements of their own experience.
   - Emphasize that no idea is a wrong idea –they may spark great out-of-the-box ideas.
6. As participants come up with ideas, hand out markers and have them **draw directional arrows** to show connections between the icons – with or without labels.
7. Tape the icons on the paper towards the end (you can also ask for volunteers to help).
8. Hang the completed map on the wall or window for the remainder of the forum.
9. Lastly, move onto the group reflection questions.

**Ask participants questions to guide the discussion such as:**

- *What are some ideas you have about what all is occurring before, during, and after the visit?*
- *Think about [patient] going to see [provider] in the clinic… what are the steps involved?*
  - *Who’s involved?*
  - *What do you think the interaction is like?*
- *You had a good point about [idea]… what do you mean by that?*


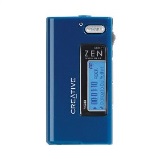


**Start Recorder**

## Group Reflection Questions (5 minutes)

- *Let’s take a look at what we’ve created…*
- *Look at all of the things that influences the patient and doctor…*
- *What jumps out at you right away? What matters to you about that?*
- *What is most surprising? Why? (gets at rationale for placement/connections)*
- *What do you see as the most important/key things in what’s been created? Why?*
- *Was there anything new that you didn’t know or hadn’t considered before?*
- *Anything else that’s still on our minds that’s not here? What’s missing from the story?*

**Stop Recorder**


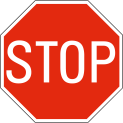


# Empathy Maps

In this framing/reflection exercise, sub-groups will take a deeper dive into what’s going on in the minds of the patient and doctor (thinking/feeling, hearing, seeing, saying/doing) in the case about scaling back.

Time: 11:00am-11:40am (40 minutes)

## Material

- Flipcharts
- Blank copies of empathy map canvas (should include: name, age, gender, and occupation)
- Markers
- Post-it notes
- Tape
- Recorder

## Reminders to Facilitators

- Make sure to keep participants **on topic** and thinking about the story.
- Occasionally **ask participants to explain an idea.**
  - **Ex:** What did s/he mean by it? Clarification or more detailed explanation of post-it? Why did s/he think it fit in a certain category?
- If one section is lacking ideas, prompt group members to think about that particular area.
- Go with the flow!

## Facilitate Activity

**Patient Map** (20 minutes)

*In this next activity our goal is to try to understand what’s going on with [patient] and [provider] during their interaction. Let’s try to think through what’s going on in their heads… what they’re thinking and feeling.*

1. Place the blank empathy map on the flipchart.
2. **Start with the “seeing” quadrant,** and move around clockwise.
   - Or, you can start wherever someone already has something to add- order is not important it just helps get things going.
   - *Let’s start with [patient]. What is he/she: 1) seeing, 2) saying/doing, 3) hearing, 4) thinking and feeling?*
   - *Why do you think s/he’s seeing ____?*
   - *While all of this is happening, what is the reaction? What is s/he saying/doing?*
3. Ask participants to write ideas on post-it notes and hand them to you to place on the empathy map. Participants should say which section it goes into.
   - You can also write on the post-it notes for some participants, and let them know, *I can write this down for you*.
4. You can also invite participants to come up and place post-it notes on the board.
   - *Can you help me try to get some of that down?*
5. Move to the pains and gains sections. Write pains and gains directly on the empathy map.
   - *Now that we know what [patient] is seeing, saying, hearing, and thinking, we also want to think about what are [patient]’s biggest problems or ‘pains’ during this moment of that conversation/interaction.*
   - *Let’s take a moment to figure out what is the biggest problem.*
   - *Now, let’s think about the opposite… what is [patient] getting out of the conversation? What is s/ he gaining?*
     - Ideas from above can be duplicated here- ask participants to list the “key” things.
6. Patient wrap-up
   - *Now that we’ve understood what [patient] is feeling, let’s explore the provider perspective…what [provider] is feeling.*

**Provider Map** (15 minutes)

1. Repeat the above process, but from the provider’s point of view.
2. When finished- *While you’re at lunch, if you want to come back and read through the diagram that’s okay. When we come back together we’ll reflect on the activity a bit.*
3. Move onto the group reflection questions **before lunch**.


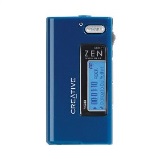


**Start Recorder**

**Stop Recorder**


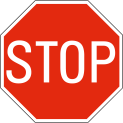


## Group Reflection Questions (5 minutes)

- *Let’s take a look at what we’ve created…* or *look at all of the things that influences doctor and patient…what jumps out at you?*
- *What are the most important things in what’s been created? Why?*
- *What is most surprising? Why was that surprising?*
- *What was it like to begin to think through each interaction?*
- *Do you see similarities between how [patient] is thinking and how [provider] is thinking?*
- *What are the big differences in what [patient] is thinking compared to what [provider] is thinking?*
- *Anything else that’s still on our minds that’s not here? What’s missing from the story?*
- *Is there anything you learned that you didn’t know before?*

# ‘Identifying Solutions’ Card Game

In this ideation exercise, participants will work in pairs to identify solutions (or “scaling back strategies”) to specific scenarios doctors can use when the best care is to scale back a medical service.

Time: 12:20pm-1:50pm (90 minutes)

## Material

- Envelopes with 5 cards for 1 scaling back scenario
  - Each pair will have a stack of 8 worksheets (we’re only expecting each pair to complete 5-6 worksheets- the other 3 in the pile are in case participants get stuck and want to select a different scenario)
- Blank scenario worksheets
- Cases for demonstration
- Post-it notes
- Pens/Markers
- Sand timers
- Tape (2 per table)

## Reminders to Facilitators

- This activity will **NOT** be audio recorded.
- For ALL CRC scenarios, assume that patient has no history of polyps, no family history, and no prior colon cancer diagnosis.
- For ALL CAS scenarios, assume that patients have no symptoms and no history of stroke.
- Dealing with an uncreative pair: Ask our roving facilitators for ideas on how to get people “unstuck.” Get them started on something that might seem silly or obvious!

## Facilitate Activity

**Example Instruction/Walk-through** (10-15 minutes)

*Debrief key take-aways from empathy mapping to transition to the identifying solutions activity. This activity is about brainstorming and coming up with as many ideas as possible. We are going to spend time coming up with creative solutions to some of the challenges (either patient or provider) to scaling back.*

*Let’s take a closer look at how to do this by walking through an example together.*

*The sheet in front of you is what we call a ‘scenario worksheet.’ The scenario we’re looking at right now is based on the [condition] case we read out loud earlier.*

- - *Each box represents a different category. At the top, in boxes 1 and 2, we’re given basic information about the patient and provider in the scenario.*
  - *The third box is a want/need, so in this case, [read the want/need].*
  - *The fourth box is a motivation (or because), so, [read the motivation/because].*
  - *The fifth box is a barrier, or something that gets in the way of the want/need [can re-read want/need] from happening.*
  - *Taken together, the boxes make up a scenario. For example, ‘Now that we know that [name from scenario] wants/needs____, because ____, how do we overcome the fact that [barrier]? So, a solution might be, _____.’*

**Identifying Solutions Pair Review**

*We are going to work in pairs for this activity. Please pair up with the person next to you.* *If there is an odd number in the group, create a group of 3.*

1. Ask participants to pick an envelope from the stack on the table.
2. Ask participants to tape each card to the matching boxes on the scenario worksheet.
3. Then, ask participants to read the full scenario together out loud.
4. As they’re reading, instruct participants to circle things they think might keep the doctor and patient from scaling back or stopping.
5. Next, ask participants to flip their timers.
6. Ask each person to write as many solutions as they can on their post-it notes and stick them anywhere on the worksheet until the 5-minute timer runs out (we’re hoping that people will be able to come up with 6-8 solutions/worksheet).

**Choosing the “best” solutions**

*This activity is about choosing which 1-3 scaling back strategies/solutions are most important to you. You will be voting on your own ideas for each worksheet. I will give each person 3 dot stickers and markers for this activity.*

1. Ask each person to place dot stickers (or use a colored marker if they prefer) on the best solutions they created with their partner (they can put more than one dot on a solution).
2. Ask pairs to review the solutions that have dots. They can choose the best solution based on the number of dots and/or through discussion with their partner.
   - It’s okay if they can’t decide on only 1 solution, but ask them to try to keep it between 1-3 solutions.
3. Based on their best solution, ask participants to write down the big problem it solves and the solution in one of the boxes on the worksheet.
4. Ask participants to repeat Step 9 if they have more than 1 “best” solution.
5. Ask participants to tape their extra stickies and other notes to the back of the worksheet.
6. Participants repeat these steps until they have finished all the envelopes or have run out of time.

## Notes

*How many scenario worksheets were participants able to complete?*

*Pair 1: _____ Pair 2: ______ Pair 3: ______ Pair 4: ______ Pair 5: ______*

# Prioritization

In this ideation/synthesis exercise, sub-group members will select the strategies that are most important from all strategies identified in their group.

## Time: 1:50pm-2:45pm (55 minutes)

## Material

- Dot stickers and markers
- Completed worksheets from identifying solutions activity
- Recorder

## Facilitate Activity

**Group Voting**

*This is where we will dot-vote as a group across ALL worksheets generated by ALL pairs.*

## Facilitators hang all worksheets (with the 1-3 prioritized solutions) on the wall.

- - You can group the worksheets by scenario since some will be the same.

1. Facilitators read the scenarios and pairs presents their solutions for each of the 5 worksheets.
2. Give each person 6 dots and markers.
3. Ask participants to place 1 dot on each of their 6 favorite solution statements.
   - Participants can only place 1 dot on each solution statement.
4. Facilitators select the top 4-6 worksheets (based on dot voting) and groups them on the wall.
5. Move onto the group reflection questions.


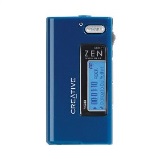


**Start Recorder**

**Stop Recorder**


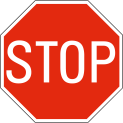


## Group Reflection Questions (10 minutes)

- *Why are these worksheets top priority?*
- *Are there common themes that tie them together? How are they different?*
- *Are we covering everything?*
- *For solutions that were not prioritized:*
  - *Are you okay with leaving these solutions behind (i.e., they’re not going into Phase 2)?*
  - *There are not as many dots on these solutions. Why didn’t these make it into the top group?*
- *What was challenging about this activity?*

# Thank You

Provide concluding thoughts on scaling back, thank participants, and remind them of Phase 2.

## Time: 2:45-3:00pm (15 minutes)

## Material

- Power point presentation

## Facilitate Activity

- Take notes if there is additional discussion

# Notes:

# ____________________________________________________________________________________________________________________________________________________________________________________________________________________________________________________________________________________________________________________________________________________________________________________________________________

# Session Wrap-Up and Post-Survey

## Time: 3:00-3:30pm (30 minutes)

## Material

- Post-forum surveys
- Gift cards
- Copies of consent forms

## Facilitate Activity

- Thank participants for their time and contributions today.
- Ask participants to complete their survey.
  - Inform participants that their survey responses will not be shared or discussed during the forum and all responses will be kept confidential.
- Distribute gift cards and copies of consent forms.

## YOU’RE DONE!!

THAT WAS AWESOME!

GREAT JOB!

WAY TO GO!

**
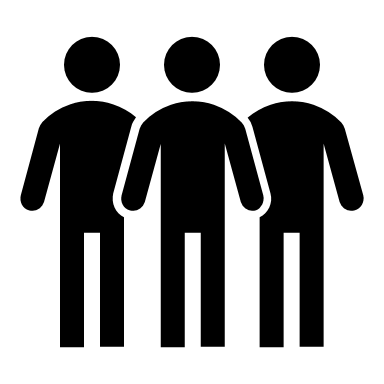

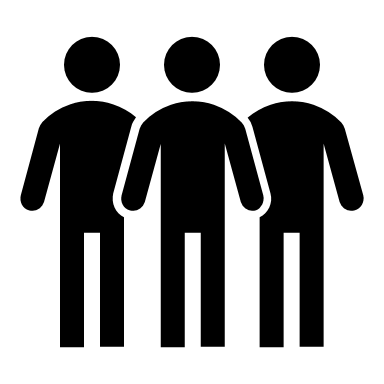

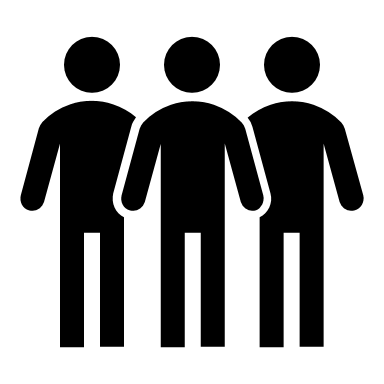
**

**NCRC/VAAHS Contacts**

| **Department** | **Phone Number** |
| --- | --- |
| NCRC Security Post  Director of Security | (734) 764-9011  (734) 647-1079 |
| VA Patient Advocate | (734) 845-5473/ (734) 845-3402 |
| VA Social Work Services | (734) 845-3417 |
| VA Crisis Line | (800) 273-8255 and Press 1 |
