# Supplementary material for: Developing Strategies to Reduce Unnecessary Services in Primary Care: Protocol for User-Centered Design Charrettes
Source: JMIR Res Protoc. 2019 Nov 26;8(11):e15618. doi: 10.2196/15618 (PMC6904896; doi:10.2196/15618)
Supplement: Multimedia Appendix 3 [file resprot_v8i11e15618_app3.docx]

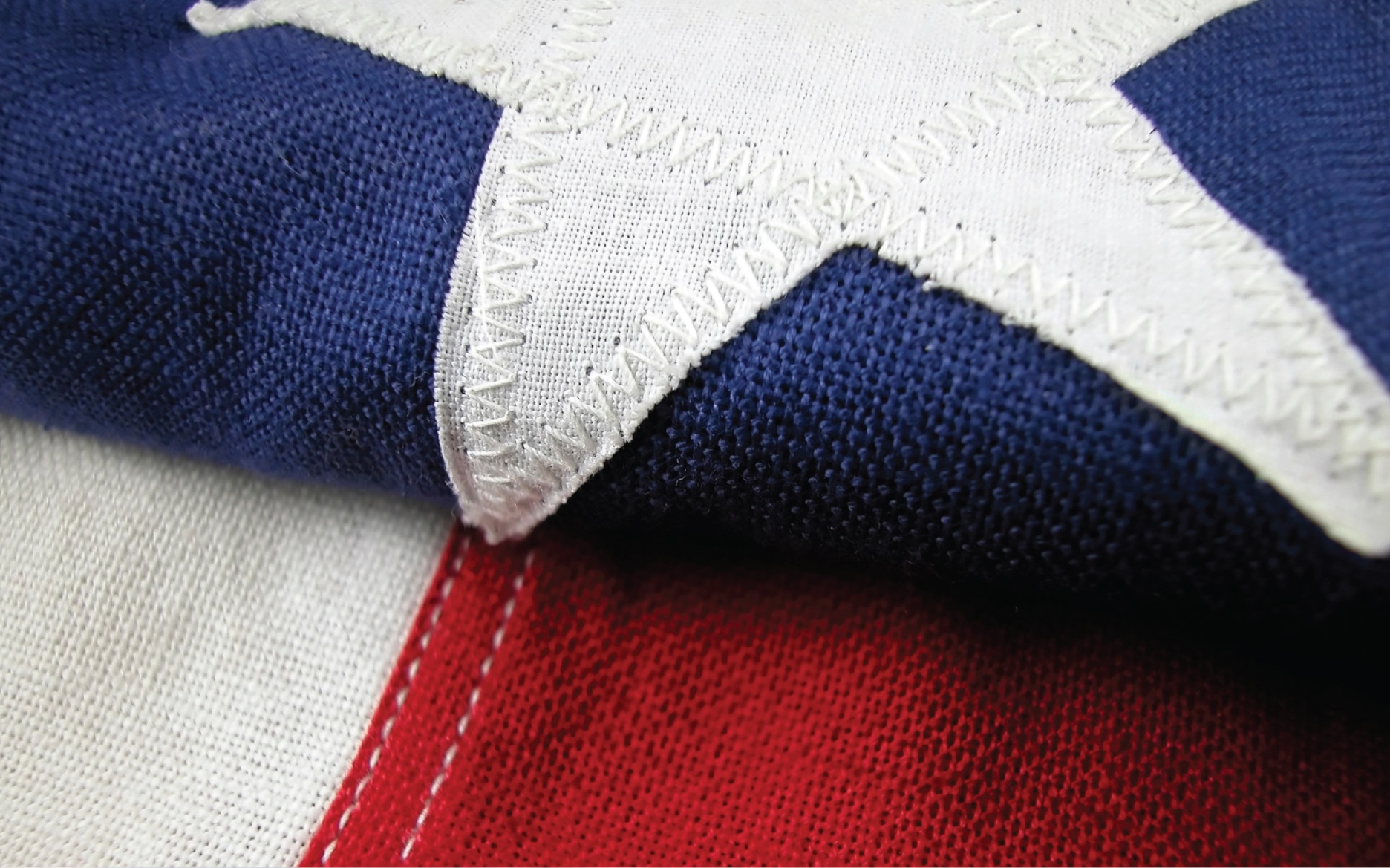

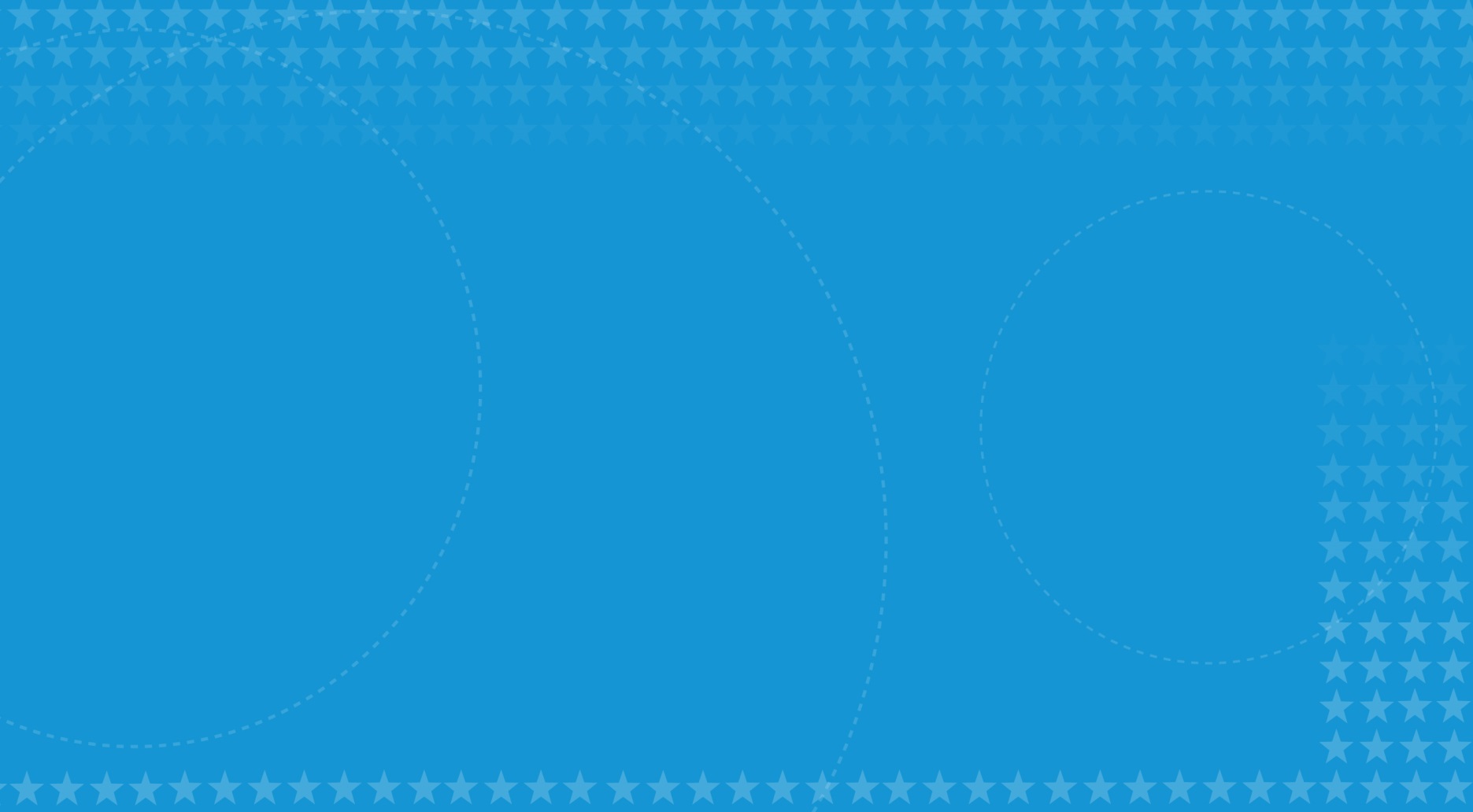


**Patient Collaborative Design Forum**

**Pre-Forum Survey**

**Participant ID Number: _________**

| **Military Service and Other Demographics** |
| --- |

**Q1: What is your military branch of service? (check all that apply)**

□ U.S. Army (active component)

□ U.S. Navy (active component)

□ U.S. Air Force (active component)

□ U.S. Marine Corps (active component)

□ U.S. Coast Guard (active component)

□ U.S. National Guard

□ U.S. Reserves

**Q2: Were you ever deployed to a combat zone?**

□ Yes

□ No

**Q3: In what military era did you serve? (check all that apply)**

□ World War II

□ Korean War

□ Vietnam War

□ Gulf War (Operation Desert Shield/Storm)

□ War on Terror (Iraq, Afghanistan, Islamic State; OEF/OFS/OIF/OND/OIR)

□ Peacetime service ONLY (i.e., none of the above)

□ Other (please list): _____________________________

**Q4: What is your current rank or your rank at discharge?**

□ E1-E4

□ E5-E6

□ E7-E9

□ WO1-WO5

□ O1-O3

□ O4-O10

**Q5: Please indicate your year of birth below:**

**___________ (example: 1950)**

**Q6: What is your gender?**

□ Male

□ Female

□ Other/Transgender

**Q7: Are you Hispanic or Latino?** (Defined as a person of Cuban, Mexican, Puerto Rican, South or Central American, or other Spanish culture or origin, regardless of race)

□ No

□ Yes

**Q8: What is your race?**

□ American Indian or Alaska Native

□ Asian

□ Black or African American

□ Native Hawaiian or Other Pacific Islander

□ White

□ Other (Please list) ___________________________

**Q9: What is the highest degree or level of education you have completed?**

□ Less than high school

□ High school graduate (diploma or GED)

□ Some college/trade school

□ Associate’s degree

□ Bachelor’s degree

□ Master’s degree or more

**Q10: What is your marital status?**

□ Single (never married)

□ Married

□ Separated

□ Widowed

□ Divorced

| **General Health Status** |
| --- |

**Q11: In general, would you say your health is:**

□ Excellent

□ Very good

□ Good

□ Fair

□ Poor

| **Satisfaction with Health Care** |
| --- |

| **Q12: All things considered, on a scale from 1-10, how satisfied are you with your health care in the VA?** | | | | | | | | | |
| --- | --- | --- | --- | --- | --- | --- | --- | --- | --- |
| **1** | **2** | **3** | **4** | **5** | **6** | **7** | **8** | **9** | **10** |
| **Completely**  **Dissatisfied** | |  |  |  |  |  |  | **Completely Satisfied** | |

| **General preferences for seeking healthcare** |
| --- |

**Q13: Please rate how much you personally disagree or agree with each statement below:**

|  | **Strongly**  **Disagree** | **Somewhat Disagree** | **Neither Agree nor Disagree** | **Somewhat Agree** | **Strongly Agree** |
| --- | --- | --- | --- | --- | --- |
| A. It is important to treat disease even when it does not make a difference in survival |  |  |  |  |  |
| B. It is important to treat a disease even when it does not make a difference in quality of life |  |  |  |  |  |
| C. Doing everything to fight illness is always the right choice |  |  |  |  |  |
| D. When it comes to health care, the only responsible thing to do is to actively seek medical care |  |  |  |  |  |
|  | **Strongly**  **Disagree** | **Somewhat Disagree** | **Neither Agree nor Disagree** | **Somewhat Agree** | **Strongly Agree** |
| E. If I have a health issue, my preference is to wait and see if the problem gets better on its own before going to the doctor |  |  |  |  |  |
| F. If I feel unhealthy, the first thing that I do is to go to the doctor and get a prescription |  |  |  |  |  |
| G. I often suggest that friends and family see their doctor |  |  |  |  |  |
| H. When it comes to health care, watching and waiting is never an acceptable option |  |  |  |  |  |
| I. If I have a medical problem, my preference is to go straight to a doctor and ask his or her opinion |  |  |  |  |  |
| J. When it comes to medical treatment, more is usually better |  |  |  |  |  |

| **Trust** |
| --- |

**Q14: Please indicate how much you disagree or agree with the following statements:**

|  | **Strongly**  **Disagree** | **Somewhat Disagree** | **Neither Agree nor Disagree** | **Somewhat Agree** | **Strongly Agree** |
| --- | --- | --- | --- | --- | --- |
| A. I have complete trust in my doctor to provide the medical care I need. |  |  |  |  |  |
| B. I have complete trust in my local VA Healthcare System to provide the medical care Veterans need. |  |  |  |  |  |
| C. I have complete trust in the national VA Healthcare System to provide the medical care Veterans need. |  |  |  |  |  |

| **Patient-provider Relationship** |
| --- |

| **Q15: How would you describe your relationship with your current VA primary care clinician?** | | | | | | | | | |
| --- | --- | --- | --- | --- | --- | --- | --- | --- | --- |
| **1** | **2** | **3** | **4** | **5** | **6** | **7** | **8** | **9** | **10** |
| **New Relationship** | |  |  |  |  |  |  | **Established, Comfortable Relationship** | |

**Q16: How long have you been seeing your current VA primary care clinician?**

□ Less than 6 months

□ 6 months to less than 1 year

□ 1 year to less than 5 years

□ 5 years or more

**Q17: To what extent do you rely on the VA to provide for your health care needs?**

□ All of my health care needs

□ Most of my health care needs

□ Some of my health care needs

□ Almost none of my health care needs

**Q18: How many minutes do you typically spend meeting in person with your VA primary care provider during a clinic visit?**

____ minutes

**Q19: Please indicate below how much control you like to have when decisions are being made about your medical treatment**

□ I prefer to make the decision about which treatment I receive

□ I prefer to make the final decision about my treatment after seriously considering my doctor’s opinion

□ I prefer that my doctor and I share responsibility for deciding which treatment is best for me

□ I prefer that my doctor makes the final decision about which treatment will be used, but seriously considers my opinion

□ I prefer to leave all decisions regarding treatment to my doctor

**Q20: Please think about your current VA primary care provider when you answer the following four questions:**

|  | **All of the time** | **Most of the time** | **Some of the time** | **Rarely (or Seldom)** | **None of the time** |
| --- | --- | --- | --- | --- | --- |
| A. How often does your doctor offer you choices in your medical care? |  |  |  |  |  |
| B. How often does your doctor discuss the pros and cons of each choice with you? |  |  |  |  |  |
| C. How often does your doctor get you to state which choice or option you prefer? |  |  |  |  |  |
| D. How often does your doctor take your preferences into account when making treatment decisions? |  |  |  |  |  |

| **Opinions** |
| --- |

**Q21: If a doctor told you that something (i.e. medication, screening, or treatment) is no longer needed, how comfortable would you be:**

|  | **Very comfortable** | **Somewhat comfortable** | **Somewhat uncomfortable** | **Very uncomfortable** |
| --- | --- | --- | --- | --- |
| A. stopping or reducing something? |  |  |  |  |
| B. asking your doctor questions about why something is no longer needed? |  |  |  |  |
| C. disagreeing with your doctor’s opinion about stopping or reducing something? |  |  |  |  |

**Q22: If you came to your medical appointment with an opinion that was different than what was recommended (Example: you believe you should continue taking the same dose of a medication but your doctor recommends cutting back), how likely would you be to change your opinion?**

□ Very likely

□ Somewhat likely

□ Somewhat unlikely

□ Very unlikely

**Q23: How comfortable would you be receiving information about stopping or reducing something (i.e. medication, screening, or treatment) by the following methods:**

|  | **Very comfortable** | **Somewhat comfortable** | **Somewhat uncomfortable** | **Very uncomfortable** |
| --- | --- | --- | --- | --- |
| A. Face-to-face with your doctor |  |  |  |  |
| B. Face-to-face with a nurse |  |  |  |  |
| C. Face-to-face with a pharmacist |  |  |  |  |
| D. Mailed letter or pamphlet |  |  |  |  |
| E. E-mailed letter or pamphlet |  |  |  |  |

**Q24:** Sometimes, stopping or reducing medical treatments and tests is the right thing to do. (Examples include: medication doses that can be reduced; routine screening tests that are not necessary because they are not helpful; or duplicate a test that has already been done.)

It can be challenging for doctors and patients to know when it is better to stop or reduce treatments and tests.

**For each item below, rate how acceptable you think each idea might be if it were used to help stop or reduce certain treatments and tests:**

|  | **1** | **2** | **3** | | **4** | **5** |
| --- | --- | --- | --- | --- | --- | --- |
|  | Not at all | | | Very Much | | |
| A. Provide a summary to clinicians of their own prescribing habits, so they can review and adjust their practices |  |  |  | |  |  |
| B. Provide a summary to clinicians of their own prescribing habits compared with their peers |  |  |  | |  |  |
| C. Provide a reward (e.g. financial bonus, promotion) to clinicians for stopping or reducing when appropriate |  |  |  | |  |  |
| D. Provide a reward (e.g. financial bonus) to health systems or leadership for appropriate stopping or reducing within their system |  |  |  | |  |  |
| E. Report hospital performance on stopping and reducing to the public |  |  |  | |  |  |
| F. Have prescription or screening requests, initiated by a clinician, reviewed and approved by a pharmacist or specialist |  |  |  | |  |  |
| G. Hand out educational materials about stopping or reducing to patients |  |  |  | |  |  |
| H. Provide training to clinicians on how to talk with patients about stopping or reducing |  |  |  | |  |  |
| I. Provide clinicians with decision-making (about stopping or reducing) tools to help during a medical appointment |  |  |  | |  |  |
| J. Have national experts create guidelines for clinicians on how and when to stop or reduce |  |  |  | |  |  |
| K. Have the local hospital create guidelines for clinicians on how and when to stop or reduce |  |  |  | |  |  |
| L. Use social media (e.g. Facebook, Twitter) to provide information about the need to stop or reduce certain treatments |  |  |  | |  |  |
