# Supplementary material for: Developing Strategies to Reduce Unnecessary Services in Primary Care: Protocol for User-Centered Design Charrettes
Source: JMIR Res Protoc. 2019 Nov 26;8(11):e15618. doi: 10.2196/15618 (PMC6904896; doi:10.2196/15618)
Supplement: Multimedia Appendix 4 [file resprot_v8i11e15618_app4.docx]

| **Phase 1 UCD Activities** | | |
| --- | --- | --- |
| **Activity** | **Goals** | **Supports which part of the model?** |
| Presentations (by VA doctor and VA patient) | To help participants understand de-intensification from the patient’s perspective and the provider’s perspective | *[Grounding]* |
| Case Review about de-intensification | To highlight some of the reasons why de-intensification can be so challenging and to provide inspiration for the remaining UCD activities | *[General problem framing]* |
| Mind Mapping | To help participants visualize and summarize the key facts related to the de-intensification case review | *[General problem framing]* |
| Empathy Mapping | To allow participants to better understand the motivations and frustrations of a patient and a provider involved in scaling back | Empathize |
| Business Origami | To help participants map out what a medical appointment – where de-intensification is considered – would look like; to provide participants with a better understanding of all the people involved in de-intensification (e.g., pharmacists, caregivers, outside doctors), the environment in which de-intensification happens (e.g., busy clinic appointment), and the interaction between these | Empathize and Define |
| Identifying Strategies Card Game | To apply the learnings from the previous activities to develop high-level “de-intensification strategies” for primary care | Define and Ideate |
| Dot voting | To collaboratively prioritize the identified strategies | *[Prioritization]* |
|  |  |  |
| **Phase 2 Design Activities** | | |
| WhoDo | To define and refine the broad de-intensification strategies identified in Phase 1 by identifying strategies that include the specific stakeholders involved, their required actions, potential obstacles to the action, and approaches to overcoming the barriers | Ideate and Prototype |
