# Supplementary material for: Developing Strategies to Reduce Unnecessary Services in Primary Care: Protocol for User-Centered Design Charrettes
Source: JMIR Res Protoc. 2019 Nov 26;8(11):e15618. doi: 10.2196/15618 (PMC6904896; doi:10.2196/15618)
Supplement: Multimedia Appendix 5 [file resprot_v8i11e15618_app5.docx]

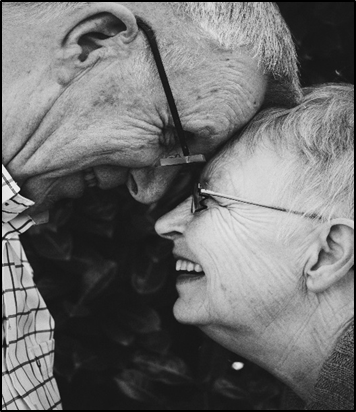
**Thinking about Scaling Back: Diabetes Medications**

John Foster is a 76-year-old retired welder. He was diagnosed with diabetes 16 years ago. The diabetes has caused him to have kidney troubles and pain in his feet. He checks his blood sugar several times a day. He takes multiple medications to help control his diabetes. For a long time now, his blood sugars have been normal with these medications. He has not had side effects, but he dislikes pricking his fingertips to check his blood sugar. He also takes several more medications since having a minor stroke and heart attack a while back. John’s wife works to make sure he never misses a medication.

John Foster and his wife Kathy

John recently talked to his friend Larry who also has diabetes. Larry mentioned that he was having side effects from his medications; his blood sugar was getting too low, causing him to get dizzy and have cold sweats. He spoke to his doctor and they stopped a medication. Since stopping, Larry said he has felt less tired, less anxious, and less irritable. Larry told John that many people start to have problems with low blood sugars as they get older.

**John’s Story:** John thinks about his next appointment with Dr. Forman and how a conversation with him about his diabetes medications might go.

***“If my doc thinks it would be ok, I’d be very grateful to be off it, quite frankly.”***

“It’d be good to stop a medication, but I’d have to talk about it with my doctor. I don’t know what would happen if I stopped taking a medication. What if my blood sugar goes back up? My doctor still refills all of the meds, so he must think I probably still need them.

But you do naturally talk to people about what’s going on with your health. Sometimes you hear or read conflicting comments about a certain medication…might cause you to want to give it up if possible.

But if my doctor says I still need the medication, then that’s enough. He knows better than me. If there’s time at the next visit, maybe I’ll bring it up. If my doc thinks it would be ok, I’d be very grateful to be off it, quite frankly. But there might not be time. I’ve got other health issues to discuss that are important. And I hate to take too much of his time and pester him with every little concern I have from the things my friends and I read.”


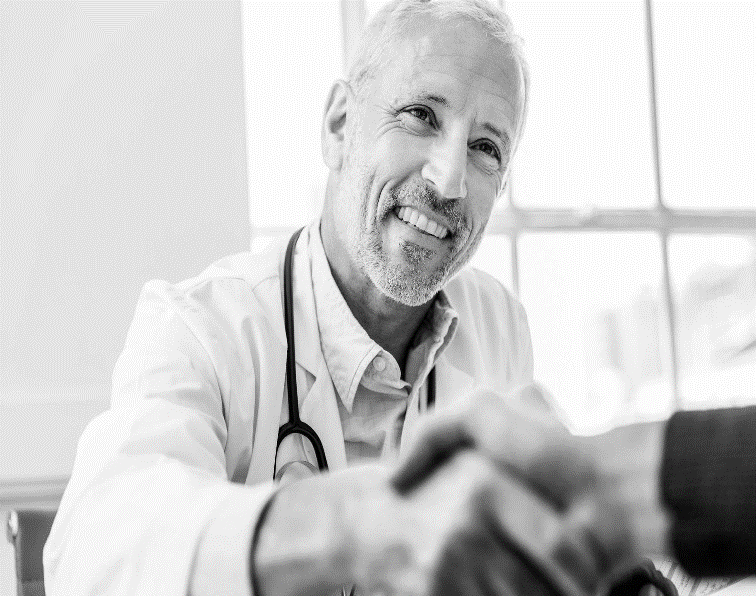
Dr. Forman is John’s primary care physician (PCP), and has been in practice at the VA for years. He has taken care of John for 10 years and sees John several times each year. Dr. Forman discusses scaling back treatment for his older patients with diabetes. He notes that scaling back can often be the best thing for a person. This is especially true when a patient’s blood sugar is lower than what people would consider to be safe and helpful (A1c below 6.5%). However, he shares that he can’t always fit these discussions into a busy visit.

Dr. Forman

**Dr. Forman’s Story:** Dr. Forman shares his own opinions of scaling back diabetes medications for his older patients with diabetes.

***“You can be too aggressive if you focus only on getting blood sugar lower.”***

“As a PCP you have to have a broad view of your patient. Diabetes is a good example. You can be too aggressive if you focus only on getting blood sugar lower. You have to take the whole person into account.

Being very aggressive controlling blood sugars doesn’t always make sense for older people. The benefit gets smaller and the side effects go up. So, I have to consider the whole person. It’s case-by-case whether scaling back is the best thing. But time is a big issue. There are so many considerations. For example, you have to consider how bad the diabetes was before they started that medication. And if somebody comes in about four other things, and you’ve got 10 minutes to see them…stopping a medicine probably becomes less of a priority.

Also, it can be difficult and time-consuming persuading older patients to withdraw after years of taking a medication. I’ve had patients that when I’ve said initially we wanted them to come off a medicine, they say, ‘oh no, I’ve been on that for ages, and I don’t want to come off it.’”
