# Supplementary material for: Developing Strategies to Reduce Unnecessary Services in Primary Care: Protocol for User-Centered Design Charrettes
Source: JMIR Res Protoc. 2019 Nov 26;8(11):e15618. doi: 10.2196/15618 (PMC6904896; doi:10.2196/15618)
Supplement: Multimedia Appendix 6 [file resprot_v8i11e15618_app6.docx]

Appendix C.

Mind Mapping Example


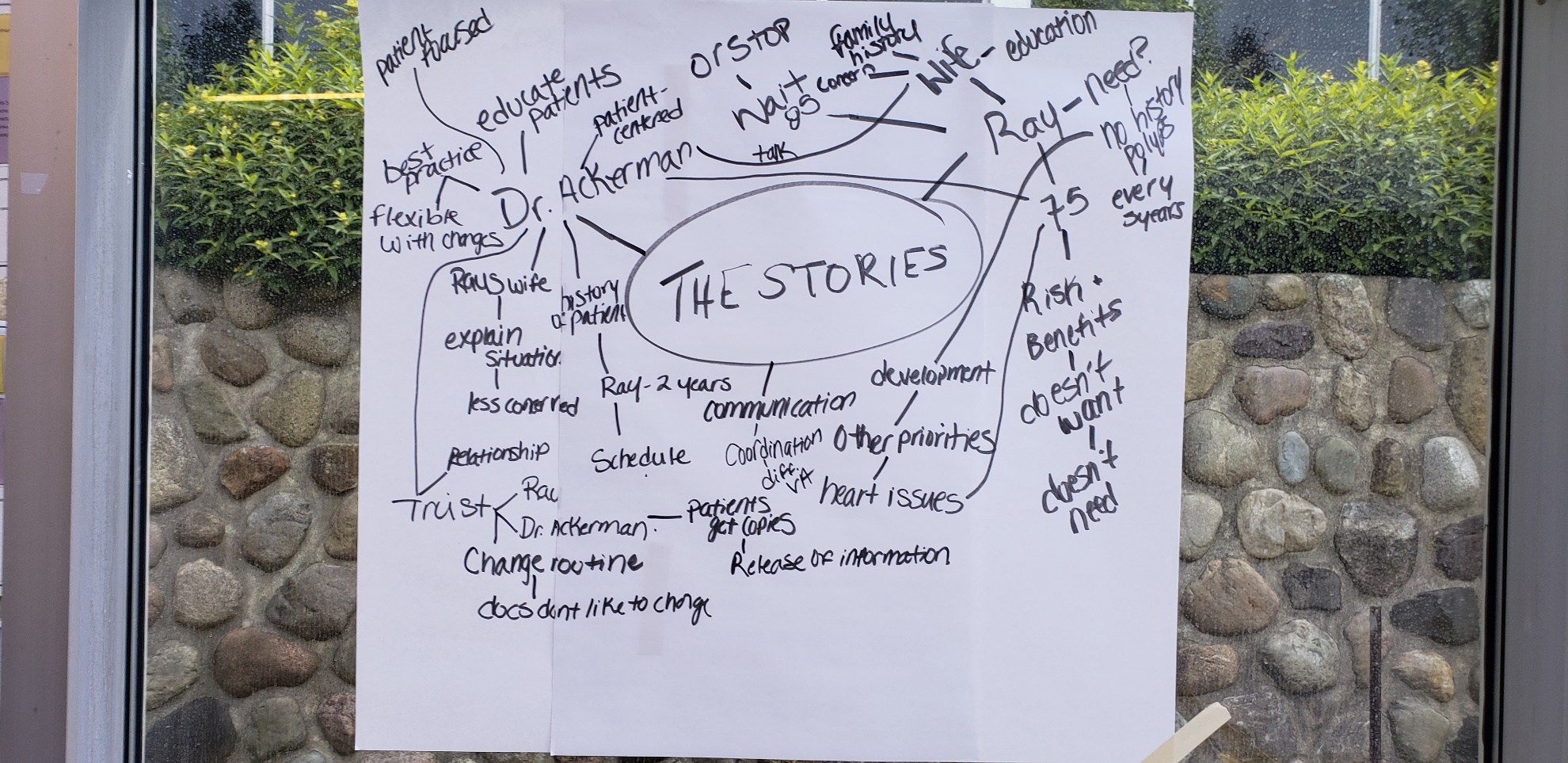


Business Origami Example


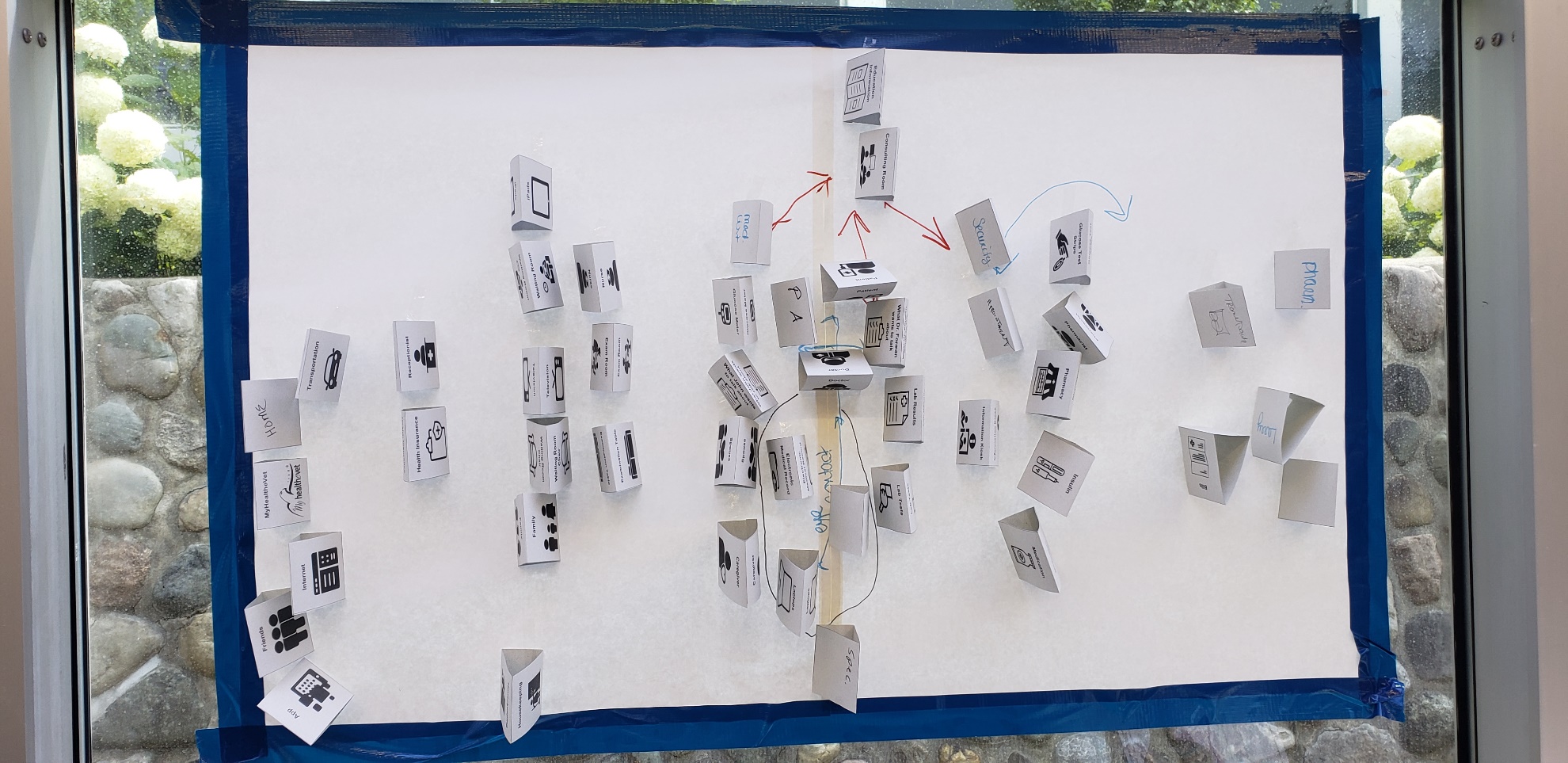


Empathy Mapping Examples (Provider, Patient)


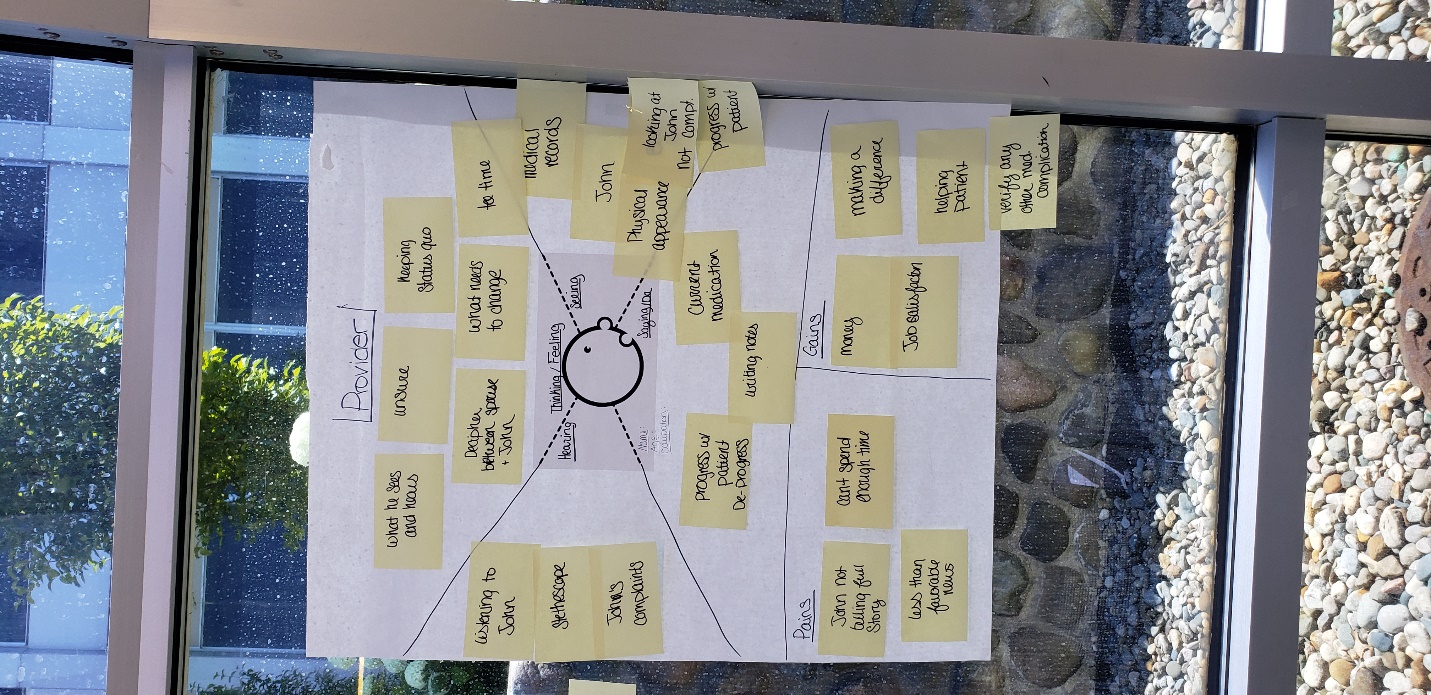

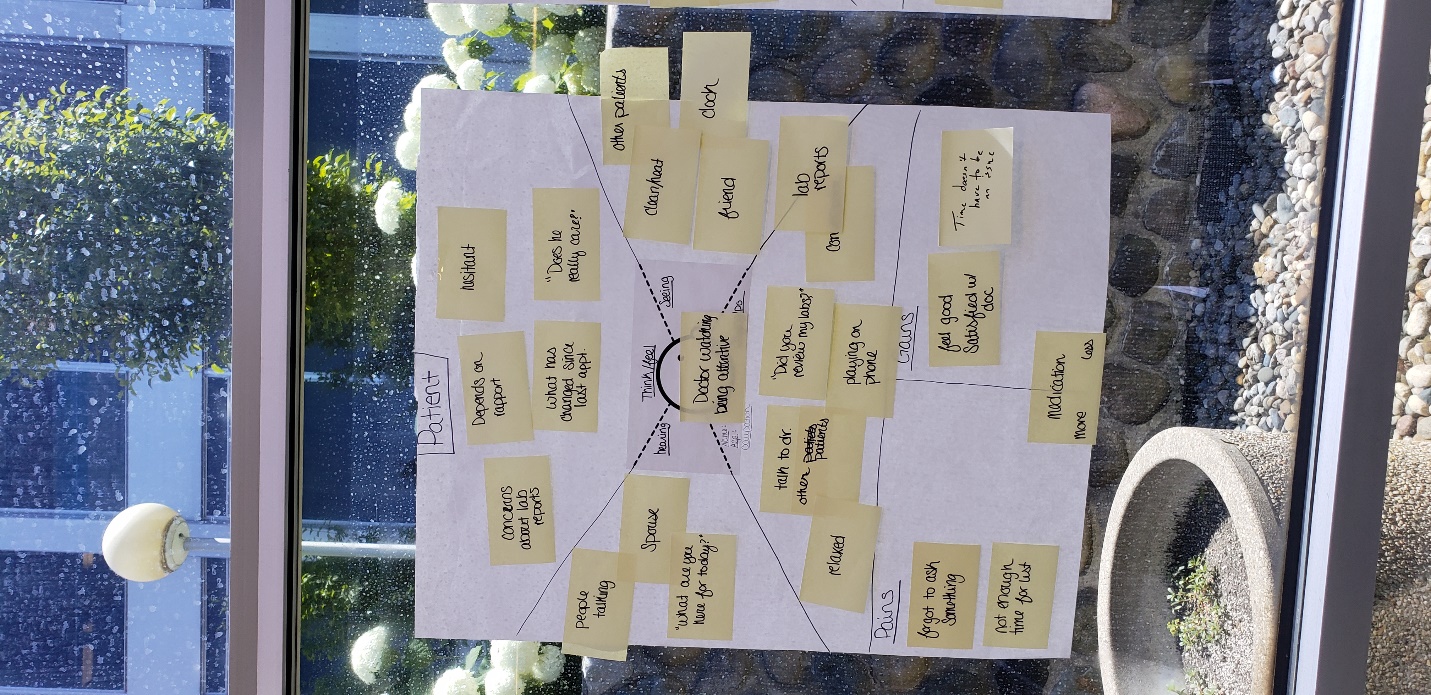


WhoDo matrix structure

| Healthcare Level | Who/Do | Barrier | How to Overcome |
| --- | --- | --- | --- |
| Patient/Primary Care Team Level |  |  |  |
| Local (hospital or clinic) VA Level |  |  |  |
| National VA Level |  |  |  |
