# Supplementary material for: Developing Strategies to Reduce Unnecessary Services in Primary Care: Protocol for User-Centered Design Charrettes
Source: JMIR Res Protoc. 2019 Nov 26;8(11):e15618. doi: 10.2196/15618 (PMC6904896; doi:10.2196/15618)
Supplement: Multimedia Appendix 7 [file resprot_v8i11e15618_app7.docx]

| **Carotid Artery Stenosis Screening** | | | | | |
| --- | --- | --- | --- | --- | --- |
|  | **Patient Description** | **Provider Description** | **Wants/Needs/etc.** | **Because (motivations)** | **But (barriers)** |
|  | ***Patient – not resistant*** | ***Provider – not resistant*** |  |  |  |
|  |  |  |  |  |  |
|  | ***Patient – not resistant*** | ***Provider – somewhat resistant*** |  |  |  |
| 1 | - Dennis is a 64-year-old retired mechanic - Has a very close relationship with his siblings and their children - Only goes to the doctor when a problem arises, so typically goes to appointments with specific things to discuss with the doctor | - Dr. Walker is a primary care provider at a large VAMC - Has been Dennis’s provider for the last 3 years | Dennis does not want to get screened for carotid artery stenosis | because his brother was screened recently and had a false positive result, which required many follow-up tests, | but Dr. Walker recommends Dennis be screened because Dr. Walker feels pressured by local specialists to be aggressive about screening. |
| 2 | - Cynthia is 62 years old and has a second career as a florist - Leads a healthy lifestyle, including swimming almost every day of the week - She doesn’t like going to the doctor because she feels it just leads to unnecessary tests | - Dr. Baker is a physician at a VA community clinic - Has been practicing medicine for over 35 years - Has been Cynthia’s physician for the last 2 years | Cynthia is not interested in getting screened for carotid artery stenosis | because she believes the screening is unnecessary and takes a lot of time and money | but when Dr. Baker started practicing medicine many years ago the screening was common. Dr. Baker finds it difficult to change how s/he practices now and still recommends screening for most patients like Cynthia. |
|  |  |  |  |  |  |
|  | ***Patient – not resistant*** | ***Provider – very resistant*** |  |  |  |
| 3 | - Greg is 63 years old and a retired Navy pilot - Staying healthy is important to him - Subscribes to many magazines (e.g., Men’s Health) and online blogs that are health related - Comes prepared to his doctor’s appointment | - Dr. Hill is a primary care physician at a small VA hospital - Greg is a new patient | Greg does not want to get a carotid artery screen | because he read an article that said it is not necessary to have screening if you don’t have symptoms or a history of stroke | but Dr. Hill recently had a patient die from a stroke and wants Greg to get screened. |
| 4 | - Sharon is 52 years old and works at a busy accounting firm - Stays active and enjoys a vegetarian only diet - Her coworker mentioned that there was going to be a mobile stroke screening clinic coming to their workplace soon and this reminded Sharon that Dr. Collins brought the topic up at her last appointment | - Dr. Collins is a primary care provider at a VA community clinic - Will be retiring at the end of the year - Has been Sharon’s provider for about 3 years | Sharon wants to talk with Dr. Collins about carotid artery screening and reiterate her lack of interest in ever getting screened | because she believes the screening isn’t necessary given that she is physically active, doesn’t smoke, and has a healthy diet | but, during their conversation, Dr. Collins tells Sharon that they should be more aggressive and screen to be thorough and ensure she doesn’t have plaque building up in her arteries. |
|  |  |  |  |  |  |
|  | ***Patient – somewhat resistant*** | ***Provider – not resistant*** |  |  |  |
| 5 | - Charles is a 65-year-old, retired plant manager - Lives in Michigan 6 months of the year and Florida the other 6 months - Enjoys playing golf with his buddies | - Dr. Malone is a physician at a large VAMC - Has been seeing Charles (during the months he is in Michigan) for at least 5 years | Dr. Malone does not want Charles to have a carotid artery screening | because Dr. Malone doesn’t think Charles will benefit from having one and is concerned about the downsides | but Charles’ other physician in Florida recommends screening. Charles is unsure what to do. |
| 6 | - Peter is a 61-year-old salesman - He has an old foot injury that makes it difficult for him to exercise - He is somewhat overweight - His father had a stroke | - Dr. Green is a primary care provider at a large VAMC - Has been seeing Peter regularly for 3 years | Dr. Green does not want Peter to have an ultrasound of the carotid arteries | because Dr. Green wants to focus on the screening tests that are known to be effective, and this isn’t one of them, | but Peter is hesitant to not get carotid artery screening because he worries about having a stroke like his father. |
|  |  |  |  |  |  |
|  | ***Patient – somewhat resistant*** | ***Provider – somewhat resistant*** |  |  |  |
| 7 | George is 62 years old and used to work in construction with his son  Suffers from high blood pressure, type 2 diabetes, and chronic pain  Prefers to not do unnecessary tests or take unnecessary medication, but will typically do what his doctor recommends | - Dr. Jones is a primary care provider at a small VA community clinic - Has been practicing medicine for the last 12 years and is close to retirement - Has been seeing George for the last 2 years | George’s preference, if asked, would be to not get screened for carotid artery stenosis | because he doesn’t like medical tests | but Dr. Jones believes that most patients like for him to be very thorough with ordering tests and screens that could be helpful, so Dr. Jones simply moves forward with ordering the screening. |
| 8 | Darius is 66 years old and drives a semi-truck cross country  Skeptical of the VA because his friends tell him that ‘VA care more about cutting costs than helping Veterans’  Has had some issues with his VA benefits recently and is frustrated with how long it takes to get an answer | - Dr. Holmes is a primary care provider at a VAMC - Has seen Darius for the past 5 years | Dr. Holmes does not want to give Darius a carotid artery screening | because Dr. Holmes knows the harms of screening outweigh the benefits | but Dr. Holmes is worried that if he does not offer the screening to Darius, Darius will think he is just trying to save the VA money and it might damage their relationship. |
|  |  |  |  |  |  |
|  | ***Patient – somewhat resistant*** | ***Provider – very resistant*** |  |  |  |
| 9 | - Donald is 53 years old and owns an auto body shop - Often spends long evenings in the shop with his military buddies and his dog - Recently received a pamphlet from his local minute clinic that discussed the benefits of a carotid artery screening | - Dr. Patterson is a physician at a VAMC - Donald has been a patient for about 2 years - Heard a briefing from a VA stroke prevention program that recommends not conducting carotid artery screening in patients like Donald | Donald isn’t sure whether he should have a carotid artery screening | because Donald doesn’t have enough knowledge about when someone should or shouldn’t get screened for carotid artery stenosis | but Dr. Patterson doesn’t agree with the VA stroke prevention program and is a strong advocate for preventing strokes with early detection, so orders a carotid artery ultrasound for Donald. |
| 10 | Fatima is a 60-year-old teacher  Does not enjoy the stress and anxiety that is associated with medical tests  Always listens to what the doctors recommend | - Dr. Franklin is a provider at a large VAMC - Has been seeing Fatima for the last 2 years | Fatima really does not want to be screened for carotid artery stenosis | because she doesn’t think it is necessary and dislikes the anxiety associated with having tests | but Fatima does not know how to bring up her concerns to Dr. Franklin so she goes along with Dr. Franklin’s strong recommendation to be screened. |
|  |  |  |  |  |  |
|  | ***Patient – very resistant*** | ***Provider – not resistant*** |  |  |  |
| 11 | - Elizabeth is a 66-year-old retired lawyer - She recently moved to Southeast Michigan to be closer to her parents - Takes care of her mother, who has dementia - Prefers to make the final decision about her healthcare, with some input from doctors | - Dr. Clark is a primary care physician at a VAMC - Has been practicing medicine at the VA for the last 14 years - This is the 2^nd^ visit with Elizabeth | Dr. Clark doesn’t want Elizabeth to have an ultrasound to test for carotid artery stenosis | because Dr. Clark doesn’t think Elizabeth will benefit from them and they carry risks | but Elizabeth doesn’t totally trust Dr. Clark yet, because this is only her 2^nd^ visit, so insists on getting a carotid artery screening. |
| 12 | - Frank is 55 years old and retired from the Air Force - He wants to do everything possible to stay healthy - Frank and his partner work out every morning at a local gym | - Dr. Peterson is a primary care provider at a VA community clinic - Has been seeing Frank on and off for the last 5 years | Dr. Peterson tells Frank that he does not need a carotid artery screening | because Dr. Peterson believes the harms outweigh the benefits | but Frank wants reassurance that he doesn’t have plaque build-up and so he pushes for Dr. Peterson to order the test for him. |
|  |  |  |  |  |  |
|  | ***Patient – very resistant*** | ***Provider – somewhat resistant*** |  |  |  |
| 13 | - Hakeem is 67 years old and retired from the Army - Is very active in his local American Legion - His mother lived to be 102 and Hakeem leads a healthy lifestyle in order to live just as long | - Dr. Foster is a primary care provider at a VAMC - Just finished residency, has only been practicing medicine for a year | Hakeem wants to get screened for carotid artery stenosis | because he believes that screening can definitely help him live longer | but Dr. Foster is worried that he won’t be able to convince Hakeem that carotid artery screenings are not beneficial because Hakeem so strongly believes that carotid artery screening is a good idea. |
| 14 | - Carl is 75 years old and a retired accountant - His wife recently had a stroke and is in a nursing home - Carl wants to talk with Dr. Rice about having a carotid ultrasound himself - Is worried how he is going to handle things (healthcare, home chores, bills, etc.) now that his wife is in the home | - Dr. Rice is a primary care provider at a large VAMC - Has been seeing Carl for the last 10 years | Dr. Rice would typically not recommend carotid artery screening | because the harms outweigh the benefits in patients without symptoms | but Dr. Rice is worried that if he tells Carl not to have the screening then Carl will think that he is giving up on him. |
|  |  |  |  |  |  |
|  | ***Patient – very resistant*** | ***Provider – very resistant*** |  |  |  |
| 15 | - Michael is 81 years old and retired from the Marines - Has never had any major health concerns - Is ‘set in his ways’ and wants to do everything he can to remain healthy | - Dr. Parks is a physician at a small VA community clinic - Has seen Michael on-and-off for 20 years - Is retiring in the next few months | Experts in stroke care strongly encourage providers to avoid carotid artery screenings to patients without a history of stroke or without symptoms (that is, do not screen patients like Michael) | because screening is not beneficial and can even be harmful | but Dr. Parks decides to screen Michael because screening for stroke is what he considers ‘good quality’ care. Furthermore, Dr. Parkers is quite certain Michael would want to get a screening as well. |

| **Colorectal Cancer Screening** | | | | | |
| --- | --- | --- | --- | --- | --- |
|  | **Patient Description** | **Provider Description** | **Wants/Needs/etc.** | **Because (motivations)** | **But (barriers)** |
|  | ***Patient – not resistant*** | ***Provider – not resistant*** |  |  |  |
|  |  |  |  |  |  |
|  | ***Patient – not resistant*** | ***Provider – somewhat resistant*** |  |  |  |
| 1 | - Dennis is an 82-year-old retired mechanic - He is due for his next colonoscopy - Really doesn’t like getting colonoscopies, and would prefer to not have to get one again | - Dr. Walker is a primary care provider at a large VAMC - Has been Dennis’s provider for the last 3 years | Dennis does not want to get a colonoscopy | because he thinks the prep is too hard (and he even mentions that “I’m getting too old for this”) | but Dr. Walker didn’t have enough time to discuss a colonoscopy and isn’t aware of how Dennis feels, so Dr. Walker simply moves forward with scheduling the next colonoscopy. |
| 2 | - Cynthia is 62 years old and has a second career as a florist - Receives regular colonoscopies every 3 years - She would like to get colonoscopies less frequently | - Dr. Baker is a physician at a VA community clinic - Has been Cynthia’s physician for the last 2 years | Dr. Baker thinks Cynthia should have colonoscopies every 10 years, instead of every 3 years, | because Dr. Baker doesn’t believe Cynthia will benefit from having them so frequently | but Dr. Baker ultimately decides not to discuss it because s/he assumes Cynthia will want to continue having colonoscopies every 3 years anyway. |
|  |  |  |  |  |  |
|  | ***Patient – not resistant*** | ***Provider – very resistant*** |  |  |  |
| 3 | - Greg is 81 years old and retired from the Navy - He currently receives regular colonoscopies - Greg always does a lot of reading and research and comes prepared to his doctor’s appointment | - Dr. Hill is a primary care physician at a small VA hospital - Greg is a new patient | Greg does not want to get colonoscopies any longer | because Greg read an article that said it is not necessary for a person over 80 years old to have colonoscopies | but Dr. Hill recently had a patient die from colon cancer and wants Greg to continue screening if there is even a small chance of benefit. |
| 4 | - Sharon is 67 years old and owns her own bakery - She tries hard to be healthy (takes medications as prescribed, eats healthy, sees doctor’s regularly) - Receives colonoscopies every 5 years | - Dr. Collins is a primary care provider at a VA community clinic - Will be retiring at the end of the year - Has been Sharon’s provider for about 3 years | Sharon wants to talk with Dr. Collins about receiving colonoscopies less frequently | because she was recently talking with a friend who mentioned that her own doctor typically recommends colonoscopies be done every 10 years | but Dr. Collins tells Sharon he would like to be more aggressive and continue colorectal screening every 5 years, to ensure she does not get cancer. |
|  |  |  |  |  |  |
|  | ***Patient – somewhat resistant*** | ***Provider – not resistant*** |  |  |  |
| 5 | - Charles is 64 years old and works as a plant manager - Lives in Michigan 6 months of the year and Florida the other 6 months - Receives regular colonoscopies every 5 years | - Dr. Malone is a physician at a large VAMC - Has been seeing Charles (during the months he is in Michigan) for at least 5 years | Dr. Malone wants Charles to have a colonoscopy every 10 years | because Dr. Malone doesn’t think Charles will benefit from having them as frequently as every 5 years | but Charles’ other physician in Florida recommends screening every 5 years. Charles is unsure what to do. |
| 6 | - Darius is a 81-year-old retired welder - While he generally listens to his doctor, he also does a lot of reading and goes to appointments with his own opinions - He receives regular colonoscopies | - Dr. Green is a primary care provider at a large VAMC - Has been seeing Darius regularly for 3 years | Dr. Green wants Darius to stop having colonoscopies | because Dr. Green doesn’t think Darius will benefit from them anymore given his age | but Darius is hesitant to stop getting colonoscopies because many articles he reads talk about the importance of screening for cancer. |
|  |  |  |  |  |  |
|  | ***Patient – somewhat resistant*** | ***Provider – somewhat resistant*** |  |  |  |
| 7 | George is 81 years old and used to work in construction with his son  Suffers from high blood pressure, type 2 diabetes, and chronic pain  He had a colonoscopy 10 years ago  Would like to stop colonoscopies, but will continue getting them for as long as his doctors tells him | - Dr. Jones is a primary care provider at a small VA community clinic - Has been practicing medicine for the last 12 years - Has been seeing George for the last 2 years | George is really hoping that Dr. Jones recommends stopping colonoscopies | because they are uncomfortable | but Dr. Jones recommends George continue because Dr. Jones feels pressured by local specialists to be aggressive about screening. |
| 8 | Stacey is a 60-year-old teacher  Has received regular colonoscopies every 5 years  She really does not enjoy colonoscopies, and would prefer to get them less frequently  Always listens to what the doctors recommend | - Dr. Franklin is a provider at a large VAMC - Has been seeing Stacey for the last 2 years - Only makes changes to patients’ care when they have a complaint, or side effect | Stacey wants to discuss getting colonoscopies less frequently with Dr. Franklin | because drinking the prep makes her feel sick | but Stacey does not know how to bring it up to Dr. Franklin and doesn’t want to be seen as a difficult patient. |
|  |  |  |  |  |  |
|  | ***Patient – somewhat resistant*** | ***Provider – very resistant*** |  |  |  |
| 9 | - Donald is 65 years old and owns an auto body shop - He receives regular colonoscopies every 5 years - Recently heard that colonoscopies should only be done every 10 years | - Dr. Patterson is a physician at a VAMC - Donald has been a patient for about 2 years - Heard a briefing from a VA cancer screening safety program that recommends reducing the frequency of colonoscopies to every 10 years in patients like Donald | Donald isn’t sure whether he should reduce the frequency of his colonoscopies | because Donald doesn’t have enough knowledge about when someone should or shouldn’t get a colonoscopy | but Dr. Patterson doesn’t agree with the VA cancer screening safety program and is a strong advocate for fighting cancer with early detection, so Donald continues screening every 5 years. |
| 10 | - Fatima is a 63-year-old artist - Receives regular colonoscopies every 5 years - She knows how important colonoscopies are for early detection, but doesn’t like having them so frequently - Cautious of the VA because her friends tell her that the ‘VA cares more about cutting costs than helping Veterans’ - Likes to have control over her own healthcare | - Dr. Hughes is a primary care provider at a VA community clinic - Has had Fatima as a patient for several years | Fatima is considering talking with Dr. Hughes about reducing the frequency of her colonoscopies | because, although they can detect cancer early, they are uncomfortable and take a lot of time | but Dr. Hughes doesn’t bring the issue up during the clinic visit because Fatima has been a challenging patient in the past and Dr. Hughes doesn’t want to stir up any new problems. |
|  |  |  |  |  |  |
|  | ***Patient – very resistant*** | ***Provider – not resistant*** |  |  |  |
| 11 | - Elizabeth is an 81-year-old retired lawyer - She recently moved to Southeast Michigan to be closer to her grandkids - Received regular colonoscopies from her previous provider | - Dr. Clark is a primary care physician at a VAMC - Has been practicing medicine at the VA for the last 14 years - This is the 2^nd^ visit with Elizabeth | Dr. Clark doesn’t want Elizabeth to get colonoscopies anymore | because Dr. Clark doesn’t think Elizabeth will benefit from them and they carry risks | but Elizabeth doesn’t totally trust Dr. Clark yet, because this is only her 2^nd^ visit, so insists on getting a colonoscopy. |
| 12 | - Frank is 84 years old and retired from the Air Force - Has received regular colonoscopies - Frank always wants to do everything possible to stay healthy (wants to be around for as long as possible!) - His best friend was diagnosed with colorectal cancer and has had a lot of problems related to his cancer | - Dr. Peterson is a primary care provider at a VA community clinic - Has been seeing Frank on and off for the last 5 years | Dr. Peterson tells Frank that colonoscopies are no longer needed | because Dr. Peterson believes the harms outweigh the benefits | but Frank is scared that he will end up like his friend, so he pushes for a colonoscopy. |
|  |  |  |  |  |  |
|  | ***Patient – very resistant*** | ***Provider – somewhat resistant*** |  |  |  |
| 13 | - Hakeem is 81 years old and retired from the Army - He gets regular colonoscopies. - His mother lived to be 102 and Hakeem leads a healthy lifestyle in order to live just as long | - Dr. Foster is a primary care provider at a VAMC - Just finished residency, has only been practicing medicine for a year | Hakeem wants to continue getting regular colonoscopies | because he believes that screening can definitely help him live longer | but Dr. Foster is worried he won’t be able to convince Hakeem why stopping colonoscopies is best because Hakeem strongly believes that colon cancer screening is a good idea. |
| 14 | - Carl is 84 years old and a retired accountant - Received regular colonoscopies - Recently lost his wife to cancer - The negative screening results give him comfort that he is cancer free, so he doesn’t want to stop having colonoscopies | - Dr. Rice is a primary care provider at a large VAMC - Has been seeing Carl for the last 10 years | Dr. Rice would typically stop colonoscopies for a patient like Carl | because it is not recommended for older patients | but Dr. Rice is worried that if he tells Carl to stop then Carl will think he is giving up on him. |
|  |  |  |  |  |  |
|  | ***Patient – very resistant*** | ***Provider – very resistant*** |  |  |  |
| 15 | - Michael is 81 years old and retired from the Marines - Has been having regular colonoscopies - Is “Set in his ways” and plans to continue getting colonoscopies for the rest of his life | - Dr. Parks is a physician at a small VA community clinic - Has seen Michael on-and-off for 20 years - Is retiring in the next few months | Experts in colorectal care strongly encourage providers to stop screening patients after the age of 80 (like Michael) | because screening these patients is not beneficial and can even be harmful | but Dr. Parks decides to continue to screen Michael because screening for cancer is what he considers ‘good quality’ care. Also, Dr. Parks is quite certain Michael doesn’t want to stop getting colonoscopies. |

|  | **DIABETES TREATMENT** | | | | |
| --- | --- | --- | --- | --- | --- |
|  | **Patient Description** | **Provider Description** | **Wants/needs/etc.** | **Because (motivation)** | **But (barrier(s))** |
|  | *Patient – not resistant to de-int* | *Provider – not resistant* |  |  |  |
|  |  |  |  |  |  |
|  | *Patient – not resistant to de-int* | *Provider – Somewhat resistant* |  |  |  |
| **1** | - Jim is 67 years old and owns a small bike repair business - On 12 medications, including insulin for his diabetes, and would be very happy to stop or reduce any of them - Additional diagnoses include: depression, back pain, substance abuse, heart disease, high blood pressure, and several other mental health conditions - Most recent A1c level is low | - Dr. Postma is a staff physician at a VA Community Clinic - Served as Jim’s doctor for over 7 years | Dr. Postma wants to scale back Jim’s diabetes medication | because Jim’s blood sugar level is too low and might even be harmful at his age | but Dr. Postma doesn’t discuss it with Jim during his clinic visit because the discussion will take a lot of time and there are other higher priorities |
| **2** | - Caroline is 72 years old and is a retired teacher - Was diagnosed with Type 2 diabetes 20 years ago and has been on insulin for most of those years - Really doesn’t like giving herself shots or taking medication, but never complains to the doctor - Several recent A1c’s have been low | - Dr. Young is a primary care provider at a large VAMC - Has been Caroline’s provider for 3 years | Dr. Young ponders whether to talk with Caroline about reducing her insulin | because her last two A1c measurements have been low | but Dr. Young ultimately decides not to discuss it because s/he assumes Caroline will not want to reduce her insulin anyway |
|  |  |  |  |  |  |
|  | *Patient – not resistant to de-int* | *Provider – Very resistant* |  |  |  |
| **3** | - Darius is 71 years old and retired from the Navy - He is currently on multiple medications to help control his diabetes - Darius always does a lot a reading and research and comes prepared to his doctor’s appointment - Most recent A1c level is low | - Dr. Stewart is a primary care physician at a small VA hospital - Sees patients at the University Hospital 2 days/week and at the VA 3 days/week - Darius is a new patient | Darius wants to reduce the dose of one of his diabetes medications | because an article that he just read stated that: 1) a low A1c can be harmful for older adults, and 2) reducing medications can bring the A1c back up to safe levels | but Dr. Stewart doesn’t want to change any of Darius’s medications because he thinks his blood sugar is well controlled |
| **4** | - Delores is 89 years old and has raised 8 children - Many chronic conditions, including diabetes, breast cancer, and severe lung disease - Tries hard to be healthy (takes medications as prescribed, eats healthy, sees doctor’s regularly) - Lives with daughter and son-in-law - Most recent A1c level is low | - Dr. Mann is a primary care provider at a VA Community Clinic - Will be retiring at the end of the year - Has been Delores’ provider for about 3 years | Delores wants to talk with Dr. Mann about possibly reducing her diabetes medications | because her daughter told her that a constant low blood sugar can cause many problems (and she plans to stay on this earth as long as possible!) | but Dr. Mann believes ‘insulin will control Delores’ diabetes’ and indicates that they need to work hard to make sure her diabetes and quality of life are as good as they can be |
|  |  |  |  |  |  |
|  | *Patient – Somewhat resistant* | *Provider – Not resistant* |  |  |  |
| **5** | - Richard is 66 years old and drives a semi-truck cross country - Recently started dieting and lost some weight - Frequently misses medical appointments - Most recent A1c level is low | - Dr. Holmes is primary care provider at a VAMC - Has seen Richard for the past 5 years - Works closely with VA case managers; the case managers help patients reduce their insulin doses when needed | Dr. Holmes wants Richard to see the case manager (so the case manager can help Richard cut back on his insulin dose) | because Richard’s A1c is lower than recommended and a low A1c can be harmful in older adults | but reducing insulin is a process that takes time and effort (i.e., reduce insulin dose THEN get A1c measured THEN determine whether to reduce insulin more THEN repeat) and Richard often misses his VA clinic visits and lab tests |
| **6** | - Fatima is 77 years old and worked as a postmaster for many years - Taking multiple oral diabetes medications - Lives in Michigan 6 months of the year and Florida the other 6 months - Has had some mild dizziness over the past year - Most recent A1c level is low | - Dr. Lee is a physician at a VA community clinic - He has been practicing medicine for the last 12 years - Has been seeing Fatima (during the months she is in Michigan) for the last 5 years | Dr. Lee wants to take Fatima off one her current diabetes medications | because Fatima’s dizziness may be due to her low blood sugar level | but Fatima’s other primary care doctor in Florida didn’t mention that she should stop a medication so Fatima is unsure what to do |
|  |  |  |  |  |  |
|  | *Patient – Somewhat resistant* | *Provider – Somewhat resistant* |  |  |  |
| **7** | - Gerald is 69 years old and retired from the Air Force as a Chief Master Sergeant - Takes several medications for diabetes and heart disease - Dislikes having to give himself insulin shots (1 in the morning and 1 at night) - Skeptical of the VA because his friends tell him that ‘VA cares more about cutting costs than helping Veterans’ - Most recent A1c level is low | - Dr. Franklin is a provider at a large VAMC - Just finished residency and started at the VA last month | Dr. Franklin notices that Gerald’s A1c is low and considers talking to Gerald about reducing his insulin to 1 shot per day | because having low blood sugar can be harmful in older adults | but Dr. Franklin is worried that s/he will not be able to effectively communicate the reasons to stop or reduce medications and that Gerald  may just think the VA is trying to save money |
| **8** | - George is 80 years old and worked all his life in construction - Was diagnosed with Type 2 diabetes 18 years ago - Recently lost his wife to cancer, so has been somewhat depressed - Believes that if things are going well then you probably should just keep things as is - Most recent A1c level is low, but George hasn’t had any symptoms | - Dr. Jones is a Primary care provider at a VA Community Clinic - He has been practicing medicine for the last 12 years - Has been seeing George for the last 2 years | Dr. Jones is well aware of the recommendation to reduce diabetes medications in older adults with a low A1c | because they are not beneficial and may even be harmful | but Dr. Jones is worried that if he tells his patient to stop taking a medication than the patient will think he is giving up on him |
|  |  |  |  |  |  |
|  | *Patient – Somewhat resistant* | *Provider – Very resistant* |  |  |  |
| **9** | - Donald is 65 years old and works as a plant manger - Was diagnosed with Type 2 diabetes 10 years ago - Being on medications gives him some reassurance that his health is ok - Follows his doctor’s advice - Received a pamphlet from a VA diabetes safety program that recommends patients discuss cutting back diabetes medications with their doctor - Most recent A1c level is low | - Dr. Patterson is a physician at a VAMC - Donald has been his patient for about 2 years - Received a pamphlet from a VA diabetes safety program that recommends cutting back diabetes medications in patients like Donald | Donald isn’t sure whether cutting back on his diabetes medication is appropriate | because he still doesn’t have enough knowledge about when you should or shouldn’t decrease medications | but Dr. Patterson doesn’t agree with the VA diabetes safety program because he had several bad experiences decreasing medications in other patients, so he doesn’t change Donald’s medications |
| **10** | - Victor is 67 years old and a retired train conductor - On two oral diabetes medications and insulin - Sees an outside specialist for some kidney issues - His outside specialist mentioned that low blood sugar levels in older adults can be harmful and suggested he discuss reducing his diabetes medications with Dr. Hughes - Most recent A1c level is low | - Dr. Hughes is a primary care provider at a VA Community Clinic - Victor has been his patient for several years | Victor is not sure he wants to talk with Dr. Hughes about reducing his diabetes medications | because he has been feeling great the past few months | but Dr. Hughes doesn’t bring the issue up during the clinic visit anyway because he strongly believes that aggressive diabetes treatment is appropriate and Dr. Hughes is happy with Victor’s A1c level |
|  |  |  |  |  |  |
|  | *Patient – Very resistant* | *Provider – Not resistant* |  |  |  |
| **11** | - Arik is 74 years old and retired from the Army - Suffers from diabetes, hypertension, kidney disease, and hearing loss - Recently transferred to the VA, with several prescriptions including insulin - Most recent A1c level is low | - Dr. Stokes is a physician at a large VAMC - Has been seeing Arik for 1 year | Dr. Stokes wants Arik to reduce his insulin dose | because Arik’s A1c is low and current evidence suggests that a low A1c can be harmful in older adults | but Arik has been on insulin for many years and is scared his blood sugars will rise if he stops or reduces it, so he refuses |
| **12** | - Elizabeth is 68 years old and recently sold her floral business - Was diagnosed with diabetes 5 years ago - Recently moved to Southeast Michigan from the Upper Peninsula and is starting to receive care at a VAMC - Most recent A1c level is low | - Dr. Clark is a primary care physician at a VAMC - Has been practicing medicine at the VA for the last 14 years - This is the 2^nd^ visit with Elizabeth | Dr. Clark wants to reduce the dose of Elizabeth’s diabetes medication | because Dr. Clark worries about Elizabeth experiencing dangerously low blood sugar | but Elizabeth doesn’t fully trust Dr. Clark because this is only her 2^nd^ visit with the doctor and so refuses |
|  |  |  |  |  |  |
|  | *Patient – Very resistant* | *Provider – Somewhat resistant* |  |  |  |
| **13** | - Kathleen is 75 years old and was a stay at home mom - Was diagnosed with Type 2 diabetes at age 58 - Has been on the same medications for 8 years - 5 years ago, she was having low blood sugars and Dr. King reduced her insulin over several months, but her A1c eventually just went back up and she remembers not feeling well at all during that period - Does not want to go through reducing insulin again - Most recent A1c level is low | - Dr. King is a primary care provider at a small VA community clinic - Has seen Kathleen for about 3 years | Dr. King believes that trying to reduce Kathleen’s insulin dose again is a good idea | because her A1c is still low and Dr. King worries about dangerously low blood sugars | but Dr. King was not successful reducing Kathleen’s medication last time so he doesn’t bring up his current concerns out of fear of damaging the relationship |
| **14** | - Hakeem is 81 years old and a retired electrician - Diabetic for many years and takes two types of insulin - Has occasional low blood sugars at home, with some symptoms - Doesn’t like change - Most recent A1c level is low | - Dr. Engels is a new physician - Joined the VA 1.5 years ago - Been Hakeem’s doctor for only 1 year - No experience in reducing insulin doses | Dr. Engels considers having Hakeem start reducing his insulin dose | because Hakeem’s blood sugar levels are too low at times, causing him to feel dizzy and have cold sweats, | but Hakeem has gotten used to his injections and believes that staying on insulin will have future benefits for him. Also, there aren’t any quick guidelines to follow to help with reducing insulin, so Dr. Engels doesn’t press the issue at the clinic visit. |
|  |  |  |  |  |  |
|  | *Patient – Very resistant* | *Provider – Very resistant* |  |  |  |
| **15** | - Bill is 85 years old and retired from the Marines - Has had diabetes for as long as he can remember - Is “Set in his ways” and very happy with his current blood sugar level - Most recent A1c level is low | - Dr. Bennin is a physician at a small VA Community Clinic - Has seen Bill on-and-off for 20 years - Is retiring in the next few months | Experts in diabetes care strongly encourage providers to reduce diabetes medications in older patients (like Bill) | because low blood sugar for these patients is not beneficial and can even be harmful | but Dr. Bennin decides to keep things as is with Bill because he doesn’t want Bill’s A1c to rise above a target A1c that is generally considered ‘good quality care’ in guidelines. Furthermore, Dr. Bennin is quite certain that Bill will not want to reduce his medications anyway. |
