# Supplementary figures and images for: Developing Strategies to Reduce Unnecessary Services in Primary Care: Protocol for User-Centered Design Charrettes
Source: JMIR Res Protoc. 2019 Nov 26;8(11):e15618. doi: 10.2196/15618 (PMC6904896; doi:10.2196/15618)

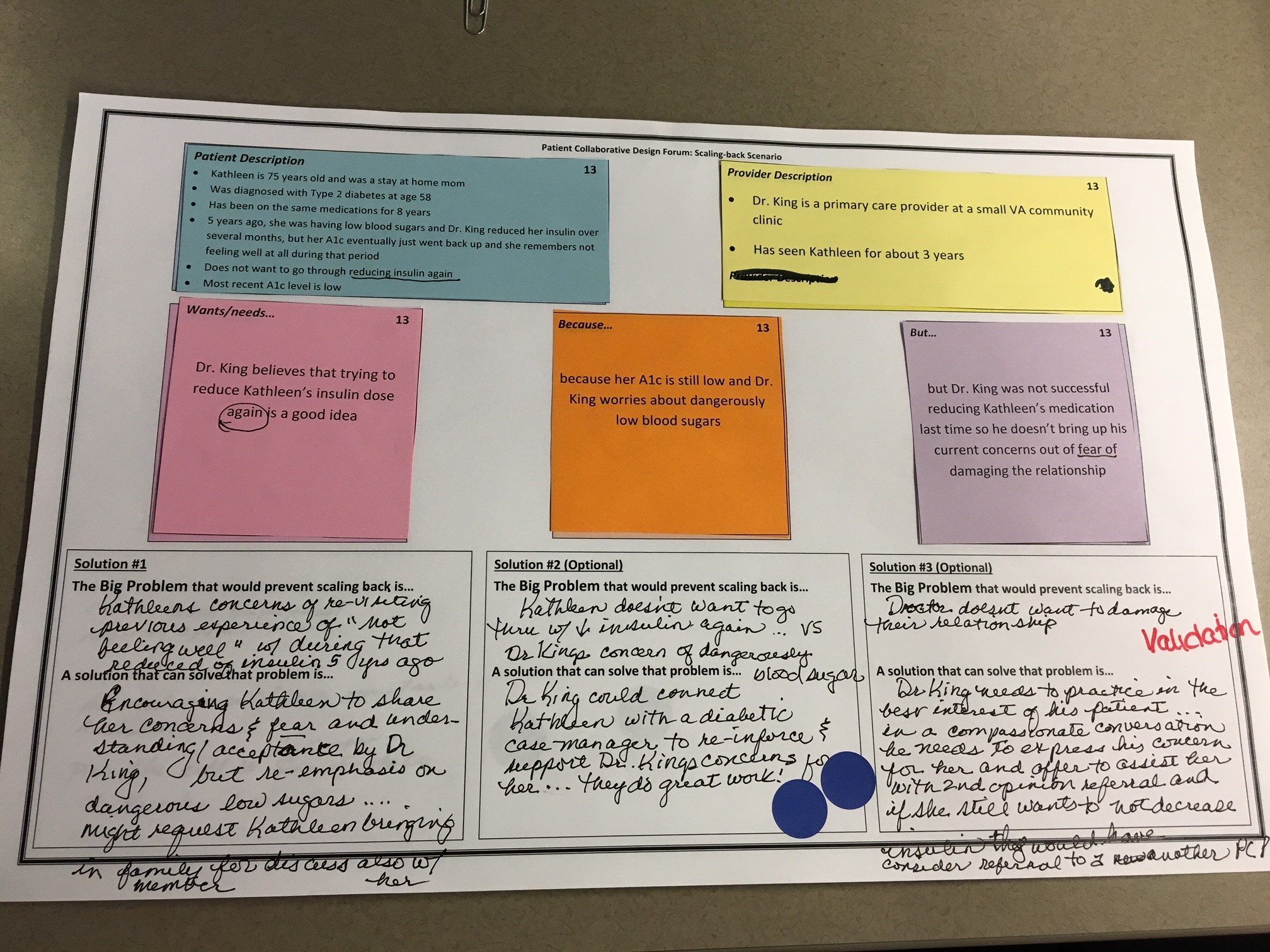

Supplement: Multimedia Appendix 8 [file resprot_v8i11e15618_app8.docx]
