# Supplementary material for: Developing Strategies to Reduce Unnecessary Services in Primary Care: Protocol for User-Centered Design Charrettes
Source: JMIR Res Protoc. 2019 Nov 26;8(11):e15618. doi: 10.2196/15618 (PMC6904896; doi:10.2196/15618)
Supplement: Multimedia Appendix 9 [file resprot_v8i11e15618_app9.docx]

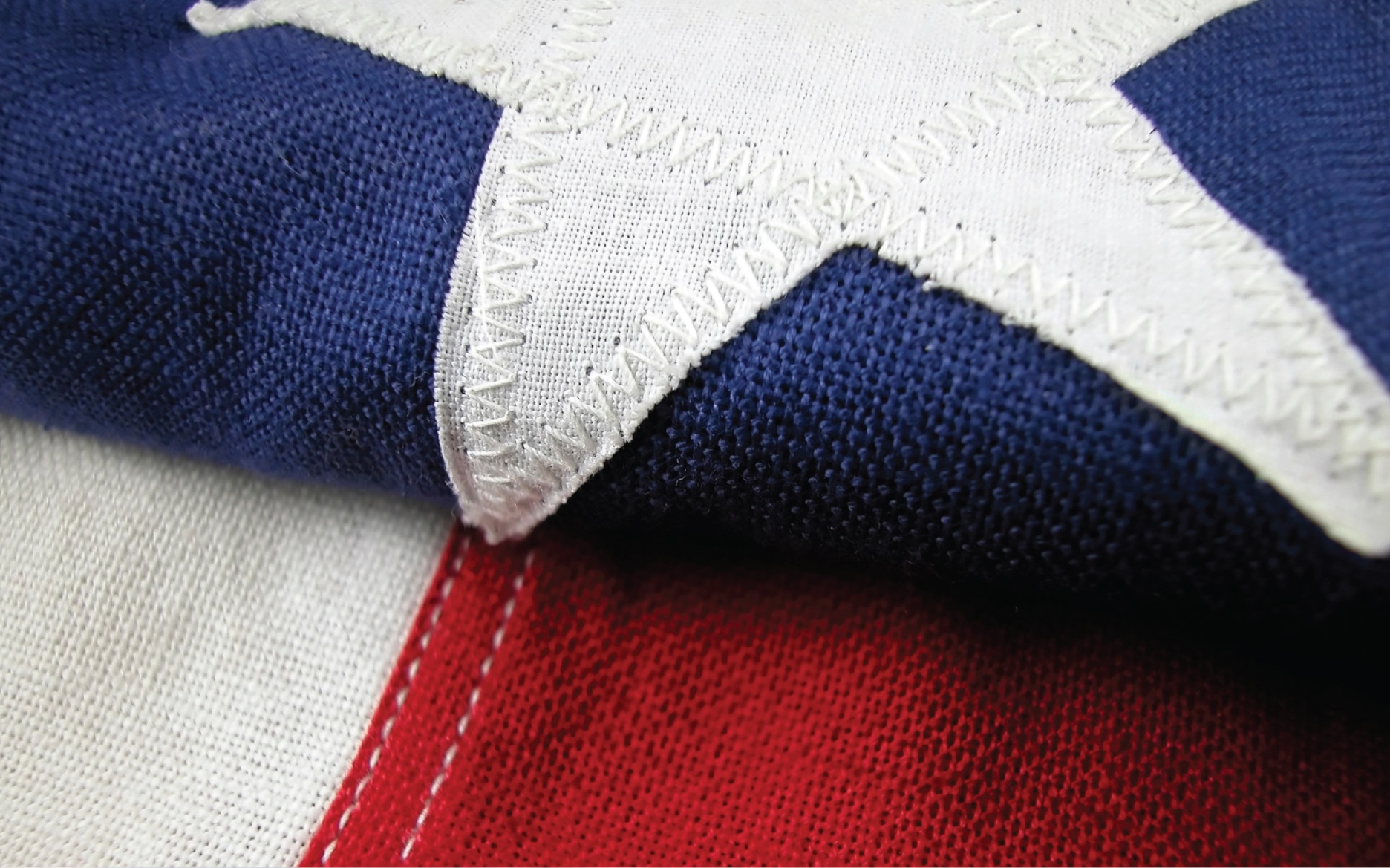


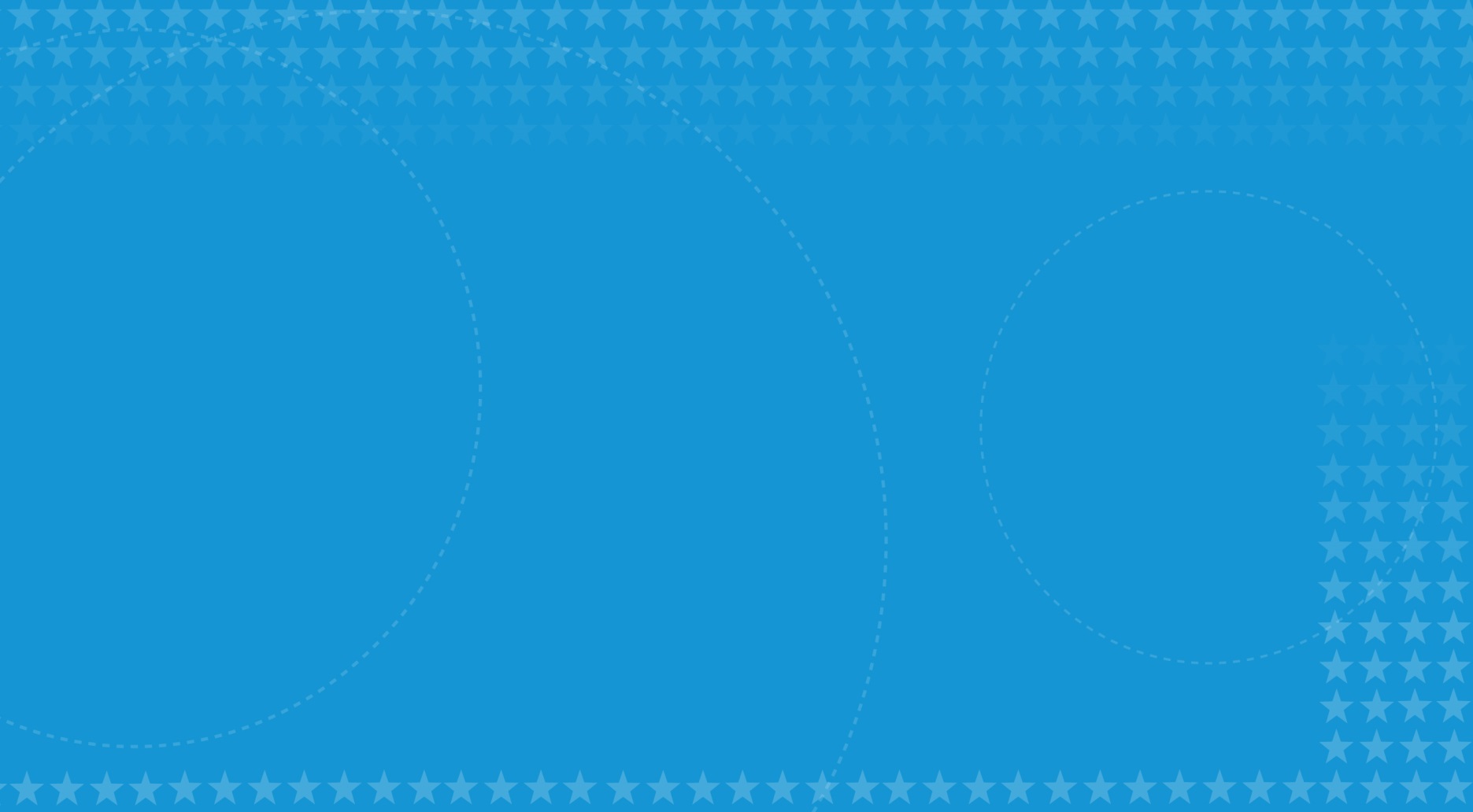


**Phase 2 Forum**

**Facilitator Guide**

**Agenda**

| **Time** | **Activity** |
| --- | --- |
| 3:30-4:05 | Registration, Consent, and Pre-Forum Survey  **We’ll start at 5 minutes after 4pm to allow everyone time to find the room!* |
| 4:05-4:15 | Welcome, Overview, and Agenda |
| 4:15-4:40 | Introductions within Sub-Groups   - Participants read experience commentary cards and read case |
| 4:40-4:50 | Super Strategies Review   - Sub-groups review super strategies |
| 4:50-6:20 | WhoDo   - Sub-groups discuss the people and actions involved in de-intensification, as well as potential barriers, and solutions |
| 6:20-6:40 | Dinner |
| 6:40-6:50 | Instructions for the Next Activities |
| 6:50-7:00 | Most Valuable Action   - Sub-groups select the most valuable action |
| 7:00-7:50 | WhoDo Refinement   - Sub-groups reflect and discuss in further detail the components and actions of the most valuable action |
| 7:50-8:00 | Thank you, Post-Forum Survey, Gift Cards, and Consents |

| **Facilitation Tips** | |
| --- | --- |
| **If group members…** | **Then you might…** |
| Contribute relevant ideas | - Recognize contribution and continue moving discussion forward. |
| Seem unfocused/are goofing around   - People become undisciplined when they are overloaded or worn out. | - Ask the group to **take a break**, “let’s take a break.” - Add positive statement about what’s been accomplished so far. - Then, **emphasize the goals** of the small group discussion. |
| Interrupt each other while speaking   - This could be a good sign of engagement in the discussion, but it may also require some direction from you | - If someone is interrupted, offer them the opportunity to finish what he/she was saying. - It may also be an appropriate time to reaffirm some of the ground rules for discussion.   - **You can say:** “I want to make sure that I can hear everyone’s ideas and opinions. It would be really helpful if we could have 1 person talk at a time. If more than one person wants to talk at the same time, I’ll ask you to raise hands and I’ll number you off. That way, you’ll know when your turn is coming.” |
| Are being repetitious   - People repeat themselves because they don’t feel heard. | - Use **paraphrasing** to help that person summarize his or her thinking. **In your own words, say what you think the speaker said.**    - Occasionally preface with a comment like: “It sounds like you’re saying…did I get it?” |
| Make vague, awkward, or incomplete statements | - Help the speaker **relax** by **drawing him/her out with open-ended questions:**   - **You can say**: “Can you say more about that?” *or* “What do you mean by…?” *or* “What matters to you about that?” |
| Hesitate to contribute | - Don’t quickly press the issue, but instead look for an opportunity to **direct a question to the whole group**:   - **You can say:** “Are there other ways of looking at this?”   - Or, “Does anyone have a different point of view?” - There’s also opportunity to **direct a question to this person**:   - **You can say:** “What do you think of that approach/idea?” *or* “Do you have anything to add?” |
| Go off on a tangent | - Ask the person to help everyone see **how his/her point connects with the broader context**. - **You can say:** “How does your idea link up with [our topic]?”   - Validate the explanation: “Are you saying…[paraphrase]?”   - Then, follow-up with, “Okay, we have [name’s] idea. Whose turn is it to go next?” - You can also redirect by clarifying the objectives for the discussion. |
| Seem to have exhausted their contributions | - If you think the group has generated lots of good discussion already, then it’s fine to end. - If there are open questions or something needs clarity, you can use that to ask open-ended questions to elaborate more on an idea. - If you see a “gap” in ideas, ask the group for their opinion on that gap (e.g., “We haven’t talked about what doctors can do…”) |
| Come to the end of a part of the discussion | - Summarize ideas/decisions. |
| Are silent | - Tolerate silence if they seem to be gathering thoughts (look for non-verbal indicators of readiness to speak).   - You can count to 5 or 10 slowly before prompting for input. Especially at the beginning of the day, silence may be longer and more likely as people gather their thoughts and hesitate to be the first to speak up. - If simply quiet on subject, ask probing questions:   - **You can say:** “I’d like to get opinions from those who haven’t talked for a while.”   - Or, “Any new thoughts? [participant], you look as if you might be about to say something…” - You can also ask someone to restate the objective of the discussion. |
| Exhibit guarded expressions | - Make inquiry and probe. - **You can say:** “Looks like you’re having a reaction to that. I’m guessing you’re [emotion]. Am I close?” |
| Emotions are running “high” | - Acknowledge the emotion - **If it’s on task, you can ask probing questions:** - **You can say**: “You sound a bit worried. Can you say more about what you are thinking?”   - Then paraphrase the content of the thought and redirect to task. |
| Emotions are running “low” | - Invite expression of emotion with opinions. |
| Dispute/oppose/attack with loaded questions | - Turn loaded questions into problems to be addressed by group. |

**DVR Assignments**

**Instructions**

How to turn on DVR

Turn DVR over.

Slide the top bar from OFF to ON.

1. When ready to record, move the side bar to REC (a red light will flash on the top of the device to indicate it’s recording).
2. **At the beginning of each recording please say your group name and name of the activity (example: DM, Who-Do: Selecting the Most Valuable activity)**
3. When you’ve finished recording, move the side bar to STOP (the red light will turn off).

# How to turn off DVR

1. Make sure the side bar is set to STOP.
2. Move top bar from ON to OFF.

# Note: Please be sure to STOP recording and turn OFF recorder. Each time you turn ON recorder a new audio file is created.

**A. Overview and Agenda Presentation**

Time: 4:05-4:15pm (10 minutes)

## Materials

- PowerPoint presentation

## Facilitate Activity

No instruction.

**B. Team Introduction & Icebreaker**

Small group introductions and ice breaker activity.

## Time: 4:15-4:40pm (25 minutes: 20 minutes for discussion and 5 minutes to read case)

## Materials

- Phase 1 cases
- De-intensification cards

## Facilitate Activity

1. Start the discussion by explaining your group’s de-intensification recommendation (in 1-2 sentences).
   - *Experts are recommending the following:* ***[state your group’s recommendation].***
2. **Read your Phase 1 case** as a group. You should read the background and ask for a volunteer to narrate the patient and provider perspectives.
   - *To illustrate the recommendation to [re-state group’s recommendation], we thought it would be helpful to share a hypothetical de-intensification story from the perspective of a patient and his/her provider.*
3. After reading the cases, **ask each person to take 1-2 minutes to share a personal experience about scaling back** (from the cards they filled out prior to the forum). You can start the discussion by sharing a story of your own de-intensification experience.

- *While we’re doing these activities, we’re going to use first names to keep things casual.*
- *You were sent a card with a question that asked about your personal experience with de-intensifying. We’re interested in hearing more about your experience. [Patient’s name], can you describe a time when: 1) you went to your doctor wanting a specific test; 2) your doctor persuaded you that NOT getting the test was the best thing to do; and, 3) in the end, you felt good about it.*
- *[Doctor’s name]:*
  - Think about times when **decreasing or stopping a diabetes medication** (or some other medication) was the best option for a patient and when you discussed it with them, they agreed that decreasing or stopping was indeed in their best interest. Then, describe a few ways that patients have made these “de-intensification” conversations easier for you in the past.
  - Think about times when **stopping or decreasing the frequency of colorectal cancer screenings** (or some other cancer screening) was the best option for a patient and when you discussed it with them, they agreed that decreasing or stopping was indeed in their best interest. Then, describe a few ways that patients have made these “de-intensification” conversations easier for you in the past.
  - Think about times when a patient came to the clinic wanting a **carotid artery ultrasound** (or some other testing/treatment that isn’t recommended), but after some discussion you were able to persuade the patient that it really wasn’t in their best interest to have the test. Then, describe a few ways that patients have made these conversations easier for you in the past.

1. **(Optional)**. During the discussion, you can note key facts, emotions and challenges people experienced in the space below.
2. **(Optional).** Ask 1 or 2 participants that have especially interesting and relevant stories to go more in-depth about their experiences.
3. Wrap up the discussion.
   - *Thank you for sharing your de-intensification stories with the group today. I think that your personal stories as well as the cases we read really connect to our group’s de-intensification recommendation to stop/scale back [topic].*
4. Collect cards from participants.

**Notes**

________________________________________________________________________________________________________________________________________________________________________________________________________________________________________________________________________________________________________________________________________________________________________________________________________________________________________________________________________________________________________________________________________________________________________________________________________________________________________________________________________________________________________

**C. Review Super Strategies and Discuss the Goal of Who-Do**

In small groups, patients are re-introduced to the strategies they developed in Phase 1, and providers learn more about how the strategies were developed.

## Time: 4:40-4:50pm (10 minutes)

## Materials

- Super-super strategy table

## Facilitate Activity

*During our Phase 1 forums, VA patients developed high-level solutions for de-intensification. We’ve grouped these into strategies that happen during an encounter and outside an encounter. We will spend a few minutes reviewing these strategies.*

1. **Reintroduce your group’s super strategies**. (These will be mentioned during the intro presentation.) Ask your group to review the table silently and jot down any questions.
   - **For CRC Team:** *We’re going to focus on the strategies that happen during a patient visit.*
   - **For CAS/DM Team:** W*e’re going to focus on the strategies that happen outside of a patient visit.*
2. After a few minutes, ask if anyone has questions and summarize your group’s strategies.
3. Review the **goal of Who-Do**. Take temperature of the group and reinforce ideas as needed from introduction presentation.

*We’re here because we want to come up with good strategies to get people to de-intensify when it’s appropriate. For patients, you know what it’s like to go through the health system. For doctors, you know the inner workings of clinics, so you know what happens behind the scenes.*

- - **For CRC Team:** *In the next activity, you will work together to brainstorm concrete actions that the VA can take* ***during an appointment*** *to support appropriate de-intensification. As you’re working through the next activity, try to think of concrete ways to:*
    - **Refer to “Need ways to” in super strategies table**
  - **For CAS/DM Team:** *In the next activity, you will work together to brainstorm concrete actions that the VA can take* ***outside of an appointment*** *to support appropriate de-intensification. As you’re working through the next activity, try to think of concrete ways to:*
    - **Refer to “Need ways to” in super strategies table**

**D. Who-Do: Brainstorm Who-Do’s, Select Most Important Who-Do’s, and Brainstorm Barriers and Ways to Overcome**

Who do you want to do what? Small groups identify the people and actions, as well as solutions to overcoming potential obstacles, to ensure that the super strategy leads to successful de-intensification.

## Time: 4:50-6:20 (90 minutes: 30 minutes for Who/Do brainstorm, and 60 minutes for brainstorming barriers and how to overcome solutions for each selected Who/Do)

## Materials

- Large sheets of paper
- Post-it notes
- Pens/markers
- Strategy table
- Container + fishbowl slips of paper (for participants)
- Fishbowl sheets with long and short wording (for facilitators)
- Audio recorder

**Reminders to Facilitators:**

- It’s okay if your group does not fill in all the cells!

**Set-Up**

- Each matrix represents a unique idea at different levels of the healthcare system: National, Local (hospital or clinic), and Patient/Primary Care Team levels. Across each level, we would like participants to consider the following:
  - **Who**: ‘*Who’ is involved in making this happen? Who is the decision maker? Who has needed resources? Who may be an obstacle? Whose support is needed? (can be individuals or groups).*
  - **Do**: *What do they need to do, or do differently? What actions will build toward the big goal? (each DO should be concrete and measurable)*
  - **Barrier**: *What could get in the way of the ‘Do’ occurring? What potential problems exist?*
  - **How**: *What needs to happen to be able to overcome that barrier?*
- To ensure that all super strategies are covered:
  - **CAS Team:** start with the patient education through outreach + educate patients through mass/social media super strategies
  - **DM Team:** start with provider education super strategies
  - **CRC Team:** start with any super strategy

## *
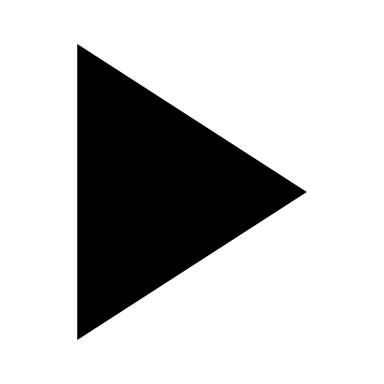
*Facilitate Activity

**Start Recording**

**Brainstorm Who/Do’s for ALL Super Strategies (4:50-5:20pm: 30 minutes)**

1. **Write the big goa**l on a large piece of paper or the whiteboard (keep this visible for the duration of the activity).

**Goal: To brainstorm concrete actions that would support appropriate de-intensification.**

1. **Demo the activity:** starting at any level, go through a full Who-Do row with the group (including a barrier and ways to overcome). At this point you can mention the other levels you’ll discuss later. Remember to just focus on the first Who/Do cell for this activity.
   - *We just talked about the [name] level, but there are others to consider. Today, we’ll talk about actions/decisions at three distinct levels. In a way, each level can work to support each other. For example, the national level can support our efforts at the patient/provider level. We’ll go into more depth about this later. We’re just going to focus on the first Who/Do cell for this part of the activity.*
2. Instruct participants to write Who/Do ideas on sticky notes based on the discussion about the group’s super strategies. You do NOT need to indicate the super strategy category for each Who/Do. **Participants will have 30 minutes total to brainstorm Who’s and Do’s**. There are a few ways you can approach this:
   - **Option 1 (structured discussion)**: **Give participants 10 minutes to brainstorm Who/Do’s for each super strategy**. Instruct participants to take 1-2 minutes writing their ideas on sticky notes and placing in front of them when finished. Ask participants to read their ideas out loud. Spend remaining time brainstorming additional Who-Do’s. **Repeat for the next 2 super strategies.** To guide the discussion, you can say something like:
     - *What can we do in this area [super strategy] to support de-intensification?*
     - *What can be done and who can/should do it?*
   - **Option 2 (open discussion)**: **Allow participants to start the discussion without a specific super strategy in mind.** You can take a group discussion-based approach—participants can write down ideas on sticky notes while group discusses (although it’s likely the facilitator will also need to capture ideas as they emerge).
     - If you notice that 1 or more super strategies haven’t been covered by a certain point, you can redirect the group to a specific super strategy.
     - *What can we do in this area [super strategy] to support de-intensification?*
     - *What can be done and who can/should do it?*
   - Facilitators should also give participants a handout to help them identify professional positions at various levels.
3. After the discussion, place 1 large sheet of paper with the matrix template on the wall/whiteboard.

**Select Most Important Who-Do’s/Actions (5:20pm-5:30pm: 10 minutes)**

1. As a group, s**elect the 3 most important Who/Do’s** that will best assist with de-intensification. Ask patients first then providers. You don’t need to select a Who/Do from each super strategy category.
   - *As a group, you’re going to pick out your 3 most important Who/Do’s.*
     - *Which ‘Who/Do’ do you think would be most effective in making sure there’s appropriate de-intensification? What are the 3 best ideas?*
     - Then, once the 3 Who/Do’s are identified: *At what level do you think this Who/Do fits?*

*2)* Then ask the group to decide which of the 3 Who-Do’s they would like to use to start their matrix.

- *Which Who-Do would you like to start with first?*

3) Write the starter Who-Do directly in the cell for the best corresponding level (unless specified by the group earlier). Mark the starting point by writing **START** directly above.

**Brainstorm Barriers and Ways to Overcome (5:30pm-6:20pm: 50 minutes)**

1. Prompt your group to brainstorm any **potential barriers that could stop the Who/Do (action)** from happening. Write barriers directly in the cell as they’re mentioned. (**Note**: we want to steer people away from barriers that have a one-time solution…want to focus on repeated decisions or actions). For example:
   - *Why wouldn’t this [Do] be* ***effective****?* Or *What are barriers to that [Who and Do] being* ***effective****?*
   - *What is a barrier to getting this [Do]* ***done****?* Or *what are barriers to putting this [Do] in place?* Or *What is a barrier to using this [Do] to achieve de-intensification?*
   - If a one-time barrier does come up, just say: *I’m going to put this in the parking lot. While this is an important solution to this barrier, it is outside of our control and may not be possible.*
2. Once the group has brainstormed all possible barriers, instruct participants to **determine what they feel is the most important barrier to solve.** **Circle the most important barrier FOR EACH ROW.**
   - It’s possible to have more than 1 barrier if there is 1 solution that could solve each barrier (see # 1 below for facilitator prompt).
   - *Let’s take a look at the barriers you just brainstormed. Out of all the ideas in this cell, what’s the biggest barrier (or the big thing that could get in the way of this [Do] being effective to get people to de-intensify? Why?)*
3. Move to the “How to Overcome” cell. Instruct the group to **discuss ways to overcome** the biggest barrier for this Who-Do. Ask the group to choose the best “How to Overcome” idea and write this in the cell.
   - ***What’s the best way to overcome the most important barrier in this cell?*** *Is there anything that can overcome more than 1 barrier?* Focus on repeated processes/actions, not on 1-time things.
4. If participants are struggling to come up with ideas for ways to overcome the biggest barrier, they can select a piece of paper from the **Fishbowl** to help generate ideas. Participants can select as many Fishbowl ideas as they’d like.

| **Original Wording (for facilitators)** | **Shortened Wording (for participants)** |
| --- | --- |
| Provide a summary to clinicians of their own prescribing habits compared with their peers | Compare clinicians prescribing habits to those of their peers |
| Provide a reward (e.g. financial bonus, promotion) to clinicians for stopping or reducing when appropriate | Incentives and disincentives (e.g. financial, promotion) |
| Report hospital performance on stopping and reducing to the public | Report hospital performance to the public |
| Have prescription or screening requests, initiated by a clinician, reviewed and approved by a pharmacist or specialist | Administrative review before all prescriptions and screening requests are approved |
| Hand out educational materials about stopping or reducing to patients | Educational materials |
| Provide training to clinicians on how to talk with patients about stopping or reducing | Communication skills training |
| Provide clinicians with decision-making (about stopping or reducing) tools to help during a medical appointment | Decision support tools |
| Have national experts create guidelines for clinicians on who and when to stop or reduce | National expert guidelines |
| For example, changing the stake holders that are making the decisions (i.e. more clinicians or more patients) | Change in the policy making process |
| Designing information or technology that could support people (i.e. doctors, patients, leadership) | Health IT/Technology |
|  | Media/Advertising |

1. Once your group has come up with their idea for overcoming the most important barrier, they can **move to the next level**. Depending on what level the discussion ended, you can follow up by asking how an action can be supported at one of the other levels. For example:
   - If someone has an idea starting at the patient/provider level, you will want to follow up with *how* that idea can be supported at the local level (as well as national level) to help support de-intensification.
   - If someone has an idea starting at the national level (as well as local level), you will want to follow up with how that idea trickles down to influence the other levels.
     - *To support this [overcome],* ***what needs to happen at this level to make sure [the overcome] happens****? To start, what can be done and who can/should do it?*
     - *How do you make sure it happens at the [patient/provider/local/national] level?*
     - For every [doctor-patient/local/national level] idea, what are the supporting things that need to happen at the other level(s)?”

**REPEAT BRAINSTORM BARRIERS AND WAYS TO OVERCOME FOR THE 2 OTHER MOST IMPORTANT WHO-Dos**

**Stop Recording**


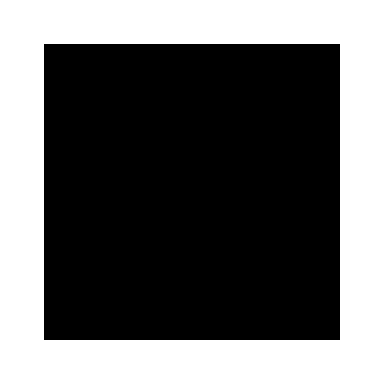


**DINNER BREAK (6:20pm-6:40pm)**

**E. Brief Reminders**

Provider instructions and rationale for the Who-Do: Selecting the Most Valuable Activity and Who-Do: Refinement activities.

## Time: 6:40pm-6:50pm (10 minutes)

## Material

- PowerPoint Presentation

## Facilitate Activity

- No instruction.

**F. Who-Do: Selecting the Most Valuable Activities**

This will be a continuation of our working dinner. Each group will try to determine 3 specific activities or decisions at the LOCAL or NATIONAL levels that potentially supports (or leads to) appropriate de-intensification. Pinpointing the most valuable activities will help us determine where simple rules will have the greatest impact.

## Time: 6:50pm-7:00pm (10 minutes)

## Material

- Completed Who-Do matrices
- Audio recorder

## Reminder to Facilitators

- Depending on time, we may not get to the other two matrices when selecting the most valuable activity.

## Facilitate Activity

**Start Recording**

*
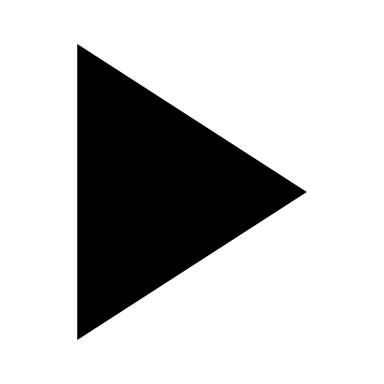
*

1. Ask participants to **pick what they think are the 3 most “valuable” activities in the Who/Do column at the LOCAL and NATIONAL levels (from all matrices)**. **Order the activities from most valuable (1^st^), runner up (2^nd^), and 3^rd^.**
   - *Let’s take a look at the matrices you just created. We’ll focus on the activities and people involved at the local and national levels. What 3 activities do you feel are the most “valuable?” Which activities do you think:*
     - *Is most likely to* ***lead*** *to appropriate de-intensification?*
     - *Is most* ***sustainable****?*
     - *Is most* ***acceptable*** *to patients, staff, providers, specialists, etc.?*
   - *What do others think? Do you agree or disagree? Why or why not?*
2. **Circle the most valuable activities**. You can also place a notecard or sticky note with “Most Valuable Activity” printed on it next to the activity.

**G. Who-Do: Refinement**

Each group will have the opportunity to delve deeper into the most valuable activities identified earlier. The detailed information from this activity will help with the development of specific simple rules.

## Time: 7:00pm-7:50pm (50 minutes)

## Material

- Matrices with the most valuable activities identified by the group
- Who-Do refinement matrix

## Facilitate Activity

*Now we’re going to take a closer look at [one of the most valuable activities]. We’ll start by discussing ways to overcome [the biggest barrier] to help accomplish [one of the most valuable activities], and brainstorm the steps required to make the “ways to overcome” happen.*

1. Place a blank Who-Do refinement matrix (see pg. 20) next to one of the completed Who-Do matrices (with one of the most valuable activities circled).
2. **Write the biggest barrier (identified earlier) in the matrix.**
   - *Let’s start by focusing on the biggest barrier you identified earlier. You said that the biggest barrier to doing [one of the most valuable activities] was [biggest barrier].*
3. Confirm agreement on the top “way to overcome” idea selected earlier to start the refinement matrix. **Write the top “way to overcome” idea in box 1A.**
   - Earlier, you suggested ways to overcome [biggest barrier] as [x,y,z…]… do you still feel like [x] is the best way to overcome this barrier to accomplish [one of the most valuable activities]?
4. Ask if there are other ideas to consider that were not part of the original list. Add columns below 1A for new “ways to overcome” ideas (please mark as 2b, 3c, etc.).
   - Are there other things that can be done to overcome [biggest barrier] that we haven’t thought of already?
5. **IF** participants come up with new ideas: ask **if there are any ideas in particular that they would like to talk about more. Circle this activity.**
   - *Which activity stands out to you the most? Why?*
6. Ask participants **to brainstorm the steps required to make the “way to overcome” idea circled earlier happen.**
   - *Let’s talk through how we could implement/do this:*
     - *What do you think are the steps that need to be done to do [1A, 2B, 3C…]?* OR *how does [1A, 2B, 3C…] get done? What does that look like?*
     - *How do you make that happen on a regular basis?*
     - *How can we (health systems) do a better job at this process?*
     - *What are your experiences with doing something like this?*
     - *How would this work?*
     - *How would we implement this?*
7. Ask participants to **identify the step that is hardest to do/accomplish**. Call the floating facilitators to the table to confirm that the hardest step is a **recurrent process**. **Circle this step and mark “biggest bottleneck.”**
   - *Of all the things that need to be done to do [1A, B, C…], which step is the hardest to do/accomplish?*
8. Ask your group to discuss **ways to improve the hardest step, including who’s involved in that process. It’s important to understand their rationale.**
   - *It looks like there may be room for improvement. Any ideas for how this step could be improved?*
   - *Who would we want to talk to that might be able to help us improve the process? Why?*

**Who-Do Refinement Matrix Template**

| **BIGGEST BARRIER: Facilitators write this in** | |
| --- | --- |
| **Ways to OVERCOME THE BIGGEST BARRIER to ACCOMPLISH WHO DO (activity):** | **Steps required to MAKE WAYS TO OVERCOME HAPPEN (*activities, decisions, actions that need to be done on a regular basis)*** |
| 1a. [Facilitators write in from one of the most valuable activities] |  |

**Stop Recording**


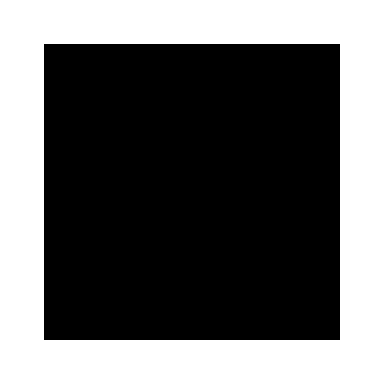


**H. Thank you, post-survey, and distribution of gift cards to patients**

## Time: 7:50pm-8:00pm (5-10 minutes)

## Material

- Post-surveys
- Gift cards

## Facilitate Activity

No instruction.

**1. Example Who-Do Matrix**

| Level | Who/Do | Barrier (or rename) | How to Overcome |
| --- | --- | --- | --- |
| Patient/Primary Care Team Level | None! | Uninteresting | Don’t care |
| Local (hospital or clinic) Level | **START**  **Who:** Office manager  **Do:** Ensure that the clinic is stocked with informational brochures on the newest recommendations to de-intensify colonoscopy screenings | No one looks at all the brochures  The cost is too high to make all the brochures | Only 1-3 brochures available at a time, office manager should prioritize which ones should be out there.    **(start Who-Do at National level next)** |
| National Level | National decision-making body should determine which brochures should be prioritized (office of health promotion and disease prevention), guideline committee | Locally they do not pay attention to the guidelines anyway | Incentivize the clinics to display the brochures by giving effort to health educator to restock the brochures |

**2. Identify biggest barriers**

| Level | Who/Do | Barrier (or rename) | How to Overcome |
| --- | --- | --- | --- |
| Patient/Primary Care Team Level | None! | Uninteresting | Don’t care |
| Local (hospital or clinic) Level | **START**  **Who:** Office manager  **Do:** Ensure that the clinic is stocked with informational brochures on the newest recommendations to de-intensify colonoscopy screenings | No one looks at all the brochures  The cost is too high to make all the brochures | Only make 1-3 brochures available at a time, office manager should prioritize which ones should be out there. |
| National Level | National decision-making body should determine which brochures should be prioritized (office of health promotion and disease prevention), guideline committee | Locally they do not pay attention to the guidelines anyway | Incentivize the clinics to display the brochures by giving effort to health educator to restock the brochures |

**3. Identify most valuable Who/Do at either the LOCAL and NATIONAL levels**

| Level | Who/Do | Barrier (or rename) | How to Overcome |
| --- | --- | --- | --- |
| Patient/Primary Care Team Level | None! | Uninteresting | Don’t care |
| Local (hospital or clinic) Level | **START**  **Who:** Office manager  **Do:** Ensure that the clinic is stocked with informational brochures on the newest recommendations to de-intensify colonoscopy screenings | No one looks at all the brochures  The cost is too high to make all the brochures | Only make 1-3 brochures available at a time, office manager should prioritize which ones should be out there. |
| National Level | National decision-making body should determine which brochures should be prioritized (office of health promotion and disease prevention), guideline committee | Locally they do not pay attention to the guidelines anyway | Incentivize the clinics to display the brochures by giving effort to health educator to restock the brochures |

**4. Who-Do Refinement**

| **BIGGEST BARRIER:**  Locally they do not pay attention to the guidelines anyway | |
| --- | --- |
| **Ways to OVERCOME THE BIGGEST BARRIER to ACCOMPLISH WHO DO (activity):** | **Steps required to MAKE WAYS TO OVERCOME HAPPEN (*activities, decisions, actions that need to be done on a regular basis)*** |
| 1a. Incentivize the clinics to display the brochures by giving effort to health educator to restock the brochures | Step 1  Step 2 --Biggest bottleneck  Step 3  Step 4  Step 5 |
| **ADD ROWS IF THERE ARE NEW IDEAS FOR WAYS TO OVERCOME** | |
| 2b. [NEW WAY to overcome biggest barrier to ACCOMPLISH WHO DO) |  |
| 3c. [NEW WAY to overcome biggest barrier to ACCOMPLISH WHO DO) |  |
| 4d. [NEW way to overcome biggest barrier to ACCOMPLISH WHO DO) |  |
| 5e. [NEW way to overcome biggest barrier to ACCOMPLISH WHO DO) |  |

| Level | Who/Do | Barrier (or rename) | How to Overcome |
| --- | --- | --- | --- |
| Patient/Primary Care Team Level | None! | Uninteresting | Don’t care |
| Local (hospital or clinic) Level | **START**  **Who:** Office manager  **Do:** Ensure that the clinic is stocked with informational brochures on the newest recommendations to de-intensify colonoscopy screenings | No one looks at all the brochures  The cost is too high to make all the brochures | Only make 1-3 brochures available at a time, office manager should prioritize which ones should be out there. |
| National Level | National decision-making body should determine which brochures should be prioritized (office of health promotion and disease prevention), guideline committee  **One of the Most Valuable Activities** | Locally they do not pay attention to the guidelines anyway  **Biggest Barrier** | Incentivize the clinics to display the brochures by giving effort to health educator to restock the brochures |
