# Supplementary material for: Developing Strategies to Reduce Unnecessary Services in Primary Care: Protocol for User-Centered Design Charrettes
Source: JMIR Res Protoc. 2019 Nov 26;8(11):e15618. doi: 10.2196/15618 (PMC6904896; doi:10.2196/15618)
Supplement: Multimedia Appendix 10 [file resprot_v8i11e15618_app10.pdf]

SUMMARY STATEMENT

PROGRAM CONTACT: (Privileged Communication) *Release Date:* 09/30/2015

Application Number: 1 I01 HX001895-01A1

Principal Investigators (Listed Alphabetically):
HOFER, TIMOTHY P. MD
KERR, EVE A MD (Contact)

Applicant Organization: VETERANS HEALTH ADMINISTRATION

Review Group: HS5A
Health Care System Organization and Delivery
Health Care System Organization and Delivery - Special Panel

Meeting Date: 08/25/2015 *RFA/PA:* HX15-023
Council: OCT 2015
Requested Start: 01/01/2016

Project Title: Identifying, Measuring, and Facilitating Opportunities for De-intensification of Medical Services
SRG Action: Impact Score: 149

Human Subjects: 30-Human subjects involved - Certified, no SRG concerns
Animal Subjects: 10-No live vertebrate animals involved for competing appl.
Gender: 1A-Both genders, scientifically acceptable
Minority: 1A-Minorities and non-minorities, scientifically acceptable
Children: 1A-Both Children and Adults, scientifically acceptable
Clinical Research - not NIH-defined Phase III Trial

Project Year	Direct Costs Requested
1	430,697
2	354,763
3	387,525
4	126,866
TOTAL	1,299,851

ADMINISTRATIVE BUDGET NOTE: The budget shown is the requested budget and has not been adjusted to reflect any recommendations made by reviewers. If an award is planned, the costs will be calculated by VA Office of Research and Development (ORD) staff based on the recommendations outlined in the BUDGET COMMENT section and any relevant ORD service-specific limitations.

NOTE TO APPLICANT: No funding decision has been made on this proposal at this time. If a decision is made to fund this proposal, you will be notified to complete the Just-in-Time process at your local VA Medical Center as soon as possible so that there will be no delay in the release of funds. As a reminder, funding is always dependent upon the availability of funds.

KEY SUMMARY POINTS:

- The area of focus is on de-intensification in primary care is of high concern to the VA. Strong investigative team with excellent partnership with VA clinical leaders
- Very responsive to the RFA and to prior critiques. This is a high-risk and high reward study.
- They have provided many details on which measures of treatment will be operationalized. Multiple-PI is justified due to the distinct scope of work outlined.
- Comment: Sample size for some of the proposed measures may be problematic, especially in terms of evaluating individual providers. However the reviewers acknowledge that it's hard to do a power calculation because the measures are not yet firm

DESCRIPTION (provided by applicant):

Providing appropriate health care means ensuring that patients get the care they need while avoiding care that is unnecessary or harmful. In the 1990s, VHA's first transformation focused on increasing the use of necessary services through a performance management system that has become a panoply of guidelines, performance measures, reminders, registries, and incentives. As a result, the underuse of necessary services in VHA decreased dramatically. Today, there is growing interest in identifying overuse - care that exposes patients to services that are not beneficial or may cause harm and which may take scarce resources away from those who would benefit from them. The national Choosing Wisely campaign is one manifestation of this trend. While this campaign has garnered attention, it has not produced systematic or reproducible approaches to identify overuse. Within the general realm of overuse, a more focused target is promoting de-intensification when good quality practice calls for decreasing the intensity or frequency of medical services that are currently part of a patient's ongoing management. Increasingly, we recognize that care is too frequent or too intensive in cases where the marginal benefit is absent or there is potential for patient harm, and that both providers and patients have particular difficulty stopping routine testing and treatments once they have successfully become part of a patient's regular care. One example is intensive glycemic management among diabetic patients who are elderly or have major comorbidities. Yet, most quality monitoring and improvement initiatives continue to provide largely unopposed incentives to escalate care intensity. The proposed study will: 1) identify and validate clinical indications for de-intensification in primary care (Aim 1); 2) assess the prevalence and reliability of measures of de-intensification in VHA (Aim 2); and 3) develop multi-level strategies to disseminate and implement de-intensification measures (Aim 3). Aim 1 will involve an environmental scan to identify a preliminary list of potential de-intensification indications; a rapid evidence synthesis of existing evidence supporting candidate de-intensification indications; a high level prevalence estimate of opportunities for de-intensification in VHA; and, expert panels to rate each of the potential indications on improvement opportunity, validity, and feasibility of measurement and implementation. Aim 2 will include constructing measures of de-intensification indications and conducting an analysis to examine prevalence, variance, and reliability of measuring de-intensification in VHA. AIM 3 will involve engaging providers, clinical managers, and patients in collaborative decision-making to develop an operational measurement procedure for disseminating and implementing the identified de-intensification measures. Findings from each of the Aims will be synthesized into practical intervention strategies. An advisory council, comprised of VHA and national policy, content and measurement experts, will work with the research team in all stages to review results and provide guidance on indication measurement prioritization, feasibility, implementation, and dissemination. Identifying, measuring, and understanding how to promote de-intensification - to complement the many measures promoting appropriate intensification - is critical to restoring balance to VHA's efforts to improve care quality. This study will develop a systematic method to identify when de-intensification is called for, assess how frequently de-intensification occurs in VHA, and provide approaches to reliably measure and deploy measures of de-intensification. The project is unique by involving all stakeholders, including clinical and policy experts, front-line providers, clinical leaders, and patients in developing an operational measurement procedure and implementation approach that can ensure reliable measurement, promote quality improvement, and avoid organizational dysfunction.

CRITIQUE 1

1. Responsiveness to the Priority Areas Outlined in this RFA.

The proposal fits squarely within the goals of the RFA; namely, to develop novel measures that address overuse of low-value health care services. The proposal also characterizes sources of variation in the proposed measures, which is another goal of the RFA. However, the RFA also states: “It is expected that this RFA would fund initiatives that develop innovative approaches through use of inter-disciplinary research teams comprised of expertise in computer science, statistics, engineering, mathematics, clinical epidemiology/decision-making, social sciences, and health economics with the aim to turn existing data into knowledge for improved healthcare and health.” I do not see many of these disciplines other than a biostatistician and clinical psychologist, the latter at 7.5% effort.

2. Approach.

The approach is systematic and well-conceptualized. Strengths include the use of an evidence synthesis, involved operational partners, a modified Delphi process, and extensive patient and provider engagement.

One main concern with the approach is whether it will generate a substantial number of valid and important performance measures. Will these be a large enough denominator for overuse measures to assess facility, practice and provider variation? This has been a challenge with developing such measures in the past. For instance, while a measure of underuse of colorectal screening would apply to a large population of most adults age 50 -79, the measure of overuse would apply to a much more selected population of adults with limited life expectancy. Presumably the variation across sites would be much lower for such a measure. Many of the Choosing Wisely measures apply to very small and highly selected populations – which may not yield the necessary sample size for reliable reporting at all the different levels proposed in this study. Power and sample size calculations for a few candidate measures (even if these are not ultimately selected for prioritization in the study) would greatly enhance the confidence that this project will be actionable for measurement and reporting in the VA. Based on the analyses reported in Appendix 1, the diabetes measure is likely to have sufficient sample size, but to this reviewer, it is unclear how many additional indicators of overuse will be valid for assessment at the provider, practice, or even VAMC level.

It is unclear how the proposed examples of overuse on page 27 (Table 2) could be developed into performance indicators. For example, the USPTF recommends a shared decision-making approach regarding PSA testing. How would this be operationalized into a performance metric? Some of the other examples – lipid testing, frequency of a1c testing – involve tests with extremely low costs; or have equivocal evidence about when to discontinue screening. Again, this raises concerns about just how many meaningful measures of de-intensification will arise from this study.

3. Innovation and impact.

There are a number of features in this proposal that are innovative. Most notably, the development of a comprehensive set of measures to assess de-intensification and profile variation across facilities has not been done. Although focused in the VA, the project would have impact in other health systems given the recent policy focus on developing measure of overuse and the Choosing Wisely campaign. The advisory board of VA operational leaders and representative from KP will also enhance the potential impact of the project.

4. Investigator Qualifications; Facilities and Resources.

The PIs are exceptionally well-qualified to carry out the proposed research. Also, this work builds on the previous work of the PIs and the Ann Arbor COIN. My one concern is that there are 6-7 co-investigators all funded in the 5-10% range, several of whom appear to have overlapping skill sets. It is not clear why each of these individuals are involved and what all of their specific role on the project will be. This could lead to a diffusion of responsibility.

5. Leadership Plan.

The multiple PI plan seems appropriate given the discrete set of tasks each PI will oversee. The two PIs have an extensive track record of collaboration

6. Adequacy of Response to Previous Feedback Provided by HSR&D Regarding the Proposed Study.

The authors thoroughly responded to the prior critiques from the previous review.

7. Protection of Human Subjects from Research Risk.

I see no concerns

8. Inclusion of Women and Minorities in Research.

Appropriate; the investigators will use random selection, but they may want to consider oversampling women and minority patients if the participation rates are low.

9. Budget.

This project has a very large budget beyond the typical limit for an IIR. However, there is a large scope of work.

10. Overall Impression.

This is an innovative project that addresses an significant issue for VA and other health care systems. The approach seems likely to have an impact given the involvement of patient and provider stakeholders. The enthusiasm is somewhat diminished by concern that there will be a large number of measures with adequate sample size to profile meaningful variation across providers and facilities.

11. Key Strengths.

1. Area of high concern to VA and other health systems

2. Strong investigative team

3. Excellent partnership with VA clinical leaders

4. Engagement of front-line providers

12. Key Weaknesses.

1. Some concerns remain about the feasibility of developing valid and important indicators that have sufficient sample size to characterize variation across all of the units of analysis

CRITIQUE 2

1. Significance (including Importance of the Problem Addressed).

The goal of de-intensifying care when appropriate is very current and has achieved broad consensus as a priority, especially within VA. In large part due to work by the applicants and others, we now have pretty clear evidence that our pursuit of control targets and quality measures has the unintended consequences of overtreatment for a fairly large number of our patients. VA is already working on several initiatives to address de-intensifying care when appropriate, such as the hypoglycemia safety initiative. In this proposal, the applicants plan to do foundational/conceptual work to better understand the context of de-intensification and also how such efforts may translate into performance measures. They make the point, correctly, that we have focused so single-mindedly on doing more for patients that little is known or understood about how we would even go about doing less. While VA can and should proceed with efforts to promote de-intensification when appropriate, it seems like a good idea to have this sort of foundational work going on as well. To use somewhat casual language, while firing from the hip may hit the target, if it does not, VA would do well to have funded a group to already be looking into where exactly we should be aiming in the first place.

2. Approach (including Feasibility).

There are 3 aims 1) to identify and validate indications for de-intensification 2) to assess the prevalence and reliability of measures of de-intensification, and 3) to develop strategies to implement de-intensification measures. Aim 1 will include a “scan” to locate all likely candidate targets for de-intensification, rapid evidence synthesis about each, and a modified Delphi panel to help prioritize the targets with the highest evidence support and perceived importance. The use of ThinkTank to help facilitate meaningful conversations while avoiding the hassles of VA travel makes sense. Aim 2 will include efforts to actually specify performance measures based on the Aim 1 work, including comparing to a gold standard of EMR review and routine measures of prevalence, variance, and reliability. This is important work to understand how such measures will work in practice before deploying them. In Aim 3, the investigators will pivot to planning for effective implementation of the measures, incorporating views gathered from patients, VA leaders, and clinicians to help inform the effort.

One important critique which I have of the general direction of this research effort. The investigators seem to imagine, above all, that the VA needs opposing sets of measures – such as patients with too high a blood glucose, and patients with too low a blood glucose. This can work OK, and is logical. But I wonder if VA needs twice as many performance measures as we have now. It may also add to a sense among already-beleaguered clinicians that they cannot win, as they face “failure” on either side (too high/too low). While I do not have an obvious answer, I wonder if opposing sets of measures is really the best approach to take. Isn’t there a way to produce fewer, more integrated measures? I realize this risks eroding the actionability of the measure, if you can’t easily tell which patients are too high vs. too low, for instance. But maybe there is a better way than just doubling the number of measures we have.

3. Impact and Innovation.

The proposal details a plan for careful and at times heavily conceptually-informed and foundational work. While HSR&D has a growing demand to always produce things quickly, sometimes careful work takes time to do. The investigators do make the point that they will make sure to work on a few high priority measures early (as assessed by their advisory panel of VA leaders), to have these measures ready for roll-out in 2.5 years if possible. The proposal is not “innovative” in the sense that well-

established methods will be used. However, these methods have not been systematically applied to this important question, and if VA does not invest in such an effort now, they may regret it in 3 years when they are begging for the kind of information this project is likely to deliver. As our recent experience with performance metrics can attest, deploying performance metrics without careful forethought can have unforeseen consequences for our VA system.

4. Investigator Qualifications, and Facilities and Resources.

As was stated in the previous review, this group of researchers has a superb track record doing just this kind of research, of working together, and of producing really high-quality products. The facilities and resources are well-matched to the goals of the project. I have a high degree of confidence that they can successfully complete this work.

5. Multiple PI Leadership Plan.

The multiple PI plan between Drs. Kerr and Hofer is appropriate and credible.

6. Adequacy of Response to Previous Feedback Provided by HSR&D Regarding the Proposed Study.

The investigators have been responsive to the critiques. They appropriately grasped the major underlying issues which were raised and have addressed them directly. There are limits to how much they can change the project, but they have made small moves in the direction of the reviewer comments where appropriate. While the project remains large (in budget) and long (in time frame), they have nodded to the need to produce some products as early as possible, and I believe they will do this. They demonstrate meaningful linkages with VA leaders, through their advisory panel and by having recently joined some key efforts like the VA Choosing Wisely group (Dr. Kerr). These connections will ensure that the findings will have an impact on VA practice.

7. Responsiveness to Research Priorities or Special Solicitations.

This proposal is highly responsive to the RFA HX15-023. The potential of this proposal to add overtreatment measures to our regime of performance measurement has the potential to help correct a one-sided and unbalanced system of measurement. The contributions to assessing the operating characteristics (reliability etc.) of the new measures also will help ensure that they are properly specified and minimize unintended consequences. These would appear to be important contributions that help address the third point of the RFA, namely “A third challenge is that our current performance measure system, and the underlying data, need substantial revisions if we are to accurately measure quality of care and promote improvement where it is most needed.”

8. Protection of Human Subjects from Research Risk.

Adequate/no concerns.

9. Inclusion of Women and Minorities in Research.

Adequate/no concerns.

10. Budget.

The budget is large, but a waiver has been obtained. High-cost items, such as multiple evidence syntheses and chart reviews, are appropriately justified and are necessary for the conduct of the research.

11. Overall Impression.

This proposal entails detailed, careful work to painstakingly define a new regime of performance measurement regarding overtreatment. This includes a thorough search for all appropriate measures, ratings of their priority, mini evidence syntheses, detailed examination of their operating characteristics with validation by chart review, and planning for effectively implementing them, which will incorporate input from clinicians, VA leaders, and patients.

Overall, this project promises to produce very useful information which can help VA to introduce such measures in a "smart" way that will improve care and not produce unintended consequences. While VA will need to act before this project is done, even so, VA's interim "actions" may go awry in various ways. The products of this grant are very likely to be useful when they do arrive. It is important to remember that when operations acts quickly and decisively, sometimes it works out well, and sometimes less so. Where are they going to turn for better ideas when parts of their plans don't work out so well?

12. Key Strengths.

1. Strong group of investigators with relevant experience and links to VA leaders
2. Very detailed plan for a very comprehensive effort, it seems they have thought of everything that needs to be done to "vet" these new measures in every important way
3. Likely to make an important contribution to improving VA care

13. Key Weaknesses.

1. Remains a large project with a big budget, long timeline, and many moving parts. Sometimes that is just what is needed however, and this project is addressing an ambitious goal.

CRITIQUE 3

1. Significance.

This is a resubmission. The proposal was of great significance before, and continues to be so. The concept of de-intensification of care, i.e., when to dial back treatment that has been recommended for a patient, is one that can lead to reduced over treatment and its associated costs, better patient satisfaction, and most importantly, better quality of care. This proposal addresses a very important need that of identifying clinical conditions suitable for de-intensification guidelines, and creating guidelines and performance measures for de-intensification of treatment for the identified clinical conditions. The need for such work is not only this reviewer's opinion, it is echoed by the various members of the investigators' advisory board, including Dr. Joseph Francis, Director of Clinical Reporting at the VA Office of Analytics and Business Intelligence, whose letter of support is extremely favorable.

2. Approach.

The investigators propose high quality, well thought out methods. Their approach could be strengthened, however, in the following areas:

A. Conceptual model: although the authors cite Glasgow et al. ,and the Theoretical Domains Framework, the conceptual model as shown in the proposal is not congruent with what is presented in the citations they provide. It is not clear, therefore, how they arrived at the model in the form presented in the proposal

B. Pritchard and Ashwood expectancy theory and PRoMES framework: although there is a motivational theory posited in the Pritchard and Ashwood book cited, the theory is actually based on the NPI theory of motivation, and is correctly attributed to Naylord, Pritchard and Ilgen, not Pritchard and Ashwood. Similarly, although ProMES is built based on the NPI theory of motivation, ProMES itself is not a motivational framework, but rather a methodology for creating performance measures. It is therefore unclear how ProMES (despite the fact that I am a big fan of the methodology) will help in their efforts.

C. Multilevel analysis of specified indications by research questions of interest: the investigators state they will conduct “a variety of quantitative analyses”, but do not specify beyond that which analyses they plan on for that specific research question (p. 31).

3. Impact and Innovation.

Although the methods used are not particularly innovative, the proposed area of study is innovative and impactful. Measurement development requires a practiced hand with well-established methodologies to ensure validity and reliability. The proposed work uses not only tried and true methods, but also a team experienced at those methods.

4. Investigator Qualifications; Facilities and Resources.

This is a highly experienced, qualified, and well-resourced team. The proposed work draws directly from their experience in hue heir prior work. No concerns.

5. Multiple PI Leadership Plan.

Drs. Kerr and Hofer have complementary but unique skill sets. The proposed plan for distributing leadership of the three aims is appropriate, especially given that multiple parts of each aim are occurring simultaneously.

6. Adequacy of Response to Previous Feedback Provided by HSR&D Regarding the Proposed Study.

Investigators were very responsive to previous review, addressing each major concern adequately. In doing so, however, a few areas that were clear in the first submission became a little less clear in their presentation. For example, the investigators state:

“Most quality improvement approaches (e.g., LEAN, PDSA, Six sigma), acknowledge that measurement is an integral component of the organizational learning and engagement process. Therefore, our measurement procedure must consider not only the properties of measures themselves, but also the procedure necessary to make them reliable tools within a learning healthcare system. Yet, many factors, often unacknowledged, can undermine operational measurement of health care quality and there are some that are likely to be particularly salient when measuring and motivating de-intensification.”
This is new text, yet the point they are trying to get across, especially in the context of the proposal, is not clear. On the positive side, the investigators have successfully addressed concerns about the timeliness and appropriateness of their work given current measurement initiatives within VA.

7. Protection of Human Subjects from Research Risk.

No concerns.

8. Inclusion of Women and Minorities in Research.

Not applicable.

9. Budget.

The budget exceeds the established limits for IIRs. However they have obtained a waiver of this budget cap from Dr. David Atkins. Although the budget request is ambitious, it has been well justified in the proposal. The large amount of personnel proposed is to be able to complete multiple aims simultaneously in order to get results to the field as quickly as possible.

10. Overall Impression.

This resubmission continues to be highly impactful, needed work; the investigators have clearly explained how their proposed work fits within the larger context of VA's performance measurement initiatives and quality of care strategy.

11. Key Strengths.

1. Highly impactful, needed work with an important research question

2. Experienced, well-resourced team

3. Team builds effectively on their prior research.

4. Methods and approach are appropriate.

12. Key Weaknesses.

1. Budget exceeds cap (though waiver was obtained, and budget was justified)

2. Many moving parts and personnel, albeit necessary. Even with the best of teams, a lot of room for potential problems. .

MEETING ROSTER

Health Care System Organization and Delivery
Health Services Research Parent IRG
Office of Research & Development
Health Care System Organization and Delivery - Special Panel
HS5A R
August 25, 2015 - August 26, 2015

CHAIRPERSON

LOWERY, JULIE C., PHD
ASSOCIATE DIRECTOR
ANN ARBOR VA HSR&D CENTER OF INNOVATION
RESEARCH INVESTIGATOR
DEPARTMENT OF SURGERY
UNIVERSITY OF MICHIGAN
ANN ARBOR, MI 48105

MEMBERS

BENNETT, CHARLES L., MD, PHD
DIRECTOR, CENTER OF MEDICATION SAFETY
ENDOWED CHAIR IN MEDICATION SAFETY AND EFFICACY
SOUTH CAROLINA CENTERS OF ECONOMIC EXCELLENCE
PROFESSOR
UNIVERSITY OF SOUTH CAROLINA
COLUMBIA, SC 29208

CHARLTON, MARY E. PHD *
CORE INVESTIGATOR
IOWA CITY VA HSR&D CENTER OF INNOVATION
CLINICAL ASSISTANT PROFESSOR
DEPARTMENT OF EPIDEMIOLOGY
UNIVERSITY OF IOWA COLLEGE OF PUBLIC HEALTH
IOWA CITY, IA 52246

CHOU, ANN F, PHD *
ASSOCIATE PROFESSOR
DEPARTMENT OF HEALTH ADMINISTRATION AND POLICY
DEPARTMENT OF FAMILY AND PREVENTIVE MEDICINE
COLLEGES OF PUBLIC HEALTH AND MEDICINE
UNIVERSITY OF OKLAHOMA
OKLAHOMA CITY, OK 73117

COWPER RIPLEY, DIANE C., PHD *
CO-SITE DIRECTOR
RESEARCH SERVICE
GAINESVILLE VA MEDICAL CENTER
GAINESVILLE, FL 32608

HALL, DANIEL E, MD *
RESEARCH HEALTH SCIENTIST
PITTSBURGH VA MEDICAL CENTER
ASSOCIATE PROFESSOR
UNIVERSITY OF PITTSBURGH
PITTSBURGH, PA 15213

HOGAN, TIMOTHY PATRICK PHD *
RESEARCH HEALTH SCIENTIST
BOSTON/BEDFORD VA HSR&D CENTER OF INNOVATION
ASSISTANT PROFESSOR
DEPARTMENT OF QUANTITATIVE HEALTH SCIENCES
UNIVERSITY OF MASSACHUSETTS MEDICAL SCHOOL
BEDFORD, MA 01730

HYSONG, SYLVIA J., PHD *
RESEARCH HEALTH SCIENTIST
HOUSTON VA HSR&D CENTER OF INNOVATION
ASSOCIATE PROFESSOR
DEPARTMENT OF MEDICINE
BAYLOR COLLEGE OF MEDICINE
HOUSTON, TX 770304211

KABOLI, PETER J., MD *
CHIEF OF MEDICINE
IOWA CITY VA HSR&D CENTER OF INNOVATION
PROFESSOR
DEPARTMENT OF GENERAL INTERNAL MEDICINE
UNIVERSITY OF IOWA CARVER COLLEGE OF MEDICINE
IOWA CITY, IA 52246

LUTHER, STEPHEN L, PHD
DIRECTOR
METHODOLOGY CORE
CO-DIRECTOR
INFORMATICS AND MEASUREMENT FOCUS AREA
TAMPA/GAINESVILLE VA HSR&D CENTER OF INNOVATION
TAMPA, FL 33637

OLSEN, MAREN K. PHD *
DIRECTOR OF HSR BIOSTATISTICS UNIT
DURHAM VA HSR&D CENTER OF INNOVATION
ASSOCIATE PROFESSOR
DEPARTMENT OF BIOSTATISTICS AND BIOINFORMATICS
DUKE UNIVERSITY
DURHAM, NC 27710

PHIBBS, CIARAN S., PHD *
ASSOCIATE DIRECTOR
HEALTH ECONOMICS RESOURCE CENTER
PALTO ALTO VA MEDICAL CENTER
RESEARCH ASSOCIATE
STANFORD UNIVERSITY
MENLO PARK, CA 94025

ROSE, ADAM J. MD *
STAFF PHYSICIAN
BOSTON VA MEDICAL CENTER
ASSISTANT PROFESSOR OF MEDICINE
BOSTON UNIVERSITY SCHOOL OF MEDICINE
BOSTON, MA 02130

SINGH, HARDEEP , MD *
PHYSICIAN
HOUSTON VA MEDICAL CENTER
ASSOCIATE PROFESSOR
SECTION OF HEALTH SERVICES RES & GEN INTERNAL MED
BAYLOR COLLEGE OF MEDICINE
HOUSTON, TX 77030

SINGH, JASVINDER A, MBBS *
STAFF RHEUMATOLOGIST
BIRMINGHAM VA MEDICAL CENTER
ASSOCIATE PROFESSOR
DEPTS OF MEDICINE AND EPIDEMIOLOGY
UNIVERSITY OF ALABAMA AT BIRMINGHAM
BIRMINGHAM, AL 35233

SOLIMEO, SAMANTHA , PHD *
INVESTIGATOR & HEALTH SCIENCE RESEARCH SPECIALIST
IOWA CITY VA HSR&D CENTER OF INNOVATION
ASSISTANT PROFESSOR
DEPARTMENT OF GENERAL INTERNAL MEDICINE
UNIVERSITY OF IOWA
IOWA CITY, IA 52246

TRIVEDI, AMAL N., MPH, MD *
RESEARCH HEALTH SCIENTIST
PROVIDENCE VA MEDICAL CENTER
ASSOCIATE PROFESSOR
DEPARTMENT OF HEALTH SERVICES, POLICY, & PRACTICE
BROWN UNIVERSITY
PROVIDENCE, RI 02912

VOGELI, CHRISTINE PHD *
DIRECTOR OF EDUCATION
MONGAN INSTITUTE FOR HEALTH POLICY
MASSACHUSETTS GENERAL HOSPITAL
ASSISTANT PROFESSOR MEDICINE
HARVARD MEDICAL SCHOOL
BOSTON, MA 02114

WHITTLE, JEFFREY C., MD
DIRECTOR
HEALTH SERVICES RESEARCH & DEVELOPMENT
MILWAUKEE VA MEDICAL CENTER
PROFESSOR OF GENERAL INTERNAL MEDICINE
MEDICAL COLLEGE OF WISCONSIN
MILWAUKEE, WI 53295

MAIL REVIEWER(S)

ELWY, ANASHUA RANI, PHD
RESEARCH SCIENTIST
DIRECTOR, CIDER
BOSTON/BEDFORD VA HSR&D CENTER OF INNOVATION
ASSISTANT PROFESSOR
BOSTON UNIVERSITY SCHOOL OF PUBLIC HEALTH
BEDFORD, MA 01730

SCIENTIFIC REVIEW OFFICER

BANERJEA, RANJANA PHD
DEPARTMENT OF VETERANS AFFAIRS
VETERANS HEALTH ADMINISTRATION
OFFICE OF RESEARCH AND DEVELOPMENT
HEALTH SERVICES RESEARCH & DEVELOPMENT
WASHINGTON, DC 20420

* Temporary Member. For grant applications, temporary members may participate in the entire meeting or may review only selected applications as needed.

Consultants are required to absent themselves from the room during the review of any application if their presence would constitute or appear to constitute a conflict of interest.
